# Phenyl(thio)phosphon(amid)ate Benzenesulfonamides as Potent and Selective Inhibitors of Human Carbonic Anhydrases II and VII Counteract Allodynia in a Mouse Model of Oxaliplatin-Induced Neuropathy

**DOI:** 10.1021/acs.jmedchem.9b02135

**Published:** 2020-05-04

**Authors:** Alessio Nocentini, Vincenzo Alterio, Silvia Bua, Laura Micheli, Davide Esposito, Martina Buonanno, Gianluca Bartolucci, Sameh M. Osman, Zeid A. ALOthman, Roberto Cirilli, Marco Pierini, Simona Maria Monti, Lorenzo Di Cesare Mannelli, Paola Gratteri, Carla Ghelardini, Giuseppina De Simone, Claudiu T. Supuran

**Affiliations:** † Department of NEUROFARBA, Pharmaceutical and Nutraceutical Section, University of Florence, Via Ugo Schiff 6, 50019 Sesto Fiorentino, Italy; ‡ Istituto di Biostrutture e Bioimmagini, CNR, Via Mezzocannone 16, 80134 Napoli, Italy; § Department of NEUROFARBA, Pharmacology and Toxicology Section, University of Florence, Viale Pieraccini 6, 50139 Firenze, Italy; ∥ Chemistry Department, College of Science, King Saud University, P.O. Box 2455, Riyadh 11451, Saudi Arabia; ⊥ Centro nazionale per il controllo e la valutazione dei farmaci, Istituto Superiore di Sanità, Viale Regina Elena 299, 00161 Rome, Italy; # Dipartimento di Chimica e Tecnologie del Farmaco, Sapienza University of Rome, P.le A. Moro 5, 00185 Rome, Italy

## Abstract

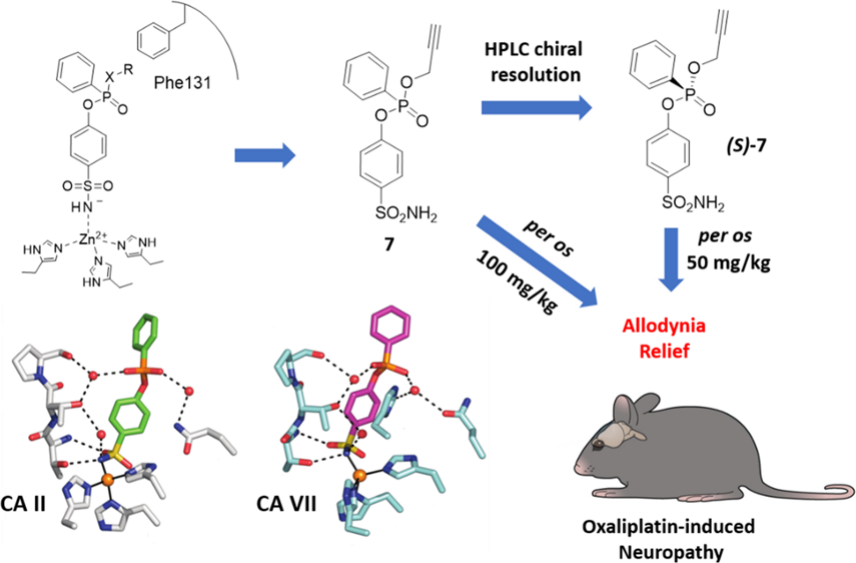

Human carbonic anhydrase (CA; EC 4.2.1.1) isoforms II and VII are implicated in neuronal excitation, seizures, and neuropathic pain (NP). Their selective inhibition over off-target CAs is expected to produce an anti-NP action devoid of side effects due to promiscuous CA modulation. Here, a drug design strategy based on the observation of (dis)similarities between the target CA active sites was planned with benzenesulfonamide derivatives and, for the first time, a phosphorus-based linker. Potent and selective CA II/VII inhibitors were identified among the synthesized phenyl(thio)phosphon(amid)ates **3**–**22**. X-ray crystallography depicted the binding mode of phosphonic acid **3** to both CAs II and VII. The most promising derivatives, after evaluation of their stability in acidic media, were tested in a mouse model of oxaliplatin-induced neuropathy. The most potent compound racemic mixture was subjected to HPLC enantioseparation, and the identification of the eutomer, the (*S*)-enantiomer, allowed to halve the dose totally relieving allodynia in mice.

## Introduction

Neuropathic pain (NP) is pain initiated by a damage or ailment of the peripheral or central somatosensory system.^1^ The prevalence of NP in the population is estimated to be 6.9–10% and amounts to 20–25% of all chronic pain cases.^2,3^ Numbness, needle sensation and tingling, paresthesia, and neurological sensory deficits are NP main symptoms, which can occur both at the central and peripheral nervous systems. Peripheral neuropathies can derive from viral infections and traumatic, post-surgical, or diabetic neuropathies, while central pain syndromes are usual in patients who suffered from multiple sclerosis, spinal cord injury, or stroke.^4^ The syndromes can manifest in the form of spontaneous, continuous (or paroxysmal), or evoked pain. The latter can be in turn distinguished in hyperalgesia, in case it is initiated by painful stimuli and allodynia, if triggered by non-noxious stimuli. Neuropathic pain is challenging to treat because of the heterogeneity and complexity of signals and symptoms. Indeed, the use of traditional analgesics (e.g., nonsteroidal anti-inflammatory drugs) or weak opioids is associated to weak outcomes in NP patients as these drugs do not target the types of symptoms correlated with the disease.^4^ The lack of specific drugs for treating NP is partially overcome with serendipitously discovered agents of the anticonvulsant and antidepressant classes, although actually only 40–60% of patients attain satisfactory pain alleviation upon treatment.^3^ The first-line treatment comprises some types of antidepressants such as tricyclic antidepressants, dual norepinephrine/serotonin reuptake inhibitors, topical analgesics (lidocaine), and calcium channel α2-δ ligands as anticonvulsants (pregabalin and gabapentin).^1,5−7^ Second-line treatments are represented by effective opioids and tramadol, while other antidepressants, antiepileptics (among which topiramate, zonisamide, and lacosamide), *N*-methyl-d-aspartate receptor antagonists, topical capsaicin, and mexiletine can be used as third-line treatment.^8−10^ Alternative pharmacological strategies are being actually explored to overcome the difficulties met in the treatment of most NP patients. Among the numerous promising new targets/drug classes identified are histamine H4 receptor (H4R) agonists, agonists of type 1 lysophosphatidic acid receptor, glial cell activity modulators, μ-opioid receptor (MOP) agonists, and carbonic anhydrase inhibitors (CAIs).^11−14^

Carbonic anhydrases (CAs; EC 4.2.1.1) are widely expressed in the central nervous system (CNS) and choroid plexus.^15,16^ Of the 15 human (h) CAs, CA II is the most physiologically relevant isoform and is abundantly expressed in oligodendrocytes, myelin sheaths, astrocytes, choroid plexus, and myelinated tracts of brain.^15,17^ The membrane-bound CA IV is located in layers III and VI in thalamus, hippocampus, and cortex as well as on the luminal surface of cerebral capillaries present in the blood–brain barrier.^18^ Likewise, CA VII is highly expressed in thalamus, hippocampus, and cortex. CA VII is deemed a brain-associated CA isozyme as it is predominantly expressed in the CNS while missing in most other systems.^15,18^

The link between CAs and NP was recently established by the groups of Kaila and Price.^19,20^ The CA-catalyzed production of HCO_3_^–^ significantly affects the biochemistry and physiology of neurotransmitters, such as γ-aminobutyric acid (GABA). Injuries in peripheral nerves negatively impact spinal GABAergic networks by reducing the neuron-specific potassium chloride (K^+^–Cl^–^) cotransporter (KCC2). A potential strategy to mitigate this process was validated, which implicates CA inhibition as it can decrease bicarbonate-related depolarization through GABA_A_ receptors in case the KCC2 function is compromised. Moreover, Ruusuvuori et al. have provided insights into the role of CAs in the modulation of the neuronal signaling and showed that isoforms II and VII are the only cytosolic isozymes present in both somata and dendrites of mature CA1 pyramidal neurons.^21,22^ As CAs are implicated in maintaining the bicarbonate gradient that results in the efflux of HCO_3_^–^ ions through GABA_A_ receptors, this study corroborated the involvement of CA VII as well as CA II in neuronal excitation and seizures.^21^

Hence, a structure-based drug design strategy based on benzenesulfonamide derivatives including, for the first time, a phosphorus-based linker is here reported to provide potent and possibly selective CA II/VII inhibitors in search of new tools for the management of neuropathic pain.

## Results and Discussion

### Drug Design and Chemistry

The spreading interest of novel therapeutic agents based on CA inhibition or continued validation of specific hCAs as drug targets requires that selective inhibitors are developed.^23^ However, all clinically used CAIs, among which primary sulfonamides and sulfamates investigated in the therapy of neuropathic pain such as acetazolamide, methazolamide, topiramate, zonisamide, celecoxib, and furosemide, are predominantly nonselective with respect to all human isoforms.^17^ Most recent drug design studies on CAIs involved the tumor-associated CA IX and CA XII and culminated with the entrance of the phenylureidobenzenesulfonamide **SLC-0111** in phase Ib/II clinical trials for the treatment of advanced, metastatic solid tumors as a monotherapy or in combination with other agents such as gemcitabine.^23,24^ In contrast, few compounds with pronounced inhibitory selectivity and/or potency toward hCA II and hCA VII have been reported to date.^23,25^

**Figure 1:**
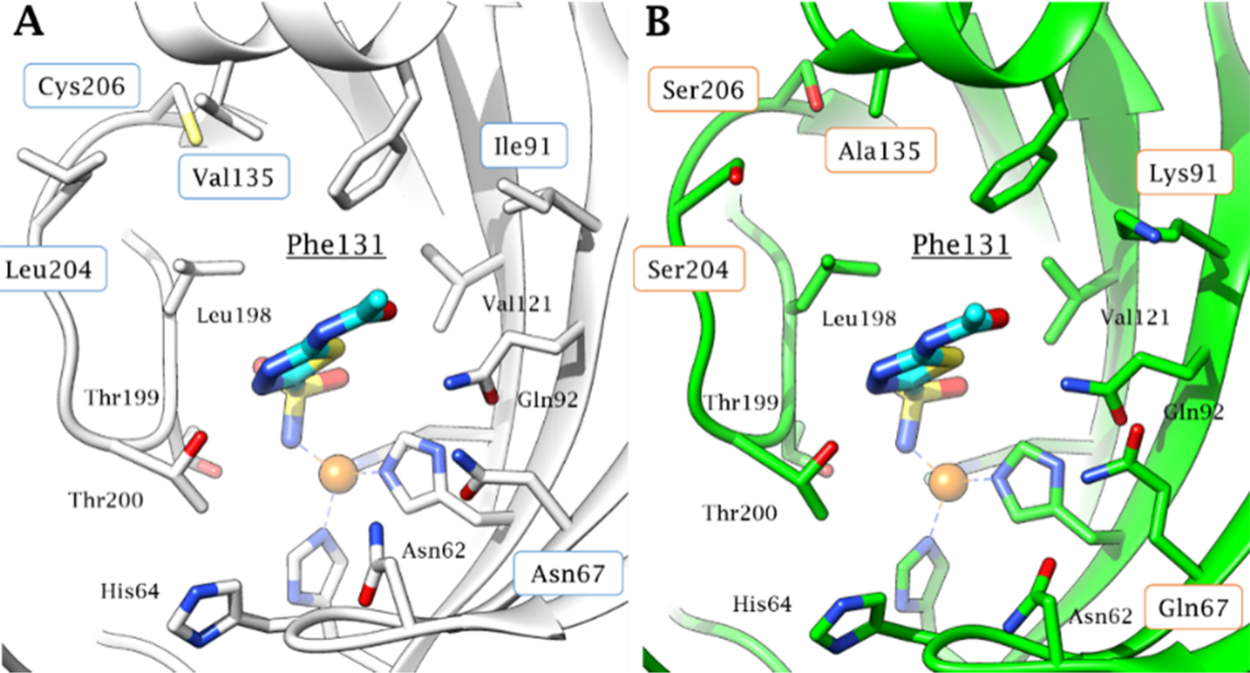
Comparison between the active site of (A) hCA II (PDB ID: 3HS4) and (B) hCA VII (PDB ID: 3ML5) in complex with acetazolamide (**AAZ**). Residues different between the two enzymes are boxed in blue for hCA II and orange for hCA VII. The target amino acid Phe131 from both active sites is underlined.

In this study, by observing the significant similarity in amino acid composition and active site architecture existing between hCA II and hCA VII (Figure 1), a new series of CAIs were designed, which can concomitantly target both isozymes with the aim of achieving a neuropathic pain-relieving action.^15,26^

**Figure 2:**
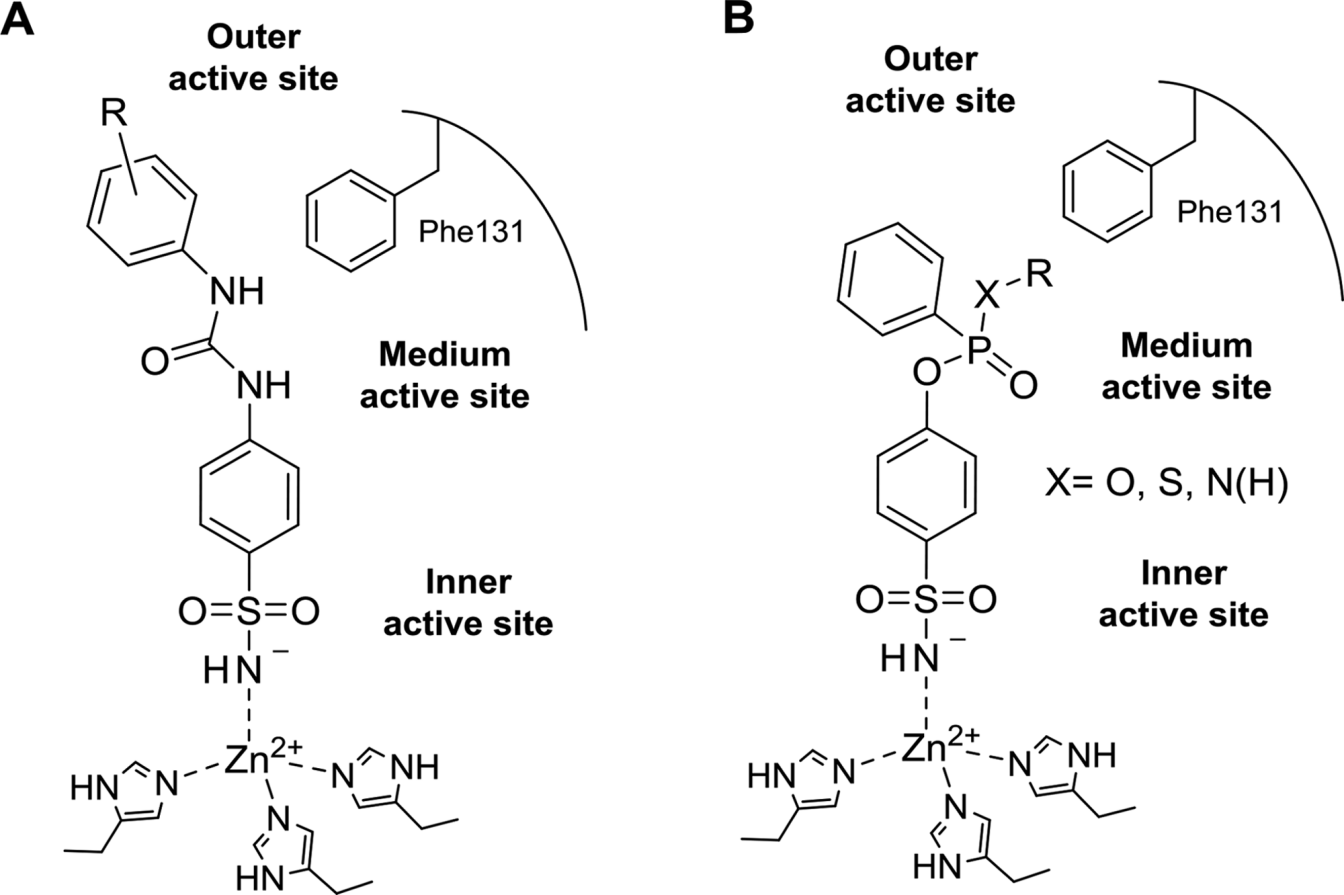
(A) Schematic representation of phenylureidobenzenesulfonamides bound to the CA II active site. (B) Drug design of benzene (thio)phosphon(amid)ates as CA II and VII inhibitors.

In detail, CA II and CA VII share a Phe residue in position 131 (Figure 1). This residue is known to play an important role in the binding of many CAIs^26,27^ and thus was taken as the reference point to explore the chemical space in the middle portion of the CA active site where differences exist between CAs II and VII (Figure 2) as well as with other CA isoforms. Benzenesulfonamide derivatives were thus designed according to the tail approach that, in recent years, has been mainly applied by using urea-based linkers.^23,26^ Such an approach consists in appending a variety of chemical frameworks to the main CA inhibitory scaffold with the aim of diversely targeting the medium/outer regions of the CA active sites where most amino acid variability exists.^23^ Nonetheless, the arylureidobenzenesulfonamide (Figure 2A) was not deemed to possess an easily accessible chemical versatility to include a variety of substituents for studying the binding to the active site middle region, that is, nearby Phe131. In fact, such a scaffold has been chiefly adopted for exploring the chemical space in the outer region of the binding cavity.^28,29^ Hence, in this context a phosphorus-based linker (Figure 2B) was adopted for the following reasons:^30^ (i) because of the electronic properties of the phosphorus atom, an additional substituent can be included on the spacer while holding the benzenesulfonamide and the aromatic ring interacting with Phe131 (i.e., the outer ring of the benzenesulfonamide derivative in Figure 2B); (ii) the extra substituent is appended in the middle of the derivative structure, a suitable position for interaction with the medium portion of the binding cavity (Figure 2B); (iii) by using phenylphosphonic dichlorides as reagents, a wide variety of substituents can be easily appended to the spacer in the form of (thio)phosphon(amid)ates; and (iv) finally, the presence of a chiral center on the phosphorus atom could induce an enantiomeric binding mode.

**Scheme 1:**
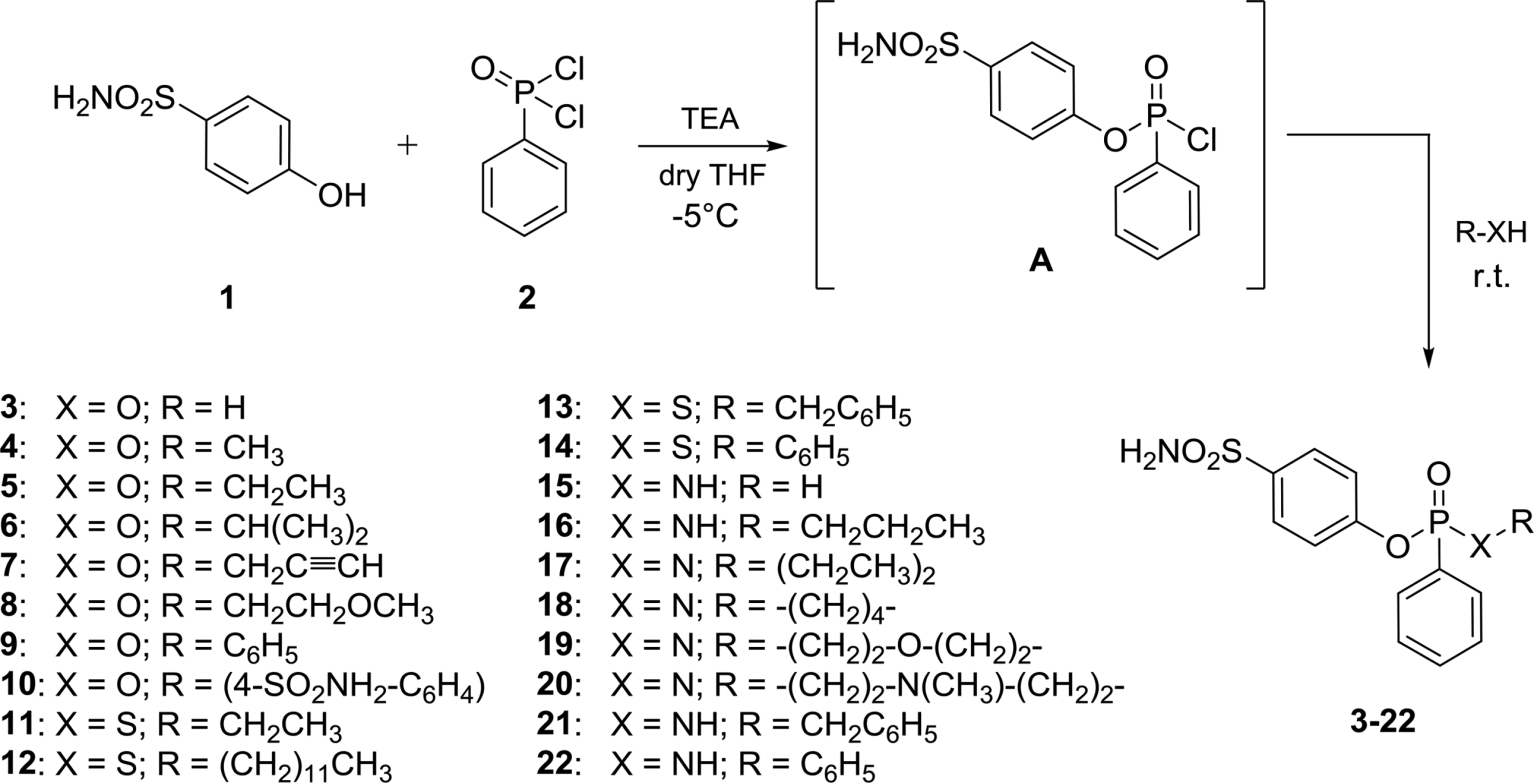
Synthesis of (Thio)phosphonates **3**–**14** and Phosphonamidates **15**–**22**

On the basis of the drug design, a series of (thio)phosphon(amid)ates were designed and synthesized using 4-hydroxybenzenesulfonamide **1** and phenylphosphonic dichloride **2** as starting materials (Scheme 1). The choice of **1** in place of sulfanilamide, which is commonly derivatized to yield benzenesulfonamide series, was related to its major reactivity with phosphonic chlorides than the aromatic amine, thus producing higher yields as well as ease of purification.^31^ The snap addition of phenylphosphonic dichloride **2** to a solution of 4-hydroxybenzenesulfonamide **1** and triethylamine in anhydrous THF at −5 °C yielded intermediate **A**. In contrast, a greater rate of double attack of **1** into phenylphosphonic dichloride was observed raising the temperature over r.t., and thus, these conditions were used to achieve compound **10**. Otherwise, the addition of various nucleophiles, among which (thio)alcohols, (thio)phenols, and aliphatic and aromatic amines, to the reaction mixture containing **A** and stirring thereof at r.t. yielded (thio)phosphonates **3**–**9** and **11**–**14** and phosphonamidates **15**–**22** (Table 1). The adopted synthetic pathway provided the compounds as racemic mixtures, with the exception of bis-sulfonamide **10** and, when deprotonated, phenylphosphonic acid **3**. All derivatives were purified by column chromatography using silica gel and MeOH/DCM gradients and fully characterized by ^1^H NMR, ^13^C NMR, ^31^P NMR, and HRMS (Supporting Information). According to literature data, peak picking of ^31^P signals in NMR spectra reported the various ranges of signals given by phosphorus atoms of different types. ^31^P signals of alkyl phosphonates **3**–**8** spanned from 14.31 to 16.73 ppm, while those of phosphonic acid **3** and aryl phosphonates **9** and **10** lied in the range 12.01–12.49 ppm. In contrast, ^31^P signals of alkyl thiophosphonates **11**–**13** were found between 42.64 and 43.65 ppm, whereas the thiophenol derivative **14** showed a signal at 39.97 ppm. Primary and secondary phosphonamidates **15**–**17** and **21** reported ^31^P peaks in the range 21.20–22.23 ppm, the tertiary derivatives **18**–**20** between 18.85 and 19.65 ppm, and the aromatic amine derivative at 15.37 ppm.

**Table 1:** Inhibition Data of Human CA Isoforms I, II, IV, VA, VB, VII, IX, and XII with (Thio)phosphon(amid)ates **3**–**22** and the Standard Acetazolamide (**AAZ**) by a Stopped-Flow CO_2_ Hydrase Assay^32^

	*K*_I_^a^ (nM)							
cmp	CA I	CA II	CA IV	CA VA	CA VB	CA VII	CA IX	CA XII
**3**	196.9 ± 11.2	37.9 ± 2.1	220.1 ± 16.2	76.3 ± 5.2	52.8 ± 3.4	16.1 ± 1.1	26.1 ± 1.4	30.8 ± 2.3
**4**	494.9 ± 35.4	11.4 ± 0.8	259.2 ± 14.5	51.7 ± 4.0	24.3 ± 1.5	36.0 ± 1.9	16.2 ± 0.9	47.2 ± 3.1
**5**	397.5 ± 26.7	36.6 ± 2.4	424.6 ± 35.6	142.6 ± 8.3	40.1 ± 2.6	38.2 ± 2.5	18.7 ± 1.1	22.4 ± 1.5
**6**	307.8 ± 18.1	12.8 ± 0.7	1196 ± 102	100.8 ± 5.9	19.8 ± 1.1	6.2 ± 0.4	17.5 ± 1.2	42.0 ± 3.2
**7**	415.3 ± 29.0	3.6 ± 0.2	1322 ± 124	82.1 ± 5.7	38.4 ± 2.5	12.2 ± 0.8	22.6 ± 1.6	26.7 ± 1.8
**8**	779.7 ± 44.8	28.5 ± 1.9	1644 ± 113	59.3 ± 4.1	67.2 ± 3.8	25.0 ± 1.2	12.1 ± 0.7	35.7 ± 2.8
**9**	971.1 ± 62.7	15.2 ± 1.0	2619 ± 153	470.4 ± 33.6	22.7 ± 1.4	31.4 ± 1.8	13.2 ± 0.9	24.6 ± 1.9
**10**	670.7 ± 40.1	3.2 ± 0.2	360.3 ± 29.5	284.3 ± 19.3	15.0 ± 0.9	7.7 ± 0.5	19.7 ± 1.4	16.9 ± 0.9
**11**	726.7 ± 49.3	30.2 ± 2.1	1389 ± 89	94.3 ± 5.8	96.7 ± 5.7	41.5 ± 2.8	27.0 ± 2.0	34.1 ± 2.5
**12**	8345 ± 552	234.1 ± 15.3	4164 ± 342	641.9 ± 45.7	224.6 ± 13.4	100.8 ± 6.3	51.2 ± 3.6	47.8 ± 3.4
**13**	3264 ± 258	64.8 ± 3.7	4456 ± 298	150.7 ± 10.4	58.4 ± 4.1	75.5 ± 5.7	25.0 ± 1.6	21.3 ± 1.6
**14**	1635 ± 101	34.9 ± 2.3	2633 ± 186	382.0 ± 26.5	28.7 ± 1.6	15.7 ± 1.2	6.6 ± 0.4	39.2 ± 2.5
**15**	195.1 ± 15.7	17.9 ± 1.0	292.4 ± 22.1	41.3 ± 3.2	12.6 ± 0.8	18.9 ± 1.0	20.9 ± 1.2	15.4 ± 0.8
**16**	759.4 ± 57.2	26.2 ± 1.6	1516 ± 99	85.2 ± 6.2	33.7 ± 2.4	20.8 ± 1.4	3.2 ± 0.2	17.3 ± 0.9
**17**	521.3 ± 37.5	17.5 ± 1.1	371.1 ± 26.4	106.8 ± 5.9	49.1 ± 2.7	13.5 ± 0.8	19.5 ± 1.4	46.8 ± 3.1
**18**	242.1 ± 18.6	1.7 ± 0.1	307.6 ± 19.2	78.3 ± 4.7	25.6 ± 1.3	9.3 ± 0.6	28.9 ± 2.1	58.1 ± 4.5
**19**	317.9 ± 17.0	7.9 ± 0.5	120.1 ± 8.3	43.9 ± 3.1	27.4 ± 1.6	30.5 ± 2.4	11.1 ± 0.6	27.6 ± 1.3
**20**	390.8 ± 29.6	9.1 ± 0.6	76.9 ± 5.2	164.1 ± 12.5	47.3 ± 3.4	17.0 ± 1.3	61.9 ± 5.1	25.3 ± 1.4
**21**	1705.9 ± 117	52.9 ± 2.9	643.9 ± 46.2	59.1 ± 4.0	68.7 ± 4.8	15.4 ± 1.0	2.6 ± 0.2	19.7 ± 1.2
**22**	817.9 ± 62.4	2.4 ± 0.1	859.9 ± 58.3	184.6 ± 14.1	24.0 ± 1.5	5.5 ± 0.3	19.6 ± 1.1	20.9 ± 1.3
**AAZ**	250 ± 15	12 ± 1	74 ± 6	63 ± 4	54 ± 3	2.5 ± 0.2	25 ± 2	5.7 ± 0.5

a Inhibition data are expressed as means ± SEM of three different assays.

### Carbonic Anhydrase Inhibition

(Thio)phosphon(amid)ates **3**–**22** were evaluated for their inhibition against the cytosolic CAs I, II, and VII, the mitochondrial CAs VA and VB, and the membrane-associated CAs IV, IX, and XII by a stopped-flow method that measures CO_2_ hydrase activity.^32^**AAZ** was used as the reference drug. The following SAR can be worked out from the data reported in Table 1.

Overall, the adopted structure-based drug design strategy produced a satisfactory result in terms of inhibition potency against the target CAs II and VII as well as selectivity over the other ubiquitous and off-target isoform CA I. The latter isozyme was in fact feebly inhibited by (thio)phosphon(amid)ates **3**–**22** with *K*_I_ values (inhibition constants) spanning in the high nanomolar up to low micromolar range (195.1–8345 nM). Phosphonic acid **3** and primary phosphonamidate **15** stood out as the most potent CA I inhibitors presumably because of the low steric hindrance produced within one of the narrowest active sites among hCAs. As a matter of fact, compounds bearing phenyl (**9**, **14**, and **22**) and benzyl (**13** and **21**) R groups as well as the lauryl derivative **12** acted as the weakest CA I inhibitors (*K*_I_ values of 817.9–8345 nM).

The target CA II was effectively inhibited by compounds **3**–**22** with *K*_I_ values below 100 nM (*K*_I_ values of 1.7–64.8 nM), except for derivative **12**, which showed again the lowest CA inhibition (a *K*_I_ of 234.1 nM). *K*_I_ values even below 10 nM were exhibited by propargyl and bis-sulfamoyl phosphonates **7** and **10** and phosphonamidates bearing a pyrrolidine (**18**), morpholine (**19**), *N*-methylpiperazine (**20**), and aniline (**22**). It should be noted that all other compounds inhibit CA II with *K*_I_ values below 50 nM with the exceptions of the benzyl derivatives **13** and **21** (*K*_I_ values of 64.8 and 52.9 nM).

SAR of compounds **3**–**22** against CA IV is rather flat. This isozyme was the least inhibited by the new reported derivatives, most of which did not inhibit CA IV up to 500 nM. *K*_I_ values lying in the range 120–450 nM were however estimated for phosphonates **3**–**5** and **10** and phosphonamidates **15** and **17**–**19**, whereas *N*-methylpiperazine **20** uniquely achieved a *K*_I_ of 76.9 nM.

The mitochondrial CAs VA and VB show two distinct levels of inhibition with compounds **3**–**22**. While CA VB was inhibited in a comparable range with CA II (*K*_I_ values between 12.6 and 224.6 nM), a drop of inhibition was measured for CA VA (*K*_I_ values of 41.3–641.9 nM) but without approaching the high nanomolar to low micromolar *K*_I_ values detected against CAs I and IV. Methyl phosphonate **4** (a *K*_I_ of 51.7 nM), simple phosphonamidate **15** (a *K*_I_ of 41.3 nM), and morpholine derivative **19** (a *K*_I_ of 43.9 nM) stood out as the best CA VA inhibitors herein, whereas a drop of efficacy was consistently observed with thiophosphonates **12**–**14** (*K*_I_ values of 150.7–641.9 nM). Phosphonamidate **15** was also detected as the most effective inhibitor against CA VB (a *K*_I_ of 12.6 nM) in a comparable manner with bis-benzenesulfonamide **10** (a *K*_I_ of 15.0 nM). The aliphatic thiophosphonates **11** and **12** showed to act as the worst CA VB inhibitors with *K*_I_ values of 96.7 and 224.6 nM. The remaining compounds showed *K*_I_ values spanning in a rather narrow range between 20 and 60 nM.

As CA II, the other target isozyme CA VII was efficiently inhibited by compounds **3**–**22** as *K*_I_ values did not raise above 50 nM (spanning from 5.5 to 41.5 nM), with the exception of thiophosphonates **12** and **13** (*K*_I_ values of 100.8 and 75.5 nM). The isopropyl (**6**) and bis-sulfamoyl (**10**) compounds among phosphonates and pyrrolidine (**18**) and aniline (**22**) derivatives among phosphonamidates acted as the most potent CA VII inhibitors with *K*_I_ values below 10 nM.

Although not presenting a Phe residue in position 131, but instead a Val or Ala, respectively, the tumor-associated CAs IX and XII were potently inhibited by compounds **3**–**22**. In fact, these isozymes display two of the roomiest active sites among hCAs, which enable the accommodation and adjustment of a plethora of chemotypes and moieties from CAIs. Interactions with Phe131 might be indeed substituted by hydrophobic contacts with Val and Ala residues that summed up with other ligand/target hydrophobic interactions within the binding pocket account for low nanomolar inhibitory effectiveness. It should be stressed that, for instance, as the lauryl derivative **12**, the weak inhibitor against other screened isoforms showed a 50 nM efficacy against CAs IX and XII, owing to a likely favored adaptation within their binding sites, which show rather extended lipophilic pockets. CA IX and CA XII showed rather flat inhibition profiles with *K*_I_ values spanning in the ranges 2.6–61.9 and 15.4–58.1 nM.

Interestingly, a subset of derivatives can be selected, which showed a preferred inhibition against the target CAs II and VII over all other isoforms, particularly CAs I, IV, and VA: isopropyl (**6**), propargyl (**7**), and bis-sulfamoyl (**10**) phosphonates and phosphonamidates bearing pyrrolidine (**18**), *N*-methylpiperazine (**20**), and aniline (**22**). It is fair to stress that these compounds showed selectivity for CAs II and VII above one order of magnitude solely over CAs I, IV, and VA. In contrast, the selectivity index was lower for CAs II and VII over CAs VB, IX, and XII, which are, however, minor off-target isoforms with respect to the ubiquitous CA I.

### X-ray Crystallography

To understand the molecular basis responsible for the inhibition efficiency of phosphonate/phosphonamidate benzenesulfonamides against CA II and CA VII, the crystal structure of these two isoforms was solved in adduct with phenylphosphonic acid **3**. This compound based on the ChemAxon software^36^ has a p*K*_a_ value of 1.62 and, therefore, is assumed to bind the enzyme at physiological pH and in crystallization conditions in a deprotonated, nonchiral form. Crystals of the CA adducts with **3** were obtained by soaking experiments as previously reported for other sulfonamide CA inhibitors.^33−35^ Data were collected to resolution values of 1.26 and 1.94 Å for CA II/**3** and CA VII/**3** complexes, respectively. The structures were analyzed by difference Fourier techniques and refined to *R*_work_ and *R*_free_ values of 14.6 and 17.4% for the CA II/**3** complex and 18.8 and 22.6% for the CA VII/**3** one (Table S1). Analysis of the electron density maps at various stages of the crystallographic refinement showed in both complexes the binding of one molecule of the inhibitor within the enzyme active site.

**Figure 3:**
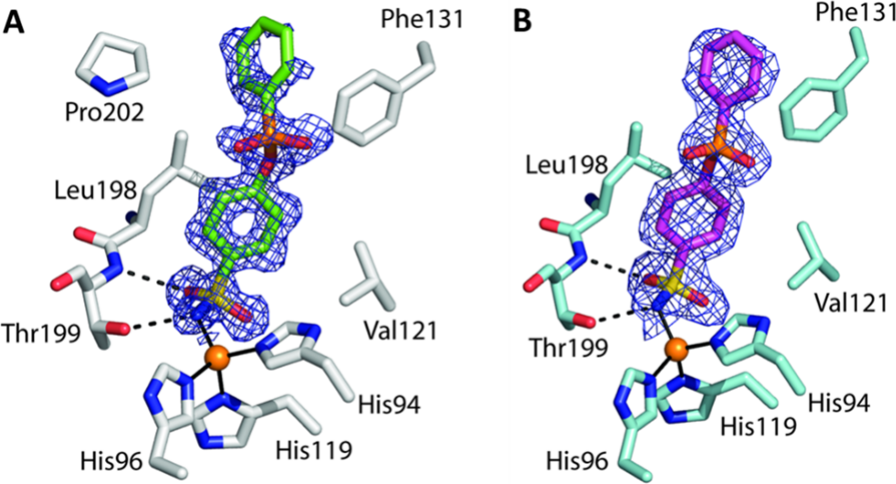
σA-weighted |2Fo – Fc| map (contoured at 1.0 σ) relative to the inhibitor molecule in (A) CA II/**3** and (B) CA VII/**3** adducts. The zinc ion coordination and residues with a distance of less than 4.0 Å from the inhibitor are also reported. Continuous lines show zinc ion coordination, whereas dashed lines indicate potential hydrogen bonds.

The electron density maps were very well defined for both proteins and for the entire inhibitor in the case of CA VII/**3**. On the contrary, in the case of the CA II/**3** complex, a less defined electron density was associated to the inhibitor phenyl tail, indicating a higher mobility of this part of the molecule within the CA II active site (Figure 3).

**Figure 4:**
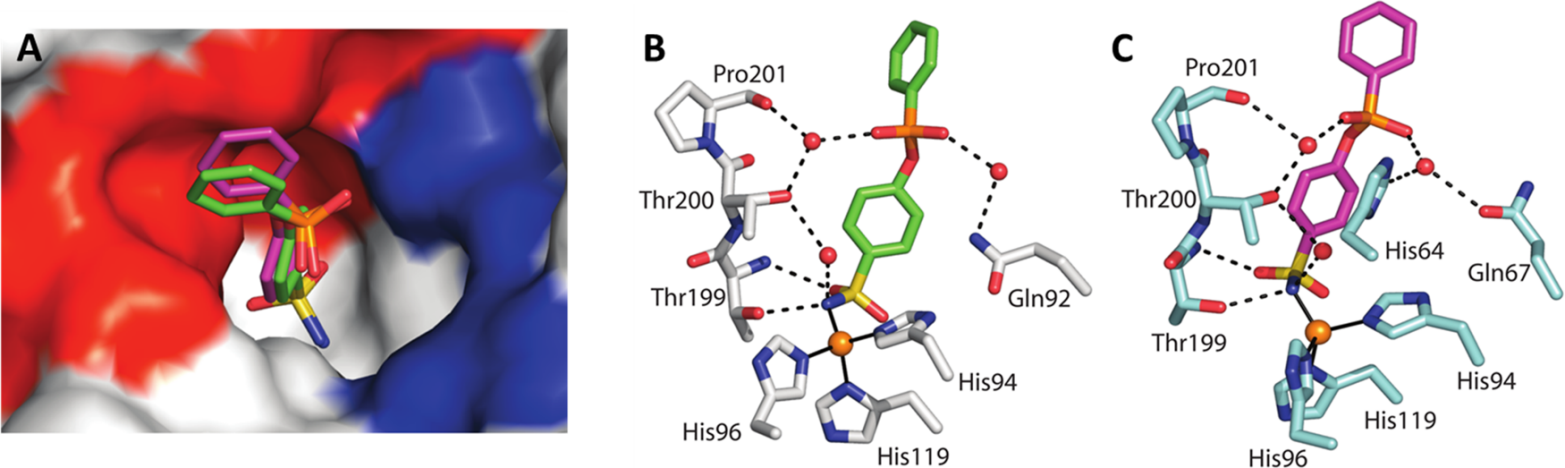
(A) Structural superposition of compound **3** when bound to CA II (green) with the same compound when bound to CA VII (magenta). The surface representation of CA II is also shown with the hydrophobic region of the active site in red and the hydrophilic one in blue. (B, C) Details of the polar interactions established by inhibitor **3** within the active site of (B) CA II and (C) CA VII. Water molecules are represented as red spheres, continuous lines indicate zinc ion coordination, whereas dashed lines indicate hydrogen bond distances.

As shown in the structural superposition reported in Figure 4A, the inhibitor adopts the same orientation within the two active sites, with only a small shift of the phenyl tail. In detail, in both structures, the benzenesulfonamide moiety binds the active site in the same conformation adopted in other CA/benzenesulfonamide complexes, coordinating the catalytic zinc ion, forming two hydrogen bonds with residue Thr199, and establishing many hydrophobic interactions with residues located on the bottom of the cavity (Figure 3 and Table S2). The phosphonate group points toward the hydrophilic region of the active site^13^ and forms in both structures several water-mediated hydrogen bonds with enzyme residues (Figure 4B,C). Finally, the phenyl tail is oriented toward the hydrophobic side of the active site^13^ establishing many hydrophobic interactions with residues delimiting this region (Figures 3 and 4A and Table S2) and interacting with residue Phe131 as hypothesized in the design. The comparable number and nature of the interactions established by the inhibitor in the two active sites might explain the similarity of the inhibition constants against the two isoforms (Table S2). On the basis of the orientations of the phosphonate oxygens depicted in Figures 3 and 4, it can be supposed that the substituents included in the P-based linker, both in the (*S*)- and (*R*)-enantiomers, are directed toward and binded the medium and target portion of the CA active site (Figure 2B).

### Drug Stability Study

The selected subset of compounds (**6**, **7**, **10**, **18**, **20**, and **22**) was promoted to *in vivo* evaluation of their neuropathic pain-relieving action. As the drug administration would have been performed per os, a drug stability study was carried out to verify the endurance of the compounds to different media such as phosphate buffer (PBS), a pH 2 aqueous solution (i.e., HCl_(aq)_ 10 mM) to mimic the stomach environment of the animal, and human plasma (H-Pl) to check the efficiency of hydrolytic enzymes against such phosphonic ester/amide derivatives. The study was performed by coupling liquid chromatography to a mass spectrometer working in the tandem mass spectrometry mode (LC–MS/MS).

The collected data indicate that the tested derivatives exhibited stability to all the tested matrices/solutions (Figures S1–S6, Supporting Information), with the exception of derivative **18** that showed a quick degradation (*t*_1/2_ of approximately 2 min) in the pH 2 solution (Figure S4, Supporting Information).

Absorption, distribution, metabolism, excretion, and toxicity (ADMET) properties for **6**, **7**, **10**, **20**, and **22** were also predicted by the online server PreADMET^37^ and are reported in Table S4, Supporting Information.

### *In Vivo* Neuropathic Pain-Relieving Action

**Figure 5:**
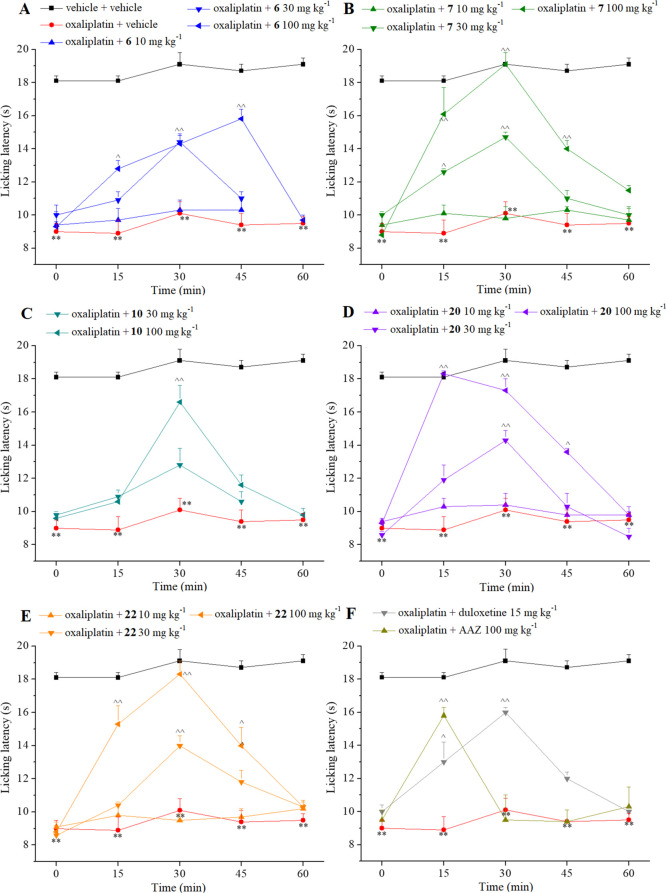
(A–F) Effect of acute administration of carbonic anhydrase inhibitors **6**, **7**, **10**, **20**, **22**, **AAZ**, and duloxetine on oxaliplatin-induced neuropathic pain in the mouse cold plate test. Oxaliplatin (2.4 mg kg^–1^) was i.p. administered for 5 consecutive days every week for 2 weeks. Compounds were dissolved in 1% CMC and per os administered acutely when neuropathy was well established (day 15). Each value represents the means ± SEM of 12 mice collected in two different experimental sets. Statistical analysis is one-way ANOVA followed by Bonferroni’s post-hoc comparison. **P < 0.s01 vs vehicle + vehicle-treated animal; ^^^*P* < 0.05 and ^^^^*P* < 0.01 vs oxaliplatin + vehicle-treated animals.

The selected compounds except **18**, shown to be unstable in acidic media, were evaluated *in vivo* for their properties in relieving neuropathic pain induced by the anticancer drug oxaliplatin that is characterized by a relevant neurotoxicity in a high percentage of patients.^38^ This platinum derivative has become a valid option as adjuvant therapy in several types of cancer, but as a common side effect, it provokes a painful neuropathy associated with characteristic nervous system alterations that reduce the patients’ quality of life.^39^ The pain-relieving effect obtained with the CAIs was compared to that achieved with the reference drugs **AAZ** and duloxetine, and the results are shown in Figure 5. Ten oxaliplatin injections decreased the licking latency to 9.0 ± 0.5 s in comparison to 18.1 ± 0.3 s of the control group (vehicle + vehicle-treated animals). Compound **6**, acutely per os administered in a range doses from 10 to 100 mg kg^–1^, evoked a dose-dependent antineuropathic effect that lasted up to 45 min after treatment with a peak of efficacy at the same time point (Figure 5A). A better antineuropathic profile was obtained with compound **7** (Figure 5B), which also showed a dose-dependent antinociceptive effect. The higher dose (100 mg kg^–1^) was able to totally counteract oxaliplatin hypersensitivity 30 min after treatment with a pain-relieving effect that continued up to 45 min. The 30 mg kg^–1^ dose also statistically amplified the pain threshold at 15 and 30 min. Likewise, notable results were attained by administration of derivatives **20** and **22**, as depicted in Figure 5D,E. Compound **10** evoked a maximum effect 30 min after administration and uniquely at the dose of 100 mg kg^–1^ (Figure 5C). The reference CAI **AAZ** was also moderately active at the dose of 100 mg kg^–1^ but solely at 15 min after injection (a licking latency of 15.8 ± 0.7 s) (Figure 5F).

The weak activity of compound **10**, although a strong dual CA II and VII inhibition, can be related to its low water solubility with respect to the other derivatives due to the presence of a double benzenesulfonamide scaffold. Barring **10**, derivatives **7** and **22**, which totally reverted allodynia *in vivo* after 30 min post-administration, are the most potent CA II inhibitors among the four remaining compounds. The slightly less effective pain-relieving action of **22** with respect to **7**, although 2-fold more potent as the CA VII inhibitor, can be related to its minor water solubility (as predicted *in silico*, Table S4). In contrast, compound **6**, 4-fold less potent as the CA II inhibitor than **7** and **22**, but as active as **22** as the CA VII inhibitor, showed a significantly inferior *in vivo* efficacy. As a result, excluding other participating factors, a greater implication of CA II than CA VII could be speculated. Surprising is the case of methylpiperazine derivative **20**, that is, it is the worst CA II and VII inhibitors among the five compounds but exhibited the quickest *in vivo* action, reverting the pain sensation after only 15 min. This is likely due to its greater water solubility, which favors its dissolution after per os administration.

The pain-relieving efficacy of the most active derivatives **7**, **20**, and **22** was comparable (at 30 mg kg^–1^) or even greater (at 100 mg kg^–1^) than that induced by duloxetine (when dosed at 15 mg kg^–1^, a neuropathic pain-relieving dose in animals^40^ (Figure 5F), a standard drug clinically used for the management of chemotherapy-induced neuropathy.^41^ It should be thus stressed that further comparison between the doses of these phosphon(amid)ates and duloxetine cannot be done as such compounds did not share the same mechanism of action.

### Chiral Resolution and Assignment of Absolute Configuration of Compound **7**

**Figure 6:**
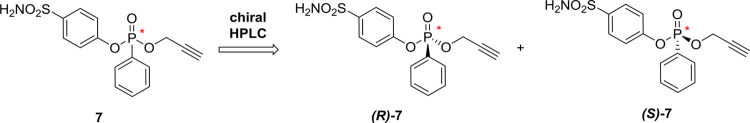
Chiral resolution of compound **7** by chiral HPLC.

**Figure 7:**
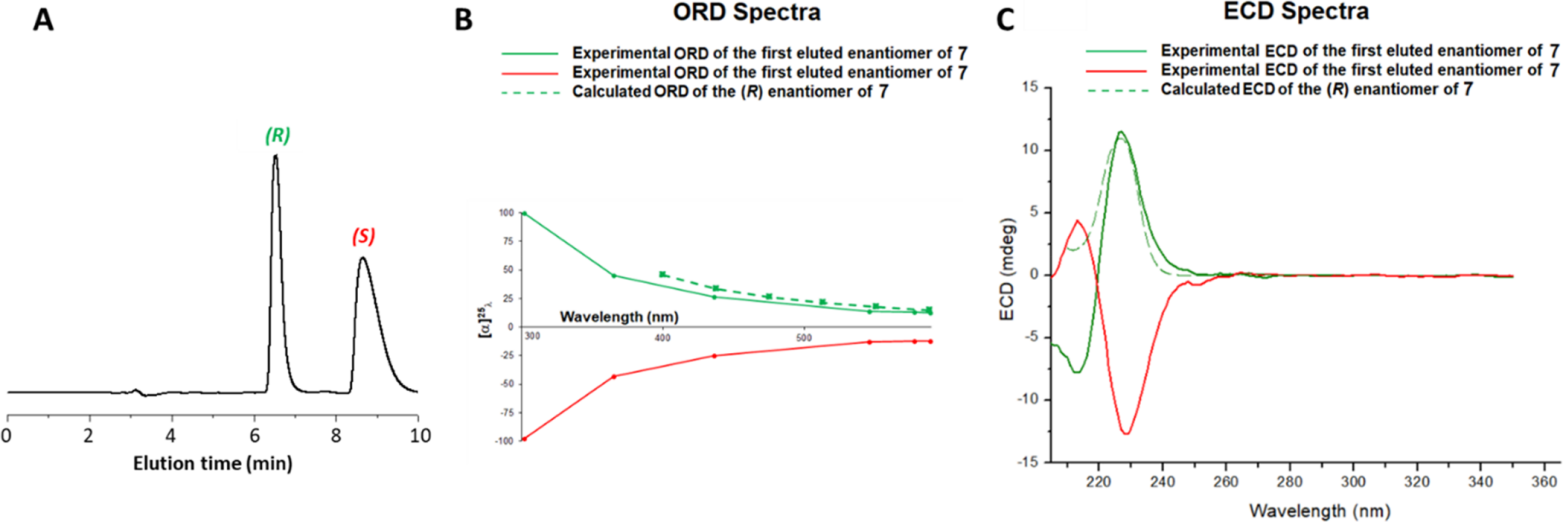
(A) Typical chromatogram illustrating the enantioseparation of 1 mg of **7** on the 250 mm × 10 mm I.D. CHIRALPAK IA column. Mobile phase: *n*-hexane/EtOH/MeOH/TFA 60:35:5:0.1 (v/v/v/v); flow rate: 4.5 mL min^–1^; temperature: 15 °C; detector: UV at 260 nm. (B) ORD and (C) ECD spectra of the enantiomers of **7** recorded in ethanol at 25 °C (solid lines) and calculated (dashed lines). Green and red lines refer to the first and second eluted enantiomers, respectively.

The racemic mixture of the most *in vivo* effective compound, namely, compound **7**, was subjected to enantioseparation, accomplished by HPLC on the immobilized-type CHIRALPAK IA chiral stationary phase under the normal-phase mode (Figure 6). Both enantiomers could be collected in multimilligram quantities, by performing repetitive injections of 1 mg of racemic sample on a 250 mm × 10 mm I.D. CHIRALPAK IA column. As shown in Figure 7A, the semipreparative HPLC resolution was achieved in nonoverlapping band conditions. The analytical control of the collected fractions gave an enantiomeric excess of both enantiomers higher than 98%.

To determine the absolute configuration and then the enantiomer elution order on the chiral chromatographic support, the chiroptical properties (i.e., CD and ORD spectra) of the enantiomers isolated by HPLC were measured and compared with the calculated ones (Figure 7B,C).

A detailed description of the adopted procedure is provided in the Experimental Section.

### Carbonic Anhydrase Inhibition of Enantiomers of Compound **7**

(*R*)*-* and (*S*)*-*enantiomers of compound **7** were evaluated for their inhibition against the cytosolic CAs I, II, and VII, the mitochondrial CAs VA and VB, and the membrane-related CAs IV, IX, and XII. The following considerations can be made from the data reported in Table 2.

**Table 2:** Inhibition Data of Human CA Isoforms I, II, IV, VA, VB, VII, IX, and XII with Compound **7** as the Racemic Mixture and its (*R*)- and (*S*)-Enantiomers Using **AAZ** as the Standard^a^

	*K*_I_ (nM)							
cmp	CA I	CA II	CA IV	CA VA	CA VB	CA VII	CA IX	CA XII
**7**	415.3 ± 29.0	3.6 ± 0.2	1322 ± 124	82.1 ± 5.7	38.4 ± 2.5	12.2 ± 0.8	22.6 ± 1.6	26.7 ± 1.8
**(*R*)-7**	365.4 ± 23.4	56.4 ± 3.8	1984 ± 153	66.5 ± 4.6	66.1 ± 3.5	71.3 ± 4.5	47.2 ± 2.8	49.2 ± 3.2
**(*S*)-7**	564.1 ± 42.7	0.78 ± 0.06	746.5 ± 38.4	108.3 ± 8.6	19.2 ± 1.5	1.1 ± 0.1	10.5 ± 1.4	17.3 ± 1.3

a Inhibition data are expressed as means ± SEM of three different assays.

Enantiomer **(*S*)-7** resulted to be the eutomer within the racemic mixture against most screened CA isoforms, which are CAs II, IV, VB, VII, IX, and XII. Unique exceptions are CAs I and VA, against which **(*R*)-7** showed *K*_I_ values of 365.4 and 66.5 nM, respectively, arising as the eutomer. Astonishingly, the CA inhibition profile of **(*S*)-7** was even improved when compared to **7** in terms of potency and selectivity for the target CAs over off-target ones. Indeed, CA II was inhibited at a subnanomolar value of 0.78 nM by **(*S*)-7**, whereas the *K*_I_ against CA VII reached the single-digit value of 1.1 nM. Moreover, the *K*_I_ of **(*S*)-7** halved against CA VB and CA IX with respect to **7**, while an inferior difference was measured between *K*_I_ values of **(*S*)-7** and **7** as the racemic mixture against CA XII (17.3 and 26.7 nM, respectively).

### *In Vivo* Neuropathic Pain-Relieving Action with **(*R*)-7** and **(*S*)-7**

**Figure 8:**
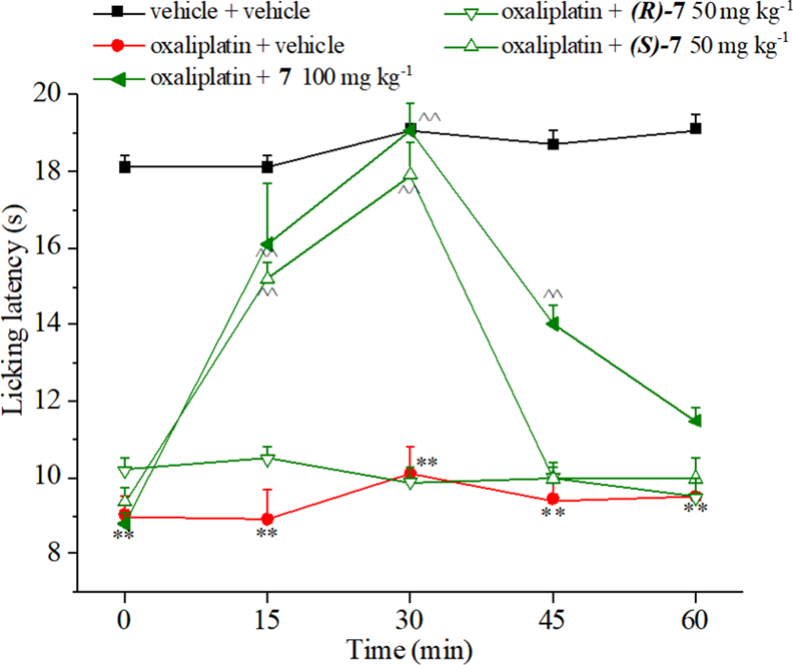
Effect of acute administration of eutomers **(*R*)-7** and **(*S****)-***7**, in comparison with **7** on oxaliplatin-induced neuropathic pain in the mouse cold plate test. Oxaliplatin (2.4 mg kg^–1^) was i.p. administered for 5 consecutive days every week for 2 weeks. Compounds were dissolved in 1% CMC and per os administered acutely when neuropathy was well established (day 15). Each value represents the means ± SEM of 12 mice collected in two different experimental sets. Statistical analysis is one-way ANOVA followed by Bonferroni’s post-hoc comparison. **P < 0.01 vs vehicle + vehicle-treated animals; ^^^*P* < 0.05 and ^^^^*P* < 0.01 vs oxaliplatin + vehicle-treated animals.

The *in vivo* action of the two enantiomers of compound **7** was thus assessed in the same mouse model of neuropathic pain when hyperalgesia was well established (Figure 8). Searching for a dose reverting the allodynia and expecting one of them having a greater efficacy than **7** as the racemate, a dose of 50 mg kg^–1^ was used. According to *in vitro* outcomes, the study identified **(*S*)-7**, the eutomer, since it counteracted oxaliplatin-induced hyperalgesia comparably to **7** but notably by the half dose. Surprisingly, 50 mg kg^–1^**(*R*)-7** was totally ineffective. This might indicate that a CA II and/or VII inhibition of a certain intensity (*K*_I_ values of **(*R*)-7** against CAs II and VII dropped by 15 and 6 times, respectively, with respect to **7**) is necessary to produce an action, which relieves neuropathic pain.

## Conclusions

NP is induced by ailments or lesion existing in the CNS, which produces the sensation of pain in the brain. New promising drug classes are necessary because of difficulties met in treating NP in a vast majority of patients. The human CA isoforms II and VII are implicated in neuronal excitation, seizures, and NP. Therefore, their selective inhibition over off-target CAs is expected to produce an anti-NP action devoid of side effects, which arise by promiscuous CA inhibition. In this study, a drug design strategy was planned to produce selective CA II and VII inhibitors. Benzenesulfonamide derivatives with a phosphorus-based linker were adopted for the first time with the aim of pursuing the tail approach for the exploration of the middle portion of the CA active site cavity. A subset of potent and selective CA II/VII inhibitors was identified among the synthesized phenyl(thio)phosphon(amid)ates **3**–**22**. It is fair to stress that these compounds showed selectivity for CAs II and VII above one order of magnitude solely over CAs I, IV, and VA. In contrast, the selectivity index was lower for CAs II and VII over CAs VB, IX, and XII, which are, however, minor off-target isoforms with respect to the ubiquitous CA I.

The efficacy of the adopted drug design strategy was confirmed at the molecular level by X-ray crystallographic studies depicting the binding mode of phosphonic acid **3** to both CAs II and VII. The most promising derivatives for potent and preferred inhibition against the target CAs II and VII over all other isoforms, after evaluation for their stability in acidic media, were tested in a mouse model of oxaliplatin-induced neuropathy. The most potent compound (**7**) racemic mixture was also subjected to semipreparative HPLC resolution, and the identification of the eutomer for CA II and VII inhibition **(*S*)-7** even allowed to halve the dose totally reverting allodynia in mice from 100 to 50 mg kg^–1^.

These results testify the efficacy of the adopted drug design strategy toward CA II/VII inhibitors for the treatment of NP and overall support the potentiality of the (thio)phosphon(amid)ate linker for yielding potent and selective CA inhibitors for therapeutic applications.

## Experimental Section

### Chemistry

Anhydrous solvents and all reagents were purchased from Sigma-Aldrich, Fluorochem, and TCI. All reactions involving air- or moisture-sensitive compounds were performed under a nitrogen atmosphere using dried glassware and syringe techniques to transfer solutions. Nuclear magnetic resonance (^1^H NMR, ^13^C NMR, and ^31^P NMR) spectra were recorded using a Bruker Avance III 400 MHz spectrometer in DMSO-*d*_6_. Chemical shifts are reported in parts per million (ppm), and the coupling constants (*J*) are expressed in hertz (Hz). Splitting patterns are designated as follows: s, singlet; d, doublet; sept, septet; t, triplet; q, quadruplet; m, multiplet; bs, broad singlet; and dd, double of doublets. The assignment of exchangeable protons (O*H* and N*H*) was confirmed by the addition of D_2_O. Analytical thin-layer chromatography (TLC) was carried out on Sigma-Aldrich silica gel F-254 plates. Flash chromatography purifications were performed on Sigma-Aldrich silica gel 60 (230–400 mesh ASTM) as the stationary phase, and ethyl acetate/*n*-hexane or MeOH/DCM was used as eluents. Melting points (mp) were measured in open capillary tubes with a Gallenkamp MPD350.BM3.5 apparatus and are uncorrected.

Compounds **3**–**22** were ≥95% pure. The purity of the final compounds was determined by an Agilent 1200 liquid chromatography system composed by an autosampler, binary pumps, a column oven, and a diode array detector (LC-DAD) operating in the UV range (210–400 nm). The operating conditions are reported in the Supporting Information. The following chromatographic parameters were employed to check the purity of the compounds: (i) column, Luna PFP length = 50 mm, internal diameter = 2 mm; particle size = 3 μm purchased from Phenomenex (Bologna, Italy); (ii) the eluents used were 10 mM formic acid and 5 mM ammonium formate in water solution (solvent A) and 10 mM ammonium formate and 5 mM formic acid in methanol (solvent B); (iii) the flow rate and injection volume were 0.25 mL min^–1^ and 5 μL, respectively. The elution gradient is shown in Table S3, Supporting Information. The HPLC chromatograms are depicted in Figures S7–S27, Supporting Information.

The high-resolution mass spectrometry (HRMS) analysis was performed with a Thermo Finnigan LTQ Orbitrap mass spectrometer equipped with an electrospray ionization source (ESI). The analysis was carried out by introducing, via a syringe pump at 10 μL min^–1^, the sample solution (1.0 μg mL^–1^ in mQ water/acetonitrile 50:50) and acquired the signal of the positive ions. These experimental conditions allow the monitoring of protonated molecules of the studied compounds ([M + H]^+^ species) that were measured with a proper dwell time to achieve 60,000 units of resolution at full width at half-maximum (FWHM). Elemental compositions of compounds were calculated on the basis of their measured accurate masses, accepting only results with an attribution error of less than 2.5 ppm and a noninteger RDB (double bond/ring equivalents) value, to consider only the protonated species.^42^

### General Synthetic Procedure of Compounds (**3**–**22**)

Phenylphosphonic dichloride **2** (1.2 equiv) was quickly added to a solution of 4-hydroxybenzenesulfonamide **1** (0.3 g, 1.0 equiv) and trimethylamine (2.2 equiv) in dry THF (10 mL) at −5 °C under a nitrogen atmosphere, and the resulting suspension was stirred for 0.5 h at 0 °C. The proper reactant was added to the mixture and that was stirred at r.t. for 1 h. The reaction mixture was concentrated *in vacuo*, and the obtained residue was purified by silica gel column chromatography eluting with MeOH/DCM to afford the title compounds **3**–**23** as powders.

#### 4-Sulfamoylphenyl Hydrogen Phenylphosphonate (**3**)

Compound **3** was obtained according to the general procedure earlier reported using H_2_O (5.0 equiv), phenylphosphonic dichloride **2** (1.2 equiv), 4-hydroxybenzenesulfonamide **1** (0.3 g, 1.0 equiv), and trimethylamine (2.2 equiv) in dry THF (10 mL). The reaction mixture was concentrated *in vacuo*, and the obtained residue was purified by silica gel column chromatography eluting with 4% MeOH in DCM to afford the title compound as a white solid. 55% yield; mp 183–184 °C; silica gel TLC *R*_f_, 0.11 (MeOH/CH_2_Cl_2_ 15% v/v); δ_H_ (400 MHz, DMSO-*d*_6_): 7.32 (m, 4H, partial exchange with D_2_O, Ar-*H* + SO_2_N*H*_2_), 7.55 (m, 2H, Ar-*H*), 7.63 (m, 1H, Ar-*H*), 7.87 (m, 4H, Ar-*H*); δ_C_ (100 MHz, DMSO-*d*_6_): 121.56 (d, *J*^3^_CP_ = 4.9), 128.50, 129.48 (d, *J*^2^_CP_ = 14.7), 131.05 (d, *J*^1^_CP_ = 185.8), 132.21 (d, *J*^3^_CP_ = 9.9), 133.16 (d, *J*^4^_CP_ = 3.2), 140.77, 154.27 (d, *J*^2^_CP_ = 6.6); δ_P_ (162 MHz, DMSO-*d*_6_): 12.01; ESI-HRMS (*m/z*): [M + H]^+^ calcd for C_12_H_13_NO_5_PS, 314.0252; found, 314.0247.

#### Methyl (4-Sulfamoylphenyl) Phenylphosphonate (**4**)

Compound **4** was obtained according to the general procedure earlier reported using MeOH (5.0 equiv), phenylphosphonic dichloride **2** (1.2 equiv), 4-hydroxybenzenesulfonamide **1** (0.3 g, 1.0 equiv), and trimethylamine (2.2 equiv) in dry THF (10 mL). The reaction mixture was concentrated *in vacuo*, and the obtained residue was purified by silica gel column chromatography eluting with 4% MeOH in DCM to afford the title compound as a white waxy solid. 28% yield; mp 92–93 °C; silica gel TLC *R*_f_, 0.31 (MeOH/CH_2_Cl_2_ 5% v/v); δ_H_ (400 MHz, DMSO-*d*_6_): 3.87 (d, *J* = 11.4, 3H, OC*H*_3_), 7.38 (s, 2H, exchange with D_2_O, SO_2_N*H*_2_), 7.41 (m, 2H, Ar-*H*), 7.64 (m, 2H, Ar-*H*), 7.75 (m, 1H, Ar-*H*), 7.81 (m, 4H, Ar-*H*); δ_C_ (100 MHz, DMSO-*d*_6_): 54.29 (d, *J*^2^_CP_ = 6.0), 121.64 (d, *J*^3^_CP_ = 4.4), 126.95 (d, *J*^1^_CP_ = 187.8), 128.74, 129.95 (d, *J*^2^_CP_ = 15.2), 132.63 (d, *J*^3^_CP_ = 10.4), 134.39 (d, *J*^4^_CP_ = 3.1), 141.61, 153.29 (d, *J*^2^_CP_ = 6.6); δ_P_ (162 MHz, DMSO-*d*_6_): 16.73; ESI-HRMS (*m/z*): [M + H]^+^ calcd for C_13_H_15_NO_5_PS, 328.0408; found, 328.0402.

#### Ethyl (4-Sulfamoylphenyl) Phenylphosphonate (**5**)

Compound **5** was obtained according to the general procedure earlier reported using EtOH (5.0 equiv), phenylphosphonic dichloride **2** (1.2 equiv), 4-hydroxybenzenesulfonamide **1** (0.3 g, 1.0 equiv), and trimethylamine (2.2 equiv) in dry THF (10 mL). The reaction mixture was concentrated *in vacuo*, and the obtained residue was purified by silica gel column chromatography eluting with 4% MeOH in DCM to afford the title compound as a white waxy solid. 33% yield; mp 98–99 °C; silica gel TLC *R*_f_, 0.52 (MeOH/CH_2_Cl_2_ 10% v/v); δ_H_ (400 MHz, DMSO-*d*_6_): 1.32 (t, *J* = 7.0, 3H, CH_2_C*H*_3_), 4.25 (m, 2H, OC*H*_2_), 7.38 (s, 2H, exchange with D_2_O, SO_2_N*H*_2_), 7.40 (m, 2H, Ar-*H*), 7.62 (m, 2H, Ar-*H*), 7.73 (m, 1H, Ar-*H*), 7.86 (m, 4H, Ar-*H*); δ_C_ (100 MHz, DMSO-*d*_6_): 17.05 (d, *J*^3^_CP_ = 6.1), 64.01 (d, *J*^2^_CP_ = 5.9), 121.71 (d, *J*^3^_CP_ = 4.8), 127.65 (d, *J*^1^_CP_ = 188.6), 128.77, 129.97 (d, *J*^2^_CP_ = 15.3), 132.59 (d, *J*^3^_CP_ = 10.3), 134.33 (d, *J*^4^_CP_ = 3.2), 141.55, 153.40 (d, *J*^2^_CP_ = 6.7); δ_P_ (162 MHz, DMSO-*d*_6_): 15.36; ESI-HRMS (*m/z*): [M + H]^+^ calcd for C_14_H_17_NO_5_PS, 342.0565; found, 342.0560.

#### Isopropyl (4-Sulfamoylphenyl) Phenylphosphonate (**6**)

Compound **6** was obtained according to the general procedure earlier reported using ^i^PrOH (5.0 equiv), phenylphosphonic dichloride **2** (1.2 equiv), 4-hydroxybenzenesulfonamide **1** (0.3 g, 1.0 equiv), and trimethylamine (2.2 equiv) in dry THF (10 mL). The reaction mixture was concentrated *in vacuo*, and the obtained residue was purified by silica gel column chromatography eluting with 4% MeOH in DCM to afford the title compound as a white solid. 44% yield; mp 106–107 °C; silica gel TLC *R*_f_, 0.45 (MeOH/CH_2_Cl_2_ 10% v/v); δ_H_ (400 MHz, DMSO-*d*_6_): 1.33 (dd, *J* = 6.2, 22.4, 6H, CH(C*H*_3_)_2_), 4.83 (m, 1H, OC*H*), 7.37 (s, 2H, exchange with D_2_O, SO_2_N*H*_2_, overlap with a signal at 7.39), 7.39 (m, 2H, Ar-*H*), 7.62 (m, 2H, Ar-*H*), 7.72 (m, 1H, Ar-*H*), 7.86 (m, 4H, Ar-*H*); δ_C_ (100 MHz, DMSO-*d*_6_): 24.43 (d, *J*^3^_CP_ = 7.3), 73.07 (d, *J*^2^_CP_ = 6.0), 121.71 (d, *J*^3^_CP_ = 4.8), 128.36 (d, *J*^1^_CP_ = 188.2), 128.64, 129.81 (d, *J*^2^_CP_ = 15.3), 132.43 (d, *J*^3^_CP_ = 10.3), 134.08 (d, *J*^4^_CP_ = 3.0), 141.43, 153.42 (d, *J*^2^_CP_ = 6.7); δ_P_ (162 MHz, DMSO-*d*_6_): 14.31; ESI-HRMS (*m/z*): [M + H]^+^ calcd for C_15_H_19_NO_5_PS, 356.0721; found, 356.0716.

#### Propargyl (4-Sulfamoylphenyl) Phenylphosphonate (**7**)

Compound **7** was obtained according to the general procedure earlier reported using propargyl alcohol (5.0 equiv), phenylphosphonic dichloride **2** (1.2 equiv), 4-hydroxybenzenesulfonamide **1** (0.3 g, 1.0 equiv), and trimethylamine (2.2 equiv) in dry THF (10 mL). The reaction mixture was concentrated *in vacuo*, and the obtained residue was purified by silica gel column chromatography eluting with 4% MeOH in DCM to afford the title compound as a white solid. 51% yield; mp 165–166 °C; silica gel TLC *R*_f_, 0.28 (MeOH/CH_2_Cl_2_ 5% v/v); δ_H_ (400 MHz, DMSO-*d*_6_): 3.74 (t, *J* = 2.4, 1H, CH_2_C*H*), 4.94 (m, 2H, OC*H*_2_), 7.39 (s, 2H, exchange with D_2_O, SO_2_N*H*_2_), 7.42 (m, 2H, Ar-*H*), 7.64 (m, 2H, Ar-*H*), 7.75 (m, 1H, Ar-*H*), 7.89 (m, 4H, Ar-*H*); δ_C_ (100 MHz, DMSO-*d*_6_): 55.30 (d, *J*^3^_CP_ = 6.0), 78.89 (d, *J*^2^_CP_ = 6.6), 79.99, 121.65 (d, *J*^3^_CP_ = 4.4), 127.06 (d, *J*^1^_CP_ = 190.03), 128.68, 129.87 (d, *J*^2^_CP_ = 15.5), 132.53 (d, *J*^3^_CP_ = 10.7), 134.46 (d, *J*^4^_CP_ = 3.2), 141.71, 153.06 (d, *J*^2^_CP_ = 7.3); δ_P_ (162 MHz, DMSO-*d*_6_): 16.32; ESI-HRMS (*m/z*): [M + H]^+^ calcd for C_15_H_15_NO_5_PS, 352.0408; found, 352.0402.

#### 2-Methoxyethyl (4-Sulfamoylphenyl) Phenylphosphonate (**8**)

Compound **8** was obtained according to the general procedure earlier reported using 2-methoxyethanol (5.0 equiv), phenylphosphonic dichloride **2** (1.2 equiv), 4-hydroxybenzenesulfonamide **1** (0.3 g, 1.0 equiv), and trimethylamine (2.2 equiv) in dry THF (10 mL). The reaction mixture was concentrated *in vacuo*, and the obtained residue was purified by silica gel column chromatography eluting with 4% MeOH in DCM to afford the title compound as a white solid. 61% yield; mp 102–103 °C; silica gel TLC *R*_f_, 0.47 (MeOH/CH_2_Cl_2_ 20% v/v); δ_H_ (400 MHz, DMSO-*d*_6_): 3.25 (s, 3H, OC*H*_3_), 3.58 (m, 2H, C*H*_2_), 4.29 (m, 2H, C*H*_2_), 7.37 (s, 2H, exchange with D_2_O, SO_2_N*H*_2_), 7.41 (m, 2H, Ar-*H*), 7.63 (m, 2H, Ar-*H*), 7.73 (m, 1H, Ar-*H*), 7.87 (m, 4H, Ar-*H*); δ_C_ (100 MHz, DMSO-*d*_6_): 58.82, 66.62 (d, *J*^3^_CP_ = 5.9), 71.56 (d, *J*^2^_CP_ = 6.5),121.60 (d, *J*^3^_CP_ = 4.5), 127.55 (d, *J*^1^_CP_ = 190.1), 128.64, 129.80 (d, *J*^2^_CP_ = 15.2), 132.49 (d, *J*^3^_CP_ = 10.3), 134.20 (d, *J*^4^_CP_ = 3.0), 141.54, 153.29 (d, *J*^2^_CP_ = 7.2); δ_P_ (162 MHz, DMSO-*d*_6_): 15.67; ESI-HRMS (*m/z*): [M + H]^+^ calcd for C_15_H_16_NO_6_PS, 369.0436; found, 369.0431.

#### Phenyl (4-Sulfamoylphenyl) Phenylphosphonate (**9**)

Compound **9** was obtained according to the general procedure earlier reported using phenol (2.0 equiv), phenylphosphonic dichloride **2** (1.2 equiv), 4-hydroxybenzenesulfonamide **1** (0.3 g, 1.0 equiv), and trimethylamine (2.2 equiv) in dry THF (10 mL). The reaction mixture was concentrated *in vacuo*, and the obtained residue was purified by silica gel column chromatography eluting with 4% MeOH in DCM to afford the title compound as a white solid. 53% yield; mp 111–112 °C; silica gel TLC *R*_f_, 0.51 (MeOH/CH_2_Cl_2_ 10% v/v); δ_H_ (400 MHz, DMSO-*d*_6_): 7.25 (m, 2H, Ar-*H*), 7.27 (m, 3H, Ar-*H*), 7.41 (s, 2H, exchange with D_2_O, SO_2_N*H*_2_), 7.45 (m, 2H, Ar-*H*), 7.65 (m, 2H, Ar-*H*), 7.78 (m, 1H, Ar-*H*), 7.88 (m, 2H, Ar-*H*), 8.00 (m, 2H, Ar-*H*); δ_C_ (100 MHz, DMSO-*d*_6_): 121.64 (d, *J*^3^_CP_ = 4.4), 121.72 (d, *J*^3^_CP_ = 4.6), 126.41, 126.43 (d, *J*^1^_CP_ = 190.0), 128.86, 130.07 (d, *J*^2^_CP_ = 15.5), 130.97, 132.98 (d, *J*^3^_CP_ = 10.8), 134.87 (d, *J*^4^_CP_ = 2.9), 141.95, 150.60 (d, *J*^2^_CP_ = 7.4), 152.96 (d, *J*^2^_CP_ = 7.3); δ_P_ (162 MHz, DMSO-*d*_6_): 12.25; ESI-HRMS (*m/z*): [M + H]^+^ calcd for C_18_H_17_NO_5_PS, 390.0565; found, 390.0563.

#### Bis(4-sulfamoylphenyl) Phenylphosphonate (**10**)

Compound **10** was obtained according to the general procedure earlier reported using phenylphosphonic dichloride **2** (0.5 equiv), 4-hydroxybenzenesulfonamide **1** (0.5 g, 1.0 equiv). and trimethylamine (2.2 equiv) in dry THF (10 mL). The reaction mixture was concentrated *in vacuo*, and the obtained residue was purified by silica gel column chromatography eluting with 10% MeOH in DCM to afford the title compound as a yellow solid. 38% yield; mp 95–96 °C; silica gel TLC *R*_f_, 0.26 (MeOH/CH_2_Cl_2_ 10% v/v); δ_H_ (400 MHz, DMSO-*d*_6_): 7.41 (s, 4H, exchange with D_2_O, 2 × SO_2_N*H*_2_), 7.47 (m, 4H, Ar-*H*), 7.68 (m, 2H, Ar-*H*), 7.80 (m, 1H, Ar-*H*), 7.88 (m, 4H, Ar-*H*), 8.03 (m, 2H, Ar-*H*); δ_C_ (100 MHz, DMSO-*d*_6_): 121.79 (d, *J*^3^_CP_ = 4.5), 125.86 (d, *J*^1^_CP_ = 190.10), 128.93, 130.21 (d, *J*^2^_CP_ = 15.9), 133.06 (d, *J*^3^_CP_ = 11.0), 135.15, 142.14, 152.76 (d, *J*^2^_CP_ = 6.9); δ_P_ (162 MHz, DMSO-*d*_6_): 12.49; ESI-HRMS (*m/z*): [M + H]^+^ calcd for C_18_H_18_N_2_O_7_PS_2_, 469.0293; found, 469.0296.

#### *S*-Ethyl *O*-(4-Sulfamoylphenyl) Phenylphosphonothioate (**11**)

Compound **11** was obtained according to the general procedure earlier reported using mercaptoethanol (2.0 equiv), phenylphosphonic dichloride **2** (1.2 equiv), 4-hydroxybenzenesulfonamide **1** (0.3 g, 1.0 equiv), and trimethylamine (2.2 equiv) in dry THF (10 mL). The reaction mixture was concentrated *in vacuo*, and the obtained residue was purified by silica gel column chromatography eluting with 4% MeOH in DCM to afford the title compound as a white solid. 36% yield; mp 109–110 °C; silica gel TLC *R*_f_, 0.39 (MeOH/CH_2_Cl_2_ 10% v/v); δ_H_ (400 MHz, DMSO-*d*_6_): 1.16 (t, *J* = 7.4, 3H, CH_2_C*H*_3_), 2.83 (m, 2H, SC*H*_2_), 7.43 (s, 2H, exchange with D_2_O, SO_2_N*H*_2_), 7.54 (m, 2H, Ar-*H*), 7.68 (m, 2H, Ar-*H*), 7.77 (m, 1H, Ar-*H*), 7.91 (m, 2H, Ar-*H*), 8.00 (m, 2H, Ar-*H*); δ_C_ (100 MHz, DMSO-*d*_6_): 16.93 (d, *J*^2^_CP_ = 4.9), 25.75 (d, *J*^3^_CP_ = 3.0), 122.25 (d, *J*^3^_CP_ = 4.7), 128.79, 130.6 (d, *J*^2^_CP_ = 14.7), 131.79 (d, *J*^3^_CP_ = 11.5), 132.33 (d, *J*^1^_CP_ = 147.1), 134.40 (d, *J*^4^_CP_ = 3.3), 141.91, 153.24 (d, *J*^2^_CP_ = 9.1); δ_P_ (162 MHz, DMSO-*d*_6_): 43.52; ESI-HRMS (*m/z*): [M + H]^+^ calcd for C_14_H_15_NO_4_PS_2_, 356.0180; found, 356.0176.

#### *S*-Dodecyl *O*-(4-Sulfamoylphenyl) Phenylphosphonothioate (**12**)

Compound **12** was obtained according to the general procedure earlier reported using 1-dodecanethiol (2.0 equiv), phenylphosphonic dichloride **2** (1.2 equiv), 4-hydroxybenzenesulfonamide **1** (0.3 g, 1.0 equiv), and trimethylamine (2.2 equiv) in dry THF (10 mL). The reaction mixture was concentrated *in vacuo*, and the obtained residue was purified by silica gel column chromatography eluting with 4% MeOH in DCM to afford the title compound as a white waxy solid. 29% yield; mp 62–63 °C; silica gel TLC *R*_f_, 0.45 (MeOH/CH_2_Cl_2_ 10% v/v); 0.89 (t, *J* = 7.0, 3H, CH_2_C*H*_3_), 1.21 (m, 18H, 9 × C*H*_2_), 1.46 (m, 2H, C*H*_2_), 2.81 (m, 2H, C*H*_2_), 7.42 (s, 2H, exchange with D_2_O, SO_2_N*H*_2_), 7.53 (m, 2H, Ar-*H*), 7.67 (m, 2H, Ar-*H*), 7.76 (m, 1H, Ar-*H*), 7.90 (m, 2H, Ar-*H*), 7.99 (m, 2H, Ar-*H*); δ_C_ (100 MHz, DMSO-*d*_6_): 14.84, 22.98, 28.45, 29.08, 29.57, 29.61, 29.75, 29.85, 29.86, 30.76 (d, *J*^2^_CP_ = 4.5), 31.09 (d, *J*^3^_CP_ = 2.9), 32.18, 122.19 (d, *J*^3^_CP_ = 5.0), 128.76, 130.0 (d, *J*^2^_CP_ = 14.8), 131.89 (d, *J*^3^_CP_ = 11.2), 132.34 (d, *J*^1^_CP_ = 147.1), 134.36 (d, *J*^4^_CP_ = 3.3), 141.96, 153.23 (d, *J*^2^_CP_ = 9.1); δ_P_ (162 MHz, DMSO-*d*_6_): 43.65; ESI-HRMS (*m/z*): [M + H]^+^ calcd for C_24_H_37_NO_4_PS_2_, 498.1901; found, 498.1896.

#### *S*-Benzyl *O*-(4-Sulfamoylphenyl) Phenylphosphonothioate (**13**)

Compound **13** was obtained according to the general procedure earlier reported using benzyl mercaptan (2.0 equiv), phenylphosphonic dichloride **2** (1.2 equiv), 4-hydroxybenzenesulfonamide **1** (0.3 g, 1.0 equiv), and trimethylamine (2.2 equiv) in dry THF (10 mL). The reaction mixture was concentrated *in vacuo*, and the obtained residue was purified by silica gel column chromatography eluting with 4% MeOH in DCM to afford the title compound as a white solid. 57% yield; mp 201–202 °C; silica gel TLC *R*_f_, 0.52 (MeOH/CH_2_Cl_2_ 10% v/v); δ_H_ (400 MHz, DMSO-*d*_6_): 4.10 (m, 2H, SC*H*_2_), 7.19 (m, 2H, Ar-*H*), 7.26 (m, 3H, Ar-*H*), 7.43 (s, 2H, exchange with D_2_O, SO_2_N*H*_2_), 7.50 (m, 2H, Ar-*H*), 7.65 (m, 2H, Ar-*H*), 7.75 (m, 1H, Ar-*H*), 7.89 (m, 2H, Ar-*H*), 7.97 (m, 2H, Ar-*H*); δ_C_ (100 MHz, DMSO-*d*_6_): 34.83 (d, *J*^2^_CP_ = 2.9), 122.5 (d, *J*^3^_CP_ = 5.0), 128.40, 128.74, 129.41, 129.64, 129.99 (d, *J*^2^_CP_ = 14.8), 131.89 (d, *J*^3^_CP_ = 11.3), 132.04 (d, *J*^1^_CP_ = 148.5), 134.39 (d, *J*^4^_CP_ = 3.1), 137.65 (d, *J*^3^_CP_ = 5.0), 141.96, 153.23 (d, *J*^2^_CP_ = 9.1); δ_P_ (162 MHz, DMSO-*d*_6_): 42.64; ESI-HRMS (*m/z*): [M + H]^+^ calcd for C_19_H_19_NO_4_PS_2_, 420.0493; found, 420.0487.

#### *S*-Phenyl *O*-(4-Sulfamoylphenyl) Phenylphosphonothioate (**14**)

Compound **14** was obtained according to the general procedure earlier reported using thiophenol (2.0 equiv), phenylphosphonic dichloride **2** (1.2 equiv), 4-hydroxybenzenesulfonamide **1** (0.3 g, 1.0 equiv), and trimethylamine (2.2 equiv) in dry THF (10 mL). The reaction mixture was concentrated *in vacuo*, and the obtained residue was purified by silica gel column chromatography eluting with 4% MeOH in DCM to afford the title compound as a white solid. 47% yield; mp 117–118 °C; silica gel TLC *R*_f_, 0.40 (MeOH/CH_2_Cl_2_ 10% v/v); δ_H_ (400 MHz, DMSO-*d*_6_): 7.33 (m, 4H, Ar-*H*), 7.44 (m, 1H, Ar-*H*), 7.45 (s, 2H, exchange with D_2_O, SO_2_N*H*_2_, overlap with a signal at 7.44), 7.53 (m, 2H, Ar-*H*), 7.59 (m, 2H, Ar-*H*), 7.72 (m, 1H, Ar-*H*), 7.81 (m, 2H, Ar-*H*), 7.92 (m, 2H, Ar-*H*); δ_C_ (100 MHz, DMSO-*d*_6_): 121.99 (d, *J*^3^_CP_ = 5.1), 125.50 (d, *J*^3^_CP_ = 5.3), 128.88, 129.82 (d, *J*^2^_CP_ = 14.9), 130.51, 130.61, 130.71 (d, *J*^1^_CP_ = 147.8), 132.27 (d, *J*^3^_CP_ = 11.1), 134.59 (d, *J*^4^_CP_ = 3.3), 136.13 (d, *J*^2^_CP_ = 4.4), 142.03, 153.25 (d, *J*^2^_CP_ = 9.5); δ_P_ (162 MHz, DMSO-*d*_6_): 39.97; ESI-HRMS (*m/z*): [M + H]^+^ calcd for C_18_H_17_NO_4_PS_2_, 406.0336; found, 406.0329.

#### 4-Sulfamoylphenyl *P*-Phenylphosphonamidate (**15**)

Compound **15** was obtained according to the general procedure earlier reported using NH_3(aq)_ (5.0 equiv), phenylphosphonic dichloride **2** (1.2 equiv), 4-hydroxybenzenesulfonamide **1** (0.3 g, 1.0 equiv), and trimethylamine (2.2 equiv) in dry THF (10 mL). The formed precipitate was filtered off, washed with water, and recrystallized from EtOH to afford the title compound as a white solid. 51% yield; mp 227–228 °C; silica gel TLC *R*_f_, 0.21 (MeOH/CH_2_Cl_2_ 10% v/v); δ_H_ (400 MHz, DMSO-*d*_6_): 5.36 (d, *J* = 5.6, 2H, exchange with D_2_O, PON*H*_2_), 7.31 (s, 2H, exchange with D_2_O, SO_2_N*H*_2_), 7.35 (d, *J* = 8.4, 2H, Ar-*H*), 7.53 (m, 2H, Ar-*H*), 7.56 (m, 1H, Ar-*H*), 7.78 (d, *J* = 8.4, 2H, Ar-*H*), 7.84 (m, 2H, Ar-*H*); δ_C_ (100 MHz, DMSO-*d*_6_): 121.9 (d, *J*^3^_CP_ = 4.7), 128.36, 129.26 (d, *J*^2^_CP_ = 14.3), 131.73 (d, *J*^3^_CP_ = 10.3), 132.73 (d, *J*^4^_CP_ = 3.0), 133.37 (d, *J*^1^_CP_ = 171.3), 140.65, 154.31 (d, *J*^2^_CP_ = 7.5); δ_P_ (162 MHz, DMSO-*d*_6_): 22.23; ESI-HRMS (*m/z*): [M + H]^+^ calcd for C_12_H_13_N_2_O_4_PS, 312.0333; found, 312.0328.

#### 4-Sulfamoylphenyl *P*-Phenyl-*N*-propylphosphonamidate (**16**)

Compound **16** was obtained according to the general procedure earlier reported using propylamine (3.0 equiv), phenylphosphonic dichloride **2** (1.2 equiv), 4-hydroxybenzenesulfonamide **1** (0.3 g, 1.0 equiv), and trimethylamine (2.2 equiv) in dry THF (10 mL). The reaction mixture was concentrated *in vacuo*, and the obtained residue was purified by silica gel column chromatography eluting with 4% MeOH in DCM to afford the title compound as a white solid. 46% yield; mp 137–138 °C; silica gel TLC *R*_f_, 0.15 (MeOH/CH_2_Cl_2_ 5% v/v); δ_H_ (400 MHz, DMSO-*d*_6_): 0.79 (t, *J* = 7.4, 3H, CH_2_C*H*_3_), 1.38 (m, 2H, C*H*_2_CH_3_), 2.85 (m, 2H, NHC*H*_2_), 5.64 (m, 1H, exchange with D_2_O, PON*H*), 7.35 (s, 2H, exchange with D_2_O, SO_2_N*H*_2_), 7.43 (m, 2H, Ar-*H*), 7.60 (m, 3H, Ar-*H*), 7.86 (m, 4H, Ar-*H*); δ_C_ (100 MHz, DMSO-*d*_6_): 12.06, 25.42 (d, *J*^3^_CP_ = 5.5), 43.09, 121.77 (d, *J*^3^_CP_ = 4.9), 128.47, 129.50 (d, *J*^2^_CP_ = 14.5), 131.95 (d, *J*^3^_CP_ = 9.8), 132.09 (d, *J*^1^_CP_ = 173.1), 132.98 (d, *J*^4^_CP_ = 3.0), 140.78, 154.27 (d, *J*^2^_CP_ = 7.8); δ_P_ (162 MHz, DMSO-*d*_6_): 21.26; ESI-HRMS (*m/z*): [M + H]^+^ calcd for C_15_H_20_N_2_O_4_PS, 355.0881; found, 355.0880.

#### 4-Sulfamoylphenyl *N*,*N*-Diethyl-*P*-phenylphosphonamidate (**17**)

Compound **17** was obtained according to the general procedure earlier reported using diethylamine (3.0 equiv), phenylphosphonic dichloride **2** (1.2 equiv), 4-hydroxybenzenesulfonamide **1** (0.3 g, 1.0 equiv), and trimethylamine (2.2 equiv) in dry THF (10 mL). The reaction mixture was concentrated *in vacuo*, and the obtained residue was purified by silica gel column chromatography eluting with 4% MeOH in DCM to afford the title compound as a white solid. 35% yield; mp 130–131 °C; silica gel TLC *R*_f_, 0.40 (MeOH/CH_2_Cl_2_ 10% v/v); δ_H_ (400 MHz, DMSO-*d*_6_): 0.92 (t, *J* = 7.2, 6H, 2 × CH_2_C*H*_3_), 3.12 (m, 4H, 2 × C*H*_2_CH_3_), 7.38 (s, 2H, exchange with D_2_O, SO_2_N*H*_2_), 7.52 (m, 2H, Ar-*H*), 7.60 (m, 2H, Ar-*H*), 7.67 (m, 1H, Ar-*H*), 7.88 (m, 4H, Ar-*H*); δ_C_ (100 MHz, DMSO-*d*_6_): 14.63 (d, *J*^3^_CP_ = 2.0), 39.31 (d, *J*^2^_CP_ = 4.6), 121.58 (d, *J*^3^_CP_ = 5.0), 128.57, 129.68 (d, *J*^2^_CP_ = 14.4), 131.4 (d, *J*^1^_CP_ = 177.9), 131.91 (d, *J*^3^_CP_ = 9.9), 133.07 (d, *J*^4^_CP_ = 2.9), 140.96, 154.05 (d, *J*^2^_CP_ = 8.0); δ_P_ (162 MHz, DMSO-*d*_6_): 21.20; ESI-HRMS (*m/z*): [M + H]^+^ calcd for C_16_H_22_N_2_O_4_PS, 369.1038; found, 369.1032.

#### 4-Sulfamoylphenyl Phenyl(pyrrolidin-1-yl)phosphinate (**18**)

Compound **18** was obtained according to the general procedure earlier reported using pyrrolidine (3.0 equiv), phenylphosphonic dichloride **2** (1.2 equiv), 4-hydroxybenzenesulfonamide **1** (0.3 g, 1.0 equiv), and trimethylamine (2.2 equiv) in dry THF (10 mL). The reaction mixture was concentrated *in vacuo*, and the obtained residue was purified by silica gel column chromatography eluting with 4% MeOH in DCM to afford the title compound as a white solid. 58% yield; mp 118–119 °C; silica gel TLC *R*_f_, 0.56 (MeOH/CH_2_Cl_2_ 10% v/v); δ_H_ (400 MHz, DMSO-*d*_6_): 1.74 (m, 4H, 2 × CH_2_), 3.17 (m, 4H, 2 × C*H*_2_), 7.39 (s, 2H, exchange with D_2_O, SO_2_N*H*_2_), 7.50 (m, 2H, Ar-*H*), 7.60 (m, 2H, Ar-*H*), 7.67 (m, 1H, Ar-*H*), 7.87 (m, 4H, Ar-*H*); δ_C_ (100 MHz, DMSO-*d*_6_): 26.63 (d, *J*^2^_CP_ = 8.1), 47.31 (d, *J*^3^_CP_ = 4.4), 121.46 (d, *J*^3^_CP_ = 5.1), 128.66, 129.71 (d, *J*^2^_CP_ = 14.1), 130.44 (d, *J*^1^_CP_ = 173.2), 131.90 (d, *J*^3^_CP_ = 9.6), 133.19 (d, *J*^4^_CP_ = 2.9), 140.99, 154.06 (d, *J*^2^_CP_ = 7.5); δ_P_ (162 MHz, DMSO-*d*_6_): 18.85; ESI-HRMS (*m/z*): [M + H]^+^ calcd for C_16_H_20_N_2_O_4_PS, 367.0881; found, 367.0875.

#### 4-Sulfamoylphenyl Morpholino(phenyl)phosphinate (**19**)

Compound **19** was obtained according to the general procedure earlier reported using morpholine (3.0 equiv), phenylphosphonic dichloride **2** (1.2 equiv), 4-hydroxybenzenesulfonamide **1** (0.3 g, 1.0 equiv), and trimethylamine (2.2 equiv) in dry THF (10 mL). The reaction mixture was concentrated *in vacuo*, and the obtained residue was purified by silica gel column chromatography eluting with 4% MeOH in DCM to afford the title compound as a white solid. 49% yield; mp 125–126 °C; silica gel TLC *R*_f_, 0.15 (MeOH/CH_2_Cl_2_ 5% v/v); δ_H_ (400 MHz, DMSO-*d*_6_): 3.09 (m, 4H, 2 × CH_2_), 3.45 (m, 4H, 2 × C*H*_2_), 7.41 (s, 2H, exchange with D_2_O, SO_2_N*H*_2_), 7.54 (m, 2H, Ar-*H*), 7.63 (m, 2H, Ar-*H*), 7.70 (m, 1H, Ar-*H*), 7.90 (m, 4H, Ar-*H*); δ_C_ (100 MHz, DMSO-*d*_6_): 44.58, 66.95 (d, *J*^2^_CP_ = 5.1), 121.75 (d, *J*^3^_CP_ = 5.1), 128.73, 129.88 (d, *J*^2^_CP_ = 14.3), 130.00 (d, *J*^1^_CP_ = 177.6), 132.10 (d, *J*^3^_CP_ = 9.7), 133.47 (d, *J*^4^_CP_ = 3.0), 141.28, 153.83 (d, *J*^2^_CP_ = 8.0); δ_P_ (162 MHz, DMSO-*d*_6_): 19.24; ESI-HRMS (*m/z*): [M + H]^+^ calcd for C_16_H_20_N_2_O_5_PS, 383.0830; found, 383.0824.

#### 4-Sulfamoylphenyl (4-Methylpiperazin-1-yl)(phenyl)phosphinate (**20**)

Compound **20** was obtained according to the general procedure earlier reported using *N*-methylpiperazine (3.0 equiv), phenylphosphonic dichloride **2** (1.2 equiv), 4-hydroxybenzenesulfonamide **1** (0.3 g, 1.0 equiv), and trimethylamine (2.2 equiv) in dry THF (10 mL). The reaction mixture was concentrated *in vacuo*, and the obtained residue was purified by silica gel column chromatography eluting with 4% MeOH in DCM to afford the title compound as a white solid. 41% yield; mp 135–136 °C; silica gel TLC *R*_f_, 0.09 (MeOH/CH_2_Cl_2_ 10% v/v); δ_H_ (400 MHz, DMSO-*d*_6_): 2.11 (s, 3H, NC*H*_3_), 2.16 (m, 4H, 2 × CH_2_), 3.11 (m, 4H, 2 × C*H*_2_), 7.41 (s, 2H, exchange with D_2_O, SO_2_N*H*_2_), 7.53 (m, 2H, Ar-*H*), 7.62 (m, 2H, Ar-*H*), 7.69 (m, 1H, Ar-*H*), 7.88 (m, 4H, Ar-*H*); δ_C_ (100 MHz, DMSO-*d*_6_): 44.33 (d, *J*^3^_CP_ = 1.8), 46.80, 55.20 (d, *J*^3^_CP_ = 5.4), 121.67 (d, *J*^3^_CP_ = 4.9), 128.64, 129.76 (d, *J*^2^_CP_ = 14.5), 130.39 (d, *J*^1^_CP_ = 177.7), 132.00 (d, *J*^3^_CP_ = 9.9), 133.30 (d, *J*^4^_CP_ = 2.9), 141.18, 153.89 (d, *J*^2^_CP_ = 8.0); δ_P_ (162 MHz, DMSO-*d*_6_): 19.65; ESI-HRMS (*m/z*): [M + H]^+^ calcd for C_17_H_23_N_3_O_4_PS, 396.1146; found, 396.1145.

#### 4-Sulfamoylphenyl *N*-Benzyl-*P*-phenylphosphonamidate (**21**)

Compound **21** was obtained according to the general procedure earlier reported using benzylamine (3.0 equiv), phenylphosphonic dichloride **2** (1.2 equiv), 4-hydroxybenzenesulfonamide **1** (0.3 g, 1.0 equiv), and trimethylamine (2.2 equiv) in dry THF (10 mL). The reaction mixture was concentrated *in vacuo*, and the obtained residue was purified by silica gel column chromatography eluting with 4% MeOH in DCM to afford the title compound as a white solid. 63% yield; mp 185–186 °C; silica gel TLC *R*_f_, 0.40 (MeOH/CH_2_Cl_2_ 20% v/v); δ_H_ (400 MHz, DMSO-*d*_6_): 4.14 (m, 2H, NHC*H*_2_), 6.23 (m, exchange with D_2_O, PON*H*), 7.26 (m, 5H, Ar-*H*), 7.36 (s, 2H, exchange with D_2_O, SO_2_N*H*_2_), 7.40 (m, 2H, Ar-*H*), 7.57 (m, 2H, Ar-*H*), 7.645 (m, 1H, Ar-*H*), 7.81 (m, 2H, Ar-*H*), 7.88 (m, 2H, Ar-*H*); δ_C_ (100 MHz, DMSO-*d*_6_): 44.66, 121.90 (d, *J*^3^_CP_ = 4.5), 127.69, 128.09, 128.47, 129.05, 129.51 (d, *J*^2^_CP_ = 14.5), 131.85 (d, *J*^1^_CP_ = 173.5), 132.01 (d, *J*^3^_CP_ = 10.2), 133.01 (d, *J*^4^_CP_ = 2.9), 140.91, 141.20 (d, *J*^3^_CP_ = 5.2), 154.15 (d, *J*^2^_CP_ = 7.5); δ_P_ (162 MHz, DMSO-*d*_6_): 21.26; ESI-HRMS (*m/z*): [M + H]^+^ calcd for C_19_H_20_N_2_O_4_PS, 403.0881; found, 403.0875.

#### 4-Sulfamoylphenyl *N*,*P*-Diphenylphosphonamidate (**22**)

Compound **22** was obtained according to the general procedure earlier reported using aniline (3.0 equiv), phenylphosphonic dichloride **2** (1.2 equiv), 4-hydroxybenzenesulfonamide **1** (0.3 g, 1.0 equiv), and trimethylamine (2.2 equiv) in dry THF (10 mL). The reaction mixture was concentrated *in vacuo*, and the obtained residue was purified by silica gel column chromatography eluting with 4% MeOH in DCM to afford the title compound as a white solid. 32% yield; mp 187–188 °C; silica gel TLC *R*_f_, 0.38 (MeOH/CH_2_Cl_2_ 10% v/v); δ_H_ (400 MHz, DMSO-*d*_6_): 6.91 (m, 1H, Ar-*H*), 7.13 (m, 2H, Ar-*H*), 7.21 (m, 2H, Ar-*H*), 7.36 (s, 2H, exchange with D_2_O, SO_2_N*H*_2_), 7.46 (m, 2H, Ar-*H*), 7.60 (m, 2H, Ar-*H*), 7.67 (m, 1H, Ar-*H*), 7.85 (m, 2H, Ar-*H*), 7.93 (m, 2H, Ar-*H*); δ_C_ (100 MHz, DMSO-*d*_6_): 118.86 (d, *J*^3^_CP_ = 6.9), 121.75 (d, *J*^3^_CP_ = 4.5), 122.21, 128.61, 129.73 (d, *J*^2^_CP_ = 14.5), 129.96, 130.62 (d, *J*^1^_CP_ = 172.9), 132.32 (d, *J*^2^_CP_ = 10.4), 133.64 (d, *J*^4^_CP_ = 2.5), 141.29, 141.39 (d, *J*^4^_CP_ = 1.2), 153.61 (d, *J*^2^_CP_ = 7.6); δ_P_ (162 MHz, DMSO-*d*_6_): 15.37; ESI-HRMS (*m/z*): [M + H]^+^ calcd for C_18_H_18_N_2_O_4_PS ,389.0724; found, 389.0716.

### Carbonic Anhydrase Inhibition

An Applied Photophysics stopped-flow instrument has been used for assaying the CA-catalyzed CO_2_ hydration activity.^32^ Phenol red (at a concentration of 0.2 mM) has been used as an indicator, working at the absorbance maximum of 557 nm, with 20 mM HEPES (pH 7.5) as a buffer and 20 mM Na_2_SO_4_ (for maintaining constant the ionic strength), following the initial rates of the CA-catalyzed CO_2_ hydration reaction for a period of 10–100 s. The CO_2_ concentrations ranged from 1.7 to 17 mM for the determination of the kinetic parameters and inhibition constants. For each inhibitor, at least six traces of the initial 5–10% of the reaction have been used for determining the initial velocity. The uncatalyzed rates were determined in the same manner and subtracted from the total observed rates. Stock solutions of the inhibitor (0.1 mM) were prepared in distilled-deionized water, and dilutions up to 0.01 nM were done thereafter with the assay buffer. Inhibitor and enzyme solutions were preincubated together for 1 h at room temperature prior to the assay, to allow for the formation of the E–I complex. The inhibition constants were obtained by nonlinear least-squares methods using PRISM 3 and the Cheng–Prusoff equation, as reported earlier,^43,44^ and represent the mean from at least three different determinations. All CA isoforms were recombinant ones obtained in-house as reported earlier.^30^

### Drug Stability Study

#### Chemicals

Acetonitrile (Chromasolv), methanol (Chromasolv), formic acid (MS grade), ammonium formate, HCl, NaCl, KCl, Na_2_HPO_4_·2H_2_O, KH_2_PO_4_ (Reagent grade), and verapamil hydrochloride (analytical standard, used as the internal standard) were purchased from Sigma-Aldrich (Milan, Italy). MilliQ water 18 MΩ was obtained from the Millipore’s Simplicity system (Milan, Italy). Phosphate buffer solution (PBS) was prepared by adding 8.01 g L^–1^ NaCl, 0.2 g L^–1^ KCl, 1.78 g L^–1^ Na_2_HPO_4_·2H_2_O, and 0.27 g L^–1^ KH_2_PO_4_. Human plasma was collected from a healthy male volunteer; each plasma batch was kept at −80 °C until use.

#### Instrumental

The LC–MS/MS analysis was carried out using a Varian 1200L triple quadrupole system (Palo Alto, CA, USA) equipped with two ProStar 210 pumps, a ProStar 410 autosampler, and an electrospray source (ESI) operating in positive ions. Raw data were collected and processed by Varian Workstation version 6.8 software. A G-Therm 015 thermostatic oven was used to maintain the samples at 37 °C during the test of degradation. An ALC microcentrifuge 4214 was employed to centrifuge the samples.

#### LC–MS/MS Methods

The chromatographic parameters employed to analyze the samples were tuned to minimize the run time. The column having a PFP 30 mm length, 2 mm internal diameter, and 3 μm particle size was used, at a constant flow of 0.25 mL min^–1^, employing a binary mobile phase elution gradient. The eluents used were 10 mM formic acid and 5 mM ammonium formate in water solution (solvent A) and 10 mM ammonium formate and 5 mM formic acid in methanol (solvent B) according to the elution gradient as follows: initial at 90% solvent A, which was then decreased to 10% in 4.0 min, kept for 3.0 min, returned to initial conditions in 0.1 min, and maintained for 3.0 min for reconditioning, to a total run time of 10.0 min.

The column temperature was maintained at 30 °C, and the injection volume was 5 μL. The ESI source was operated in the positive ion mode, using the following setting: 5 kV needle, 42 psi nebulizing gas, 600 V shield, and 20 psi drying gas at 280 °C. The analyses were acquired in multiple reaction monitoring (MRM), and the ion transitions are reported in Table 3.

**Table 3:** MRM Parameters

compound	precursor ion (*m/z*)	quantifier ion (*m/z*) [CE (V)]	qualifier ion (*m/z*) [CE (V)]
**6**	356	297 [20]	314 [10]
**7**	352	335 [15]	131 [25]
**10**	469	452 [15]	280 [35]
**18**	367	194 [30]	350 [20]
**20**	396	223 [25]	296 [40]
**22**	389	308 [20]	296 [30]
ISTD	455	165 [25]	303 [25]

#### Standard Solutions

Stock solutions of analytes and verapamil hydrochloride (internal standard or ISTD) were prepared in acetonitrile at 1.0 mg mL^–1^ and stored at 4 °C. The ISTD working solution was prepared in acetonitrile at 15 μg mL^–1^ (ISTD solution). The spiked solutions of each analyte were prepared separately, by diluting the respective stock solutions in mQ water/acetonitrile 80:20 (v/v) solution, to obtain a final concentration of 10 μM.

#### Sample Preparation

The sample was prepared by adding 10 μL of spiked solution to 100 μL of tested matrix (PBS or human plasma or 10 mM HCl) in microcentrifuge tubes. The obtained solutions correspond to 1 μM analyte. Each set of samples was incubated in triplicate at four different times: 0, 30, 60, and 120 min at 37 °C. Therefore, the matrix stability profile of each analyte was represented by a batch of 12 samples (4 incubation times × 3 replicates). After the incubation, the samples were added with 300 μL of ISTD solution and centrifuged (room temperature for 5 min at 800*g*). The supernatants were transferred in autosampler vials and added with 0.6 mL of mQ water. The final solutions were analyzed by the LC–MS/MS methods described above.

### X-ray Crystallography

Native hCA II was produced and purified as previously described.^45^ For hCA VII, a mutated form where the cysteine residues in positions 183 and 217 were mutated to serines was used for crystallographic studies since we previously reported that this mutant was more suitable for crystallization experiments.^46^ For both enzymes, crystals were obtained by the hanging drop vapor diffusion technique, using a precipitant solution containing 1.3 M sodium citrate and 60 mM Tris-HCl, pH 8.0 for CA II and 25% (w/v) polyethylene glycol 3350, 0.2 M ammonium acetate, and 0.1 M Tris-HCl, pH 8.5 for CA VII. Crystals of enzyme–inhibitor complexes were prepared by soaking enzyme crystals in precipitant solutions containing 10 mM inhibitor and using 1/18 h as the soaking time for CA II/VII crystals, respectively.

X-ray diffraction data for both CA II/**3** and CA VII/**3** adducts were collected at 100 K, using a Rigaku MicroMax-007 HF generator producing Cu Kα radiation and equipped with a Saturn 944 CCD detector. Prior to cryogenic freezing, crystals were transferred to the respective precipitant solution with the addition of 10/25% (v/v) glycerol for hCA II/VII. Data were integrated, merged, and scaled using HKL2000.^47^ Crystal parameters and data collection statistics for both adducts are reported in Table S1.

Phasing and refinement of the complexes were carried out with REFMAC5^48^ using as starting models the previously solved hCA II (PDB ID: 6H29)^33^ and hCA VII (PDB ID: 6G4T)^46^ structures with water molecules and ligands removed. In both structures, the inhibitor molecule was clearly visible in the difference Fo – Fc map from the early stages of refinement. Restraints on inhibitor bond angles and distances were taken from the Cambridge Structural Database,^49^ and topology file was generated using the PRODRG2 server.^50^ Standard restraints were used on protein bond angles and distances throughout refinement. Several rounds of manual rebuilding were performed using the program O^51^ with careful inspection of the 2Fo – Fc and Fo – Fc electron density maps. The geometric restraints of the final models were analyzed using the programs PROCHECK^52^ and WHATCHECK.^53^ The refinement statistics of the final models are summarized in Table S1. The atomic coordinates of the complexes have been deposited in the Protein Data Bank with accession codes 6SDS and 6SDT.

### Animals

Male CD-1 albino mice (Envigo, Varese, Italy) weighing approximately 22–25 g at the beginning of the experimental procedure were used. Animals were housed in Ce.S.A.L (Centro Stabulazione Animali da Laboratorio, University of Florence) and used at least 1 week after their arrival. Ten mice were housed per cage (size 26 × 41 cm); animals were fed a standard laboratory diet and tap water *ad libitum* and kept at 23 ± 1 °C with a 12 h light/dark cycle, light at 7 a.m. All animal manipulations were carried out according to the Directive 2010/63/EU of the European Parliament and of the European Union Council (22 September 2010) on the protection of animals used for scientific purposes. The ethical policy of the University of Florence complies with the Guide for the Care and Use of Laboratory Animals of the US National Institutes of Health (NIH publication no. 85-23, revised 1996; University of Florence assurance number: A5278-01). Formal approval to conduct the experiments described was obtained from the Animal Subjects Review Board of the University of Florence. Experiments involving animals have been reported according to ARRIVE guidelines.^54^ All efforts were made to minimize animal suffering and to reduce the number of animals used.

#### Oxaliplatin-Induced Neuropathic Pain Model and Pharmacological Treatments

Mice treated with oxaliplatin (2.4 mg kg^–1^) were administered intraperitoneally (i.p.) on days 1 and2, 5–9, and 12–14 (10 i.p. injections).^55^ Oxaliplatin was dissolved in 5% glucose solution. Control animals received an equivalent volume of vehicle. Behavioral tests were performed starting from day 15. Acetazolamide (**AAZ**; 100 mg kg^–1^), duloxetine (15 mg kg^–1^), compounds **6**, **7**, **10**, **20**, and **22** (10–100 mg kg^–1^), and enantiomers (***R***)-**7** and (***S***)-**7**, both 50 mg kg^–1^, were suspended in 1% carboxymethylcellulose sodium salt (CMC, Sigma-Aldrich, Milan, Italy) and per os (p.o.) acutely administered. Behavioral tests were carried out before and after (15, 30, 45, and 60 min) compound’s injection.

#### Cold Plate

Thermal allodynia was assessed using the cold plate test. With minimal animal–handler interaction, mice were taken from home cages and placed onto the surface of the cold plate (Ugo Basile, Varese, Italy) maintained at a constant temperature of 4 °C ± 1 °C. Ambulation was restricted by a cylindrical Plexiglas chamber (diameter: 10 cm; height: 15 cm), with an open top. A timer controlled by foot pedal began timing response latency from the moment the mouse was placed onto the cold plate. Pain-related behavior (licking of the hind paw) was observed, and the time (seconds) of the first sign was recorded. The cutoff time of the latency of paw lifting or licking was set at 30 s.^56^

#### Statistical Analysis

Behavioral measurements were performed on 12 mice for each treatment carried out in two different experimental sets. Results were expressed as means ± SEM. The analysis of variance of behavioral data was performed by one-way ANOVA, and a Bonferroni’s significant difference procedure was used for post-hoc comparison. *P* values of less than 0.05 or 0.01 were considered significant. Investigators were blind to all experimental procedures. Data were analyzed using the “Origin 9” software (OriginLab, Northampton, USA).

### Enantioseparation and Assignment of Absolute Configuration

Semipreparative HPLC separations of the enantiomers of **7** was carried out on the commercially available 250 mm × 10 mm I.D. CHIRALPAK IA (Chiral Technologies Europe, Illkirch, France) column using the mixture *n*-hexane/EtOH/MeOH/TFA 60:35:5:0.1 (v/v/v/v) as a mobile phase. The temperature was set at 15 °C. The flow rate was 4.5 mL min^–1^. HPLC apparatus consisted of a PerkinElmer (Norwalk, CT, USA) 200 LC pump equipped with a Rheodyne (Cotati, CA, USA) injector, a 5 mL sample loop, an HPLC PerkinElmer oven, and a PerkinElmer detector. The signal was acquired and processed by Clarity software (DataApex, Prague, Czech Republic). The amount of racemic samples resolved for single chromatographic run was 1 mg.

The CD spectra of enantiomers of **7**, dissolved in ethanol (concentration about 0.40 mg mL^–1^) in a 0.1 cm path length quartz cell at 25 °C, were measured by using a Jasco model J-700 spectropolarimeter. The spectra are average computed over three instrumental scans, and the intensities are presented in terms of ellipticity values (mdeg).

Specific rotations were measured at 589, 578, 546, 436, 365, and 302 nm by a PerkinElmer polarimeter model 241 equipped with Na/Hg lamps. The volume of the cell was 1 mL, and the optical path was 10 cm. The system was set at a temperature of 20 °C.

The *in silico* procedure adopted to achieve the assignment of absolute configuration can be summarized in the following five steps: (i) conduction of a conformational search based on molecular mechanic calculations (MM) performed on the structure of **7** characterized by (*R*)-configuration followed by the selection of the more representative conformations inside an energetic window of 4 kcal mol^–1^; (ii) further optimization at the AM1 semiempirical level of theory of the conformations selected from the MM search again followed by the selection of the structures found inside an energetic window of 1.2 kcal mol^–1^(four conformations), which corresponds to an overall Boltzmann population of 93.6%; (iii) energy minimization performed in a vacuum at the B3LYP/6-31G* level of theory of the four AM1 structures coming from the previous step, which collapsed to just one final conformation (maximum difference in energy stability amounting to 0.06 kcal mol^–1^ and root-mean-square deviation (RMSD) of atomic positions, with exclusion of hydrogens, lesser than 0.3 Å); (iv) conclusive optimization at the M06/6-31G* level of theory of the most stable conformation found in the previous B3LYP minimization step; (v) quantum mechanical simulation of both electronic circular dichroism (ECD) and optical rotatory dispersion (ORD) assessed for the conformation of **7** of (*R*)-configuration achieved in the previous fourth step.

The conformational search quoted in the first step of the procedure was carried out through MM calculations (force field: MMFF94), according to the systematic algorithm implemented in the computer program Spartan 10 v.1.1.0 (Wavefunction Inc., 18401 Von Karman Avenue, Suite 370, Irvine, CA 92612, USA). The conditions adopted in this analysis were as follows: (a) all the rotatable bonds varied; (b) 40 kJ mol^–1^ as the maximum energy gap from the lowest energy geometry imposed for kept conformations; (c) *R*^2^ ≥ 0.9, the criterion adopted to define conformers as duplicates in the analysis of similarity between conformations. Similarly, the structure optimizations mentioned in steps from 2 to 4 were again carried out by means of the program Spartan 10 v.1.1.0.

Instead, simulation of both ECD and ORD spectra has been performed through the algorithms implemented in the Amsterdam Density Functional (ADF) package v. 2007.01. The options set for such calculations were as follows: single point at the BLYP level of theory, employing the QZ4P large core basis set; ethanol as the solvent; 30 singlet and triplet excitations; the diagonalization method: Davidson; velocity representation; scaling factor, 0.90; and peak width, 8.0. Optical rotation values, [α]*_n_*, have been assessed at six different wavelengths (*n*), in the range 400–589 nm. The found values, scaled of a factor 10, were as follows: [α]_400_ = 45.5°; [α]_438_ = 33.6°; [α]_476_ = 26.1°; [α]_513_ = 21.1°; [α]_551_ = 17.6°; and [α]_589_ = 14.8°.

The final simulated ECD and ORD spectra of (***R***)-**7**, superimposed on the experimental ones corresponding to the isolated enantiomers of **7**, have been reported in Figure 7B,C. By inspection of the resulting plots, it can be reasonably inferred that to the first and second eluted enantiomers of **7** (green and red lines in Figure 7B,C) have to be assigned the configurations (*R*) and (*S*), respectively.

## Supporting Information Available

Data for 3HS4 (PDB)
Data for 3ML5 (PDB)
Data for 6SDS (PDB)
Data for 6SDT (PDB)
SMILES representation for compounds **3**–**22** (CSV)
^1^H, ^13^C, and ^31^P NMR spectra, crystallographic data collection and refinement statistics, drug stability profiles in solution, HPLC chromatograms, and *in silico*-predicted ADMET properties (PDF)

## Supplementary Material

jm9b02135_si_001.pdb

jm9b02135_si_002.pdb

jm9b02135_si_003.pdb

jm9b02135_si_004.pdb

## Notes

The atomic coordinates of the complexes have been deposited in the Protein Data Bank with accession codes 6SDS and 6SDT.

## Abbreviations Used

NP: neuropathic pain
CAI: carbonic anhydrase inhibitor
MOP: μ-opioid receptor
H4R: histamine H4 receptor
KCC2: potassium chloride cotransporter 2
SAR: structure–activity relationship
THF: tetrahydrofuran
CMC: carboxymethylcellulose
