# Supplementary material for: Phenyl(thio)phosphon(amid)ate Benzenesulfonamides as Potent and Selective Inhibitors of Human Carbonic Anhydrases II and VII Counteract Allodynia in a Mouse Model of Oxaliplatin-Induced Neuropathy
Source: J Med Chem. 2020 May 4;63(10):5185–200. doi: 10.1021/acs.jmedchem.9b02135 (PMC8007106; doi:10.1021/acs.jmedchem.9b02135)
Supplement: Supplementary file 6 — jm9b02135_si_006.pdf [file jm9b02135_si_006.pdf]

Supporting Information

Phenyl(thio)phosphon(amid)ate Benzenesulfonamides as Potent and Selective Inhibitors of Human Carbonic Anhydrases II and VII Counteract Allodynia in a Mouse Model of Oxaliplatin-Induced Neuropathy

Alessio Nocentini*, Vincenzo Alterio, Silvia Bua, Laura Micheli, Davide Esposito, Martina Buonanno, Gianluca Bartolucci, Sameh M. Osman, Zeid A. ALOthman, Roberto Cirilli, Marco Pierini, Simona Maria Monti, Lorenzo Di Cesare Mannelli, Paola Gratteri, Carla Ghelardini, Giuseppina De Simone, Claudiu T. Supuran*

^{1}H-, ^{13}C-, ^{31}P-NMR spectra S2

Crystallographic data collection and refinement statistics S32

Drug stability profiles in solution S33

HPLC chromatograms S35

In silico* predicted ADMET properties for compounds tested *in vivo S44

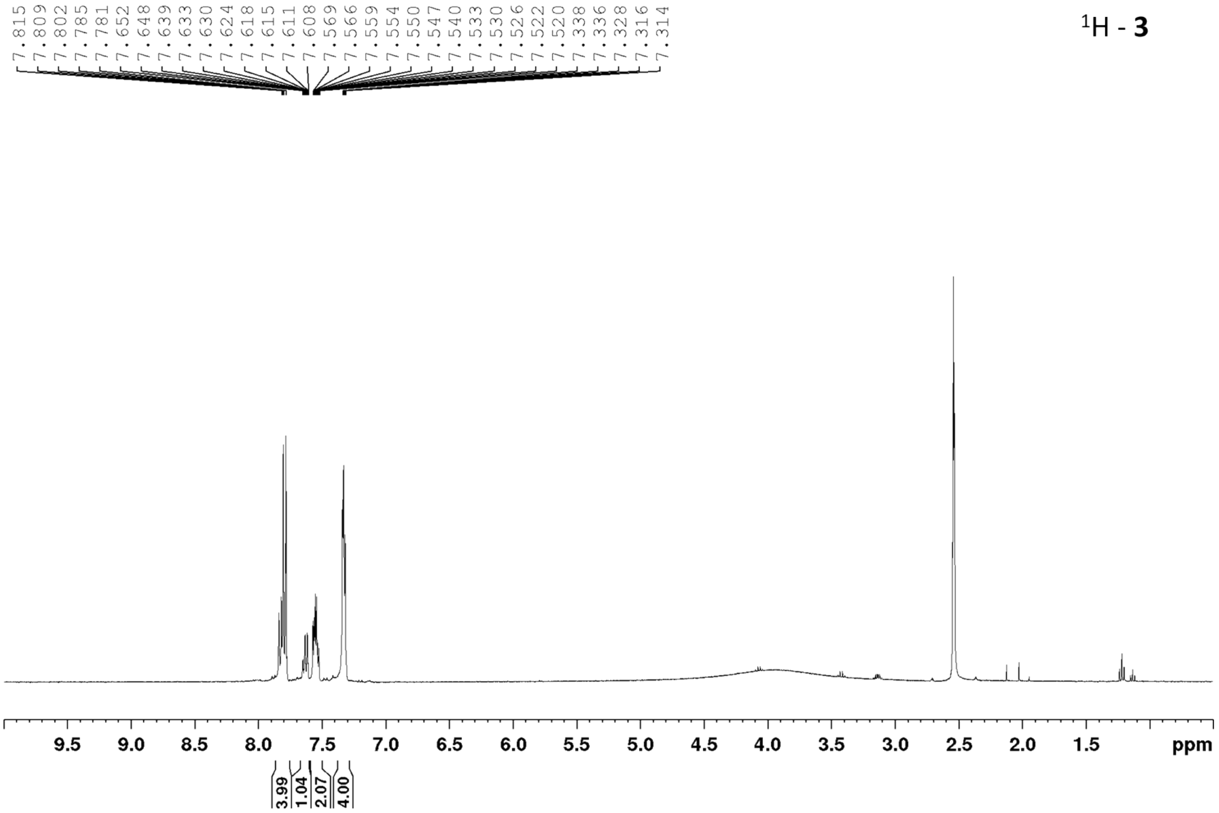
7.815 7.809 7.802 7.785 7.781 7.652 7.648 7.639 7.633 7.630 7.624 7.618 7.615 7.611 7.608 7.569 7.566 7.559 7.554 7.550 7.547 7.540 7.533 7.530 7.526 7.522 7.520 7.338 7.336 7.328 7.316 7.314
^{1}H - **3**
9.5 9.0 8.5 8.0 7.5 7.0 6.5 6.0 5.5 5.0 4.5 4.0 3.5 3.0 2.5 2.0 1.5 ppm
3.99 1.04 2.07 4.00

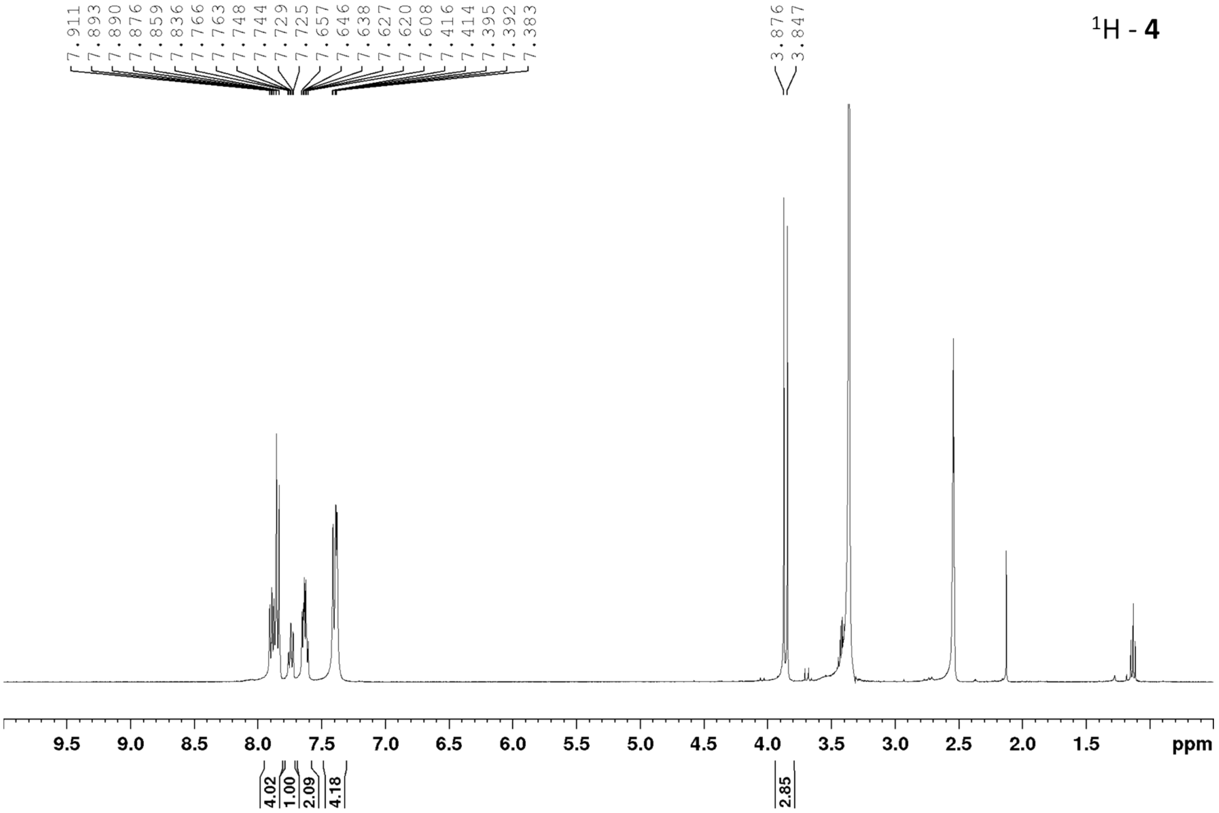
7.911 7.893 7.890 7.876 7.859 7.836 7.766 7.763 7.748 7.744 7.729 7.725 7.657 7.646 7.638 7.627 7.620 7.608 7.416 7.414 7.395 7.392 7.383
3.876 3.847
^{1}H - **4**
9.5 9.0 8.5 8.0 7.5 7.0 6.5 6.0 5.5 5.0 4.5 4.0 3.5 3.0 2.5 2.0 1.5 ppm
4.02 1.00 2.09 4.18
2.85

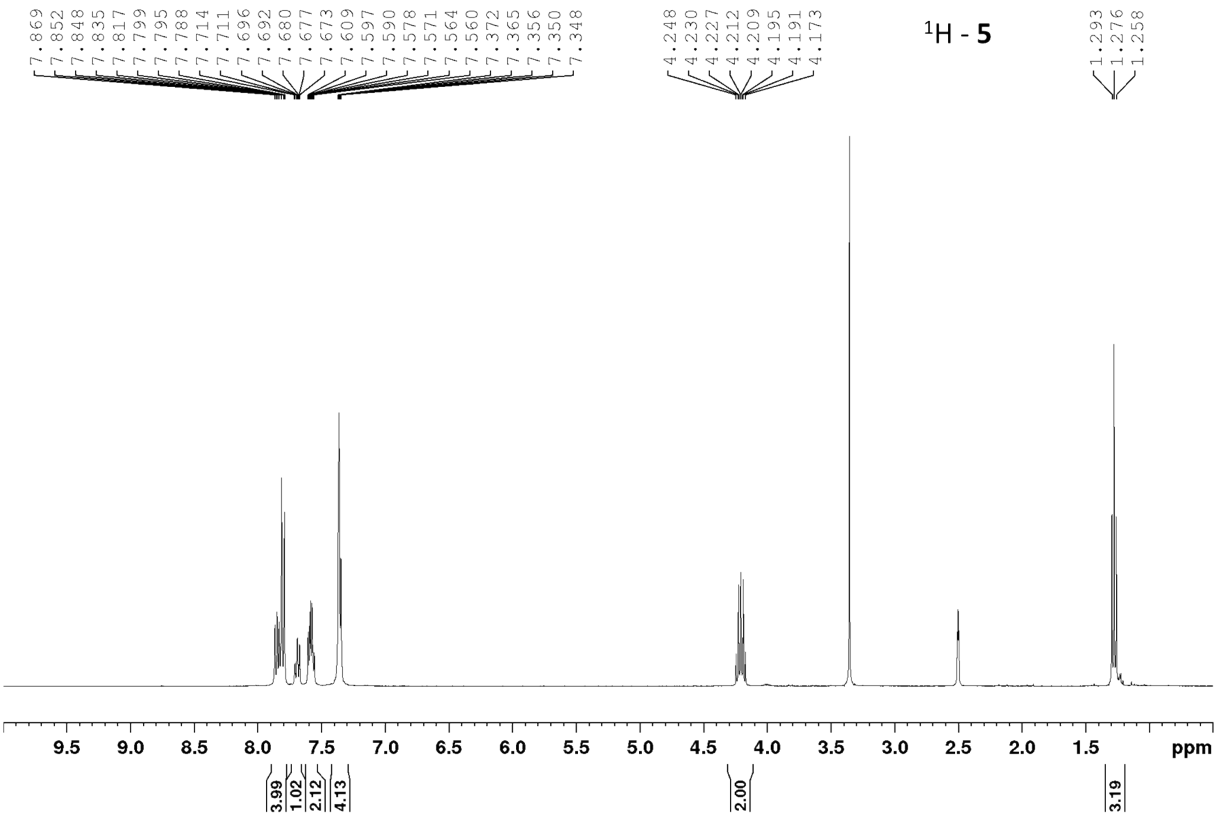
^{1}H - 5
7.869 7.852 7.848 7.835 7.817 7.799 7.795 7.788 7.714 7.711 7.696 7.692 7.680 7.677 7.673 7.609 7.597 7.590 7.578 7.571 7.564 7.560 7.372 7.365 7.356 7.350 7.348
4.248 4.230 4.227 4.212 4.209 4.195 4.191 4.173
1.293 1.276 1.258
9.5 9.0 8.5 8.0 7.5 7.0 6.5 6.0 5.5 5.0 4.5 4.0 3.5 3.0 2.5 2.0 1.5 ppm
3.99 1.02 2.12 4.13 2.00 3.19

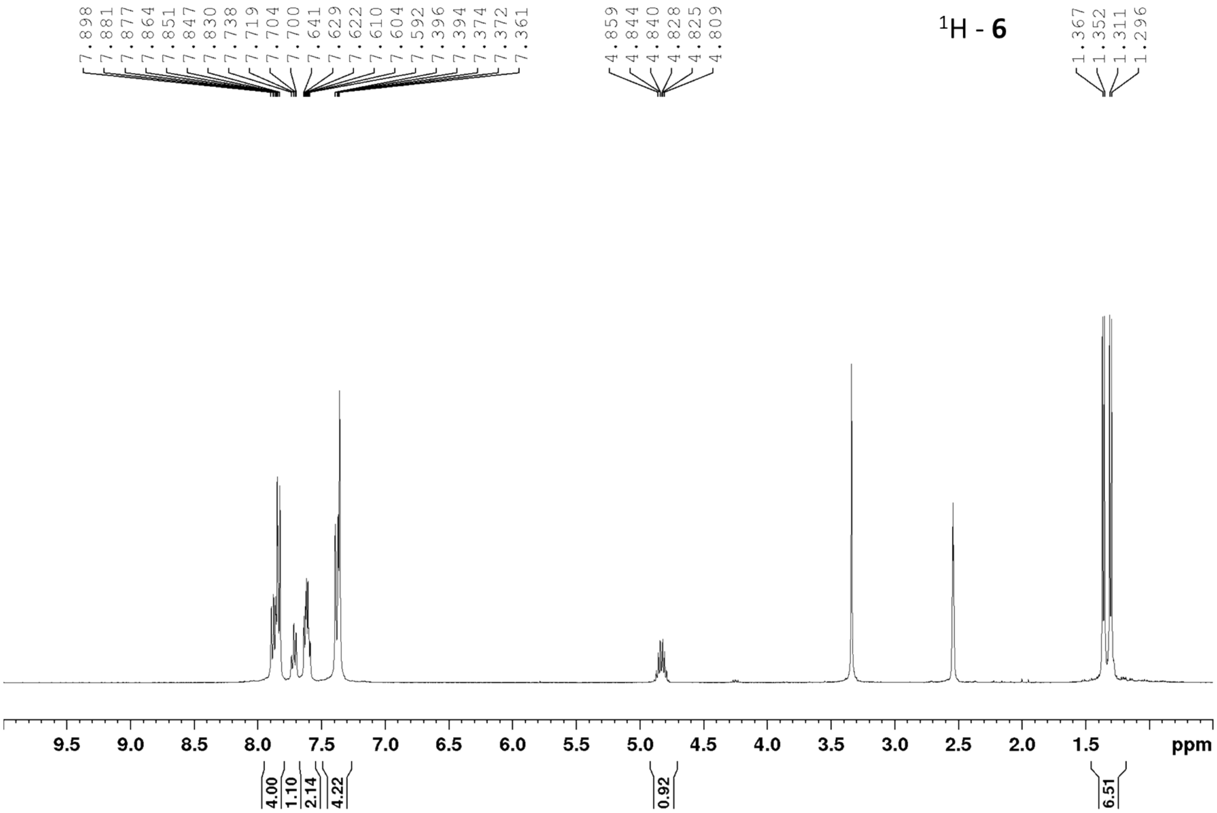
^{1}H - 6
7.898 7.881 7.877 7.864 7.851 7.847 7.830 7.738 7.719 7.704 7.700 7.641 7.629 7.622 7.610 7.604 7.592 7.396 7.394 7.374 7.372 7.361
4.859 4.844 4.840 4.828 4.825 4.809
1.367 1.352 1.311 1.296
9.5 9.0 8.5 8.0 7.5 7.0 6.5 6.0 5.5 5.0 4.5 4.0 3.5 3.0 2.5 2.0 1.5 ppm
4.00 1.10 2.14 4.22 0.92 6.51

^{1}H - **7**

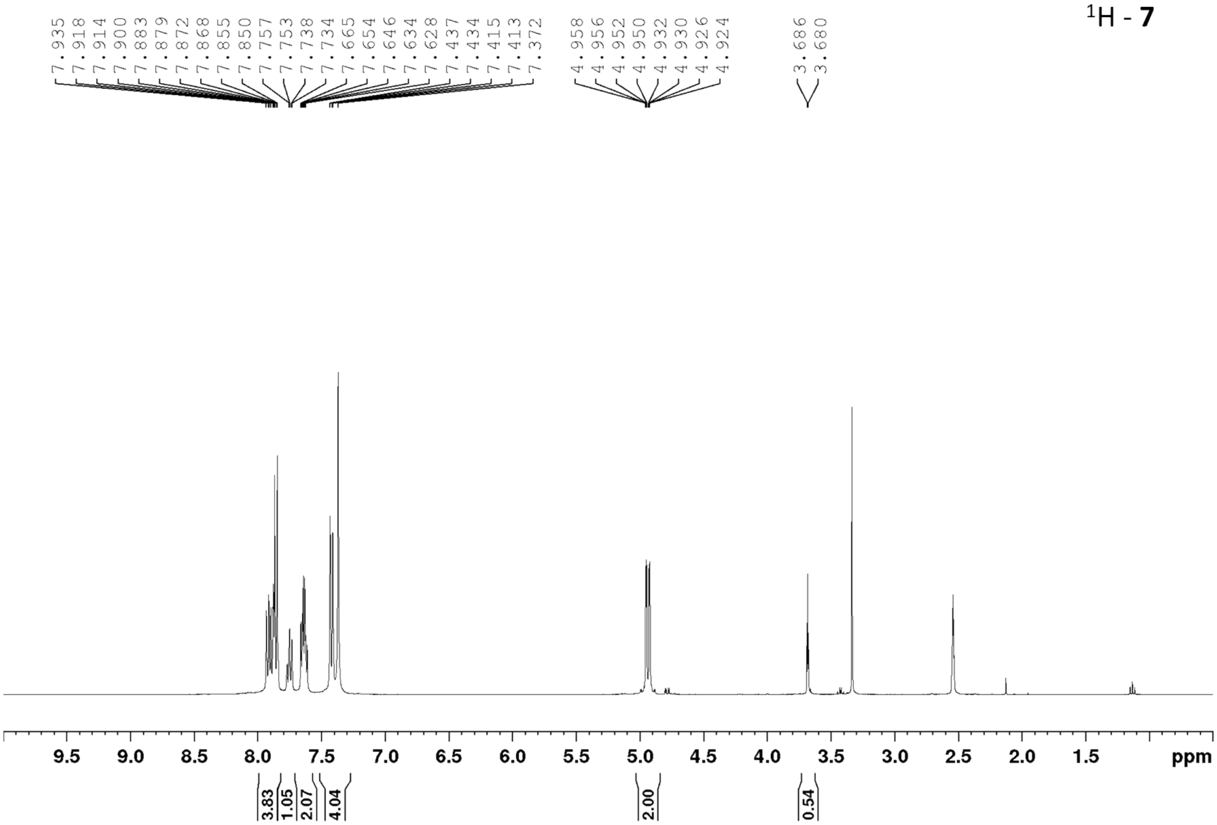
7.935
7.918
7.914
7.900
7.883
7.879
7.872
7.868
7.855
7.850
7.757
7.753
7.738
7.734
7.665
7.654
7.646
7.634
7.628
7.437
7.434
7.415
7.413
7.372
4.958
4.956
4.952
4.950
4.932
4.930
4.926
4.924
3.686
3.680
9.5 9.0 8.5 8.0 7.5 7.0 6.5 6.0 5.5 5.0 4.5 4.0 3.5 3.0 2.5 2.0 1.5 ppm
3.83
1.05
2.07
4.04
2.00
0.54

^{1}H - **8**

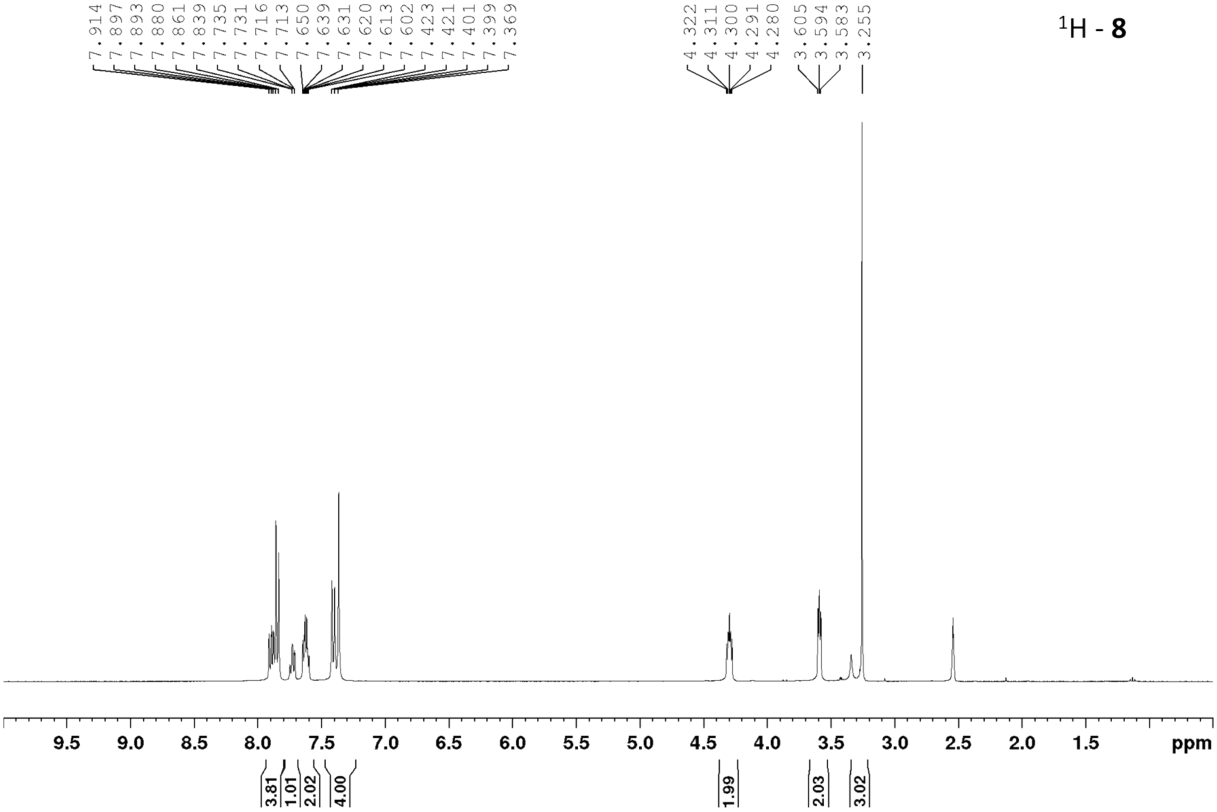
7.914
7.897
7.893
7.880
7.861
7.839
7.735
7.731
7.716
7.713
7.650
7.639
7.631
7.620
7.613
7.602
7.423
7.421
7.401
7.399
7.369
4.322
4.311
4.300
4.291
4.280
3.605
3.594
3.583
3.255
9.5 9.0 8.5 8.0 7.5 7.0 6.5 6.0 5.5 5.0 4.5 4.0 3.5 3.0 2.5 2.0 1.5 ppm
3.81
1.01
2.02
4.00
1.99
2.03
3.02

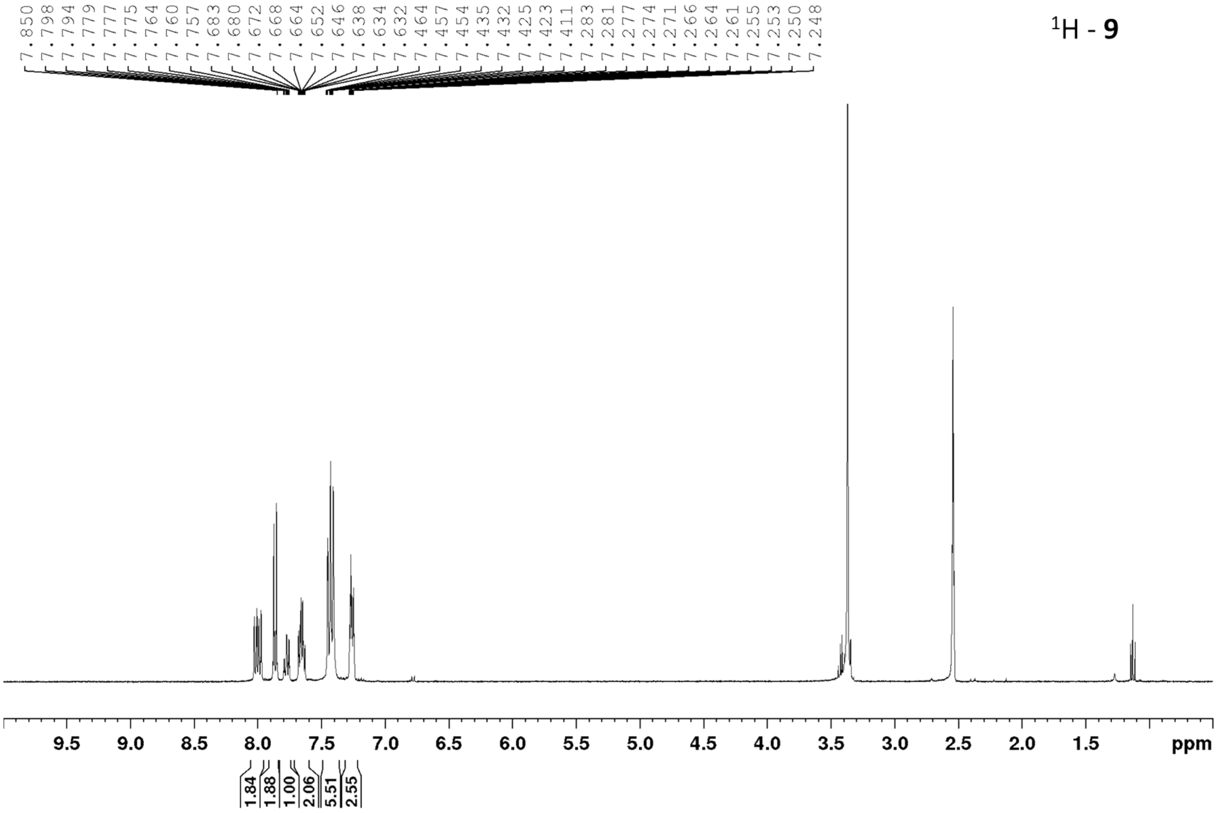
ppm
9.5
9.0
8.5
8.0
7.5
7.0
6.5
6.0
5.5
5.0
4.5
4.0
3.5
3.0
2.5
2.0
1.5
1.84
1.88
1.00
2.06
5.51
2.55

^1H - **9**

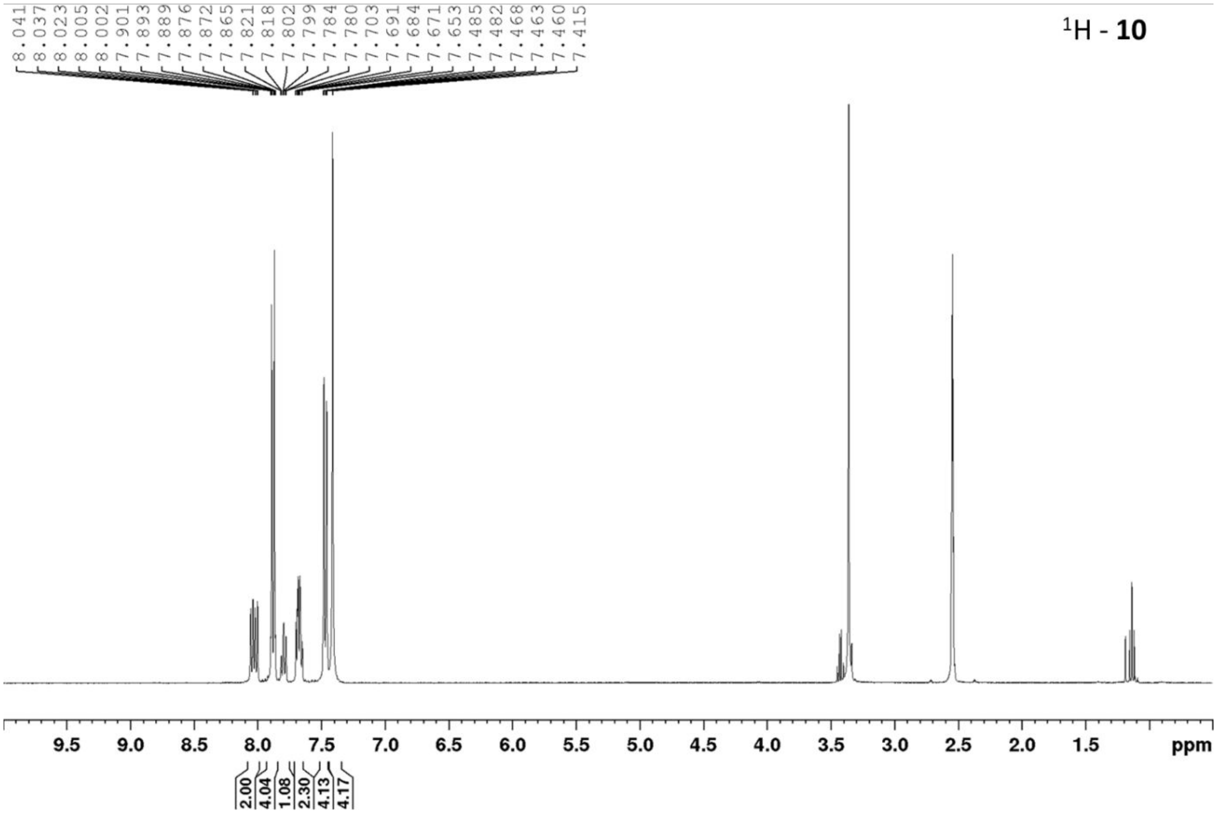
ppm
9.5
9.0
8.5
8.0
7.5
7.0
6.5
6.0
5.5
5.0
4.5
4.0
3.5
3.0
2.5
2.0
1.5
2.00
4.04
1.08
2.30
4.13
4.17

^1H - **10**

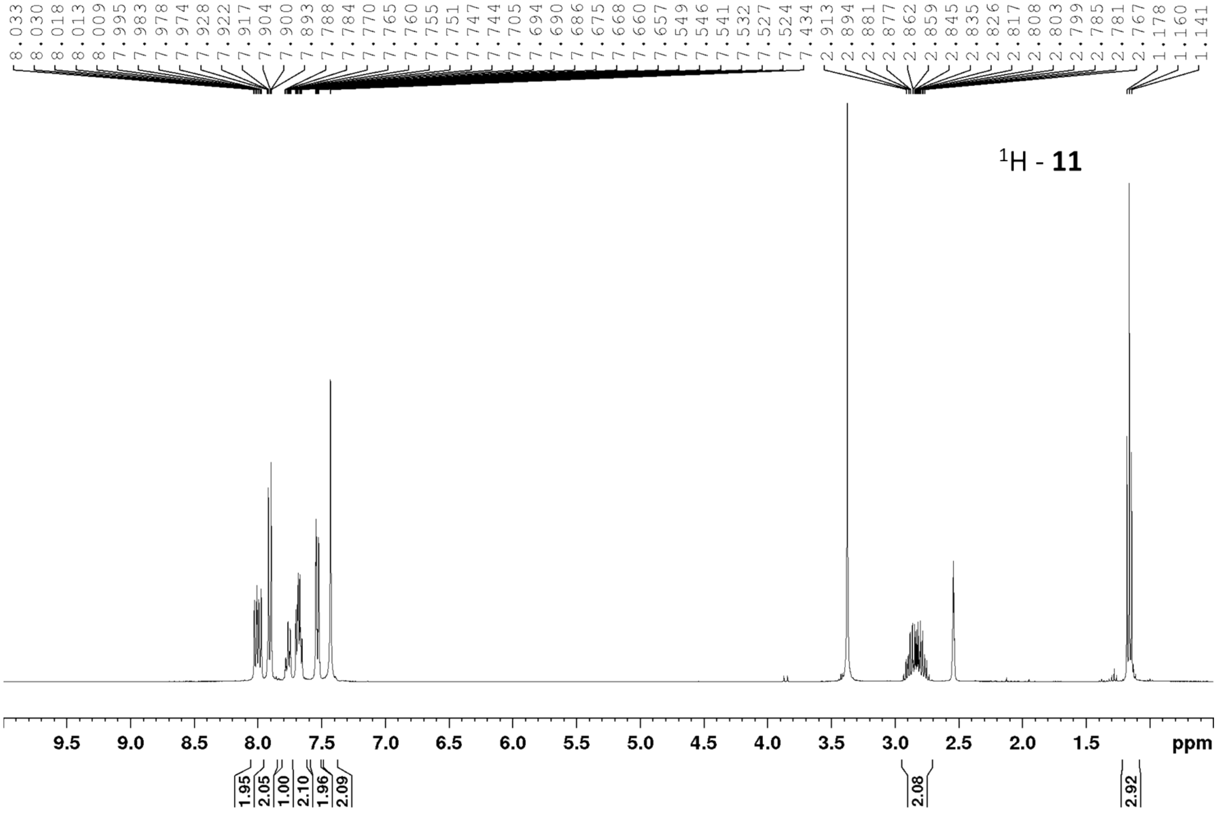
8.033 8.030 8.018 8.013 8.009 7.995 7.983 7.978 7.974 7.928 7.922 7.917 7.904 7.900 7.893 7.788 7.784 7.770 7.765 7.760 7.755 7.751 7.747 7.744 7.705 7.694 7.690 7.686 7.675 7.668 7.660 7.657 7.549 7.546 7.541 7.532 7.527 7.524 7.434 2.913 2.894 2.881 2.877 2.862 2.859 2.845 2.835 2.826 2.817 2.808 2.803 2.799 2.785 2.781 2.767 1.178 1.160 1.141
^{1}H - 11
9.5 9.0 8.5 8.0 7.5 7.0 6.5 6.0 5.5 5.0 4.5 4.0 3.5 3.0 2.5 2.0 1.5 ppm
1.95 2.05 1.00 2.10 1.96 2.09 2.08 2.92

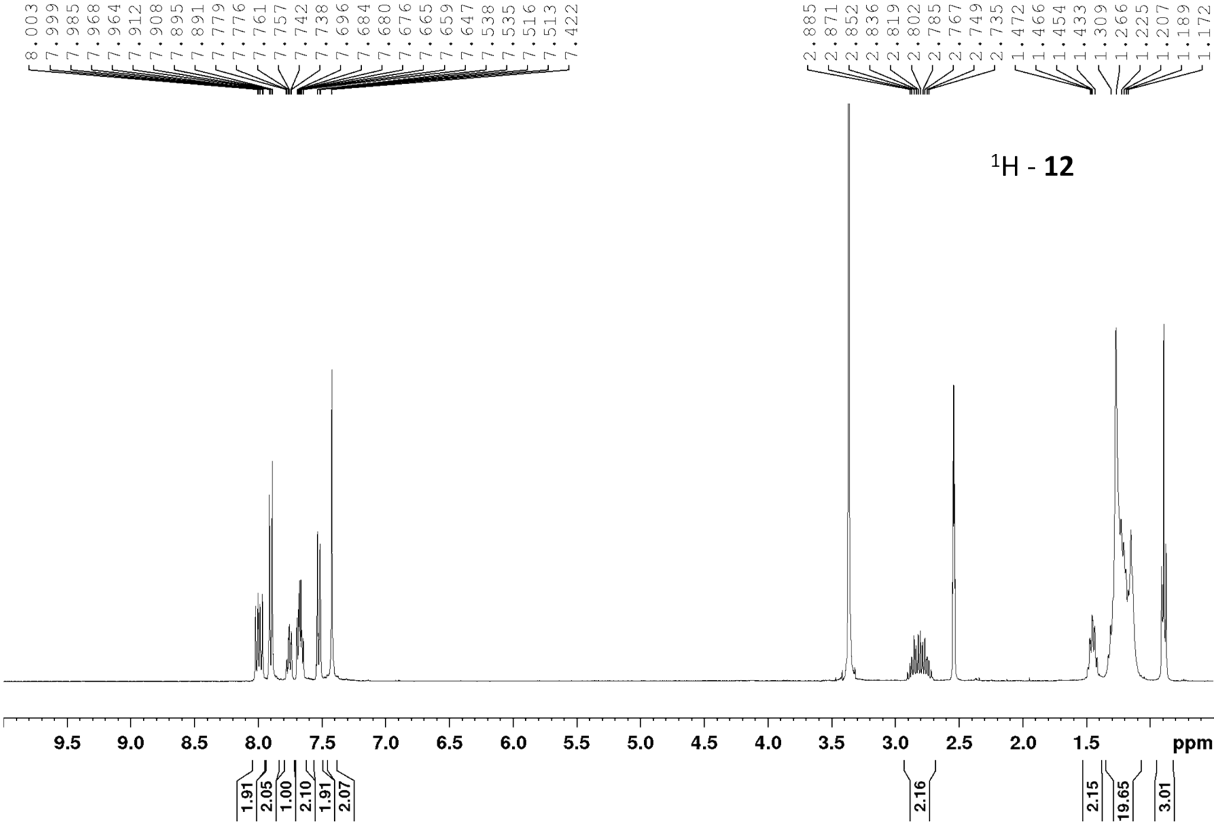
8.003 7.999 7.985 7.968 7.964 7.912 7.908 7.895 7.891 7.779 7.776 7.761 7.757 7.742 7.738 7.696 7.684 7.680 7.676 7.665 7.659 7.647 7.538 7.535 7.516 7.513 7.422
2.885 2.871 2.852 2.836 2.819 2.802 2.785 2.767 2.749 2.735 1.472 1.466 1.454 1.433 1.309 1.266 1.225 1.207 1.189 1.172
^{1}H - 12
9.5 9.0 8.5 8.0 7.5 7.0 6.5 6.0 5.5 5.0 4.5 4.0 3.5 3.0 2.5 2.0 1.5 ppm
1.91 2.05 1.00 2.10 1.91 2.07 2.16 2.15 19.65 3.01

^{1}H - **13**

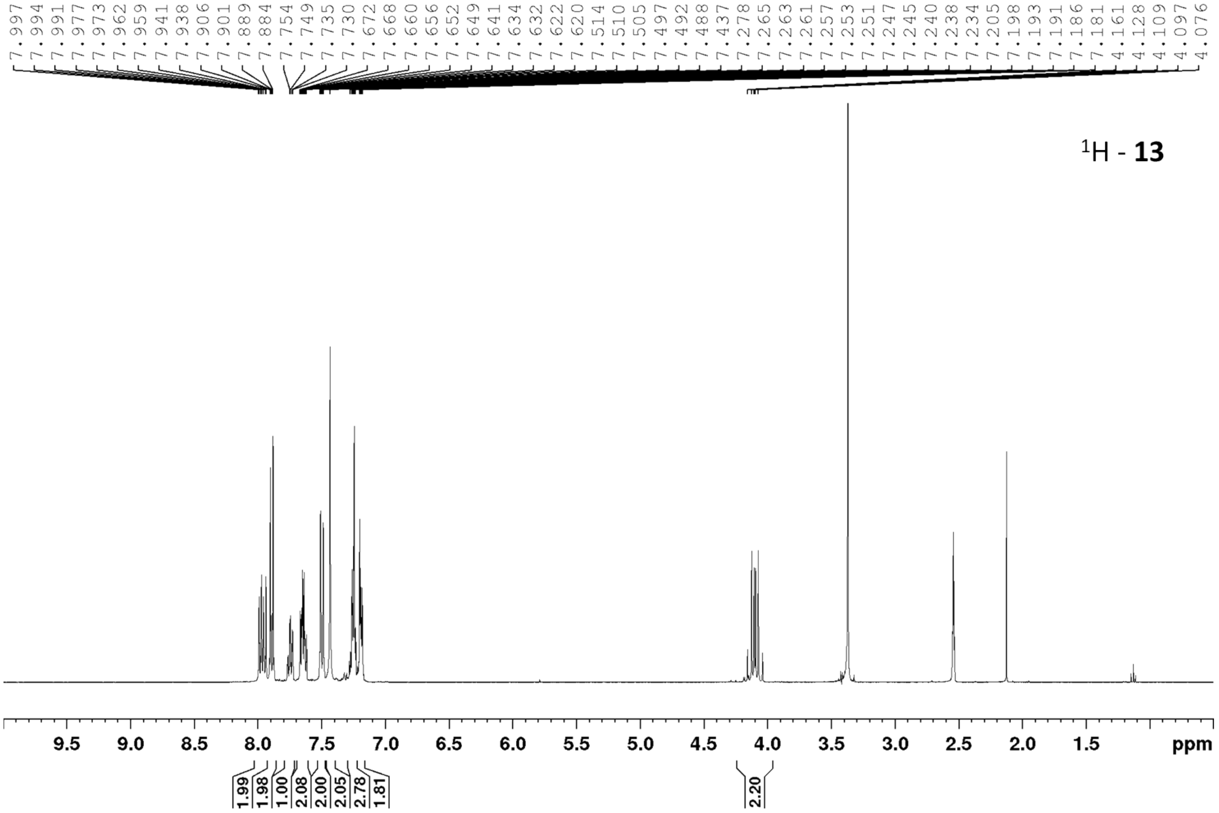
9.5 9.0 8.5 8.0 7.5 7.0 6.5 6.0 5.5 5.0 4.5 4.0 3.5 3.0 2.5 2.0 1.5 ppm
1.99 1.98 1.00 2.08 2.00 2.05 2.78 1.81
2.20

^{1}H - **14**

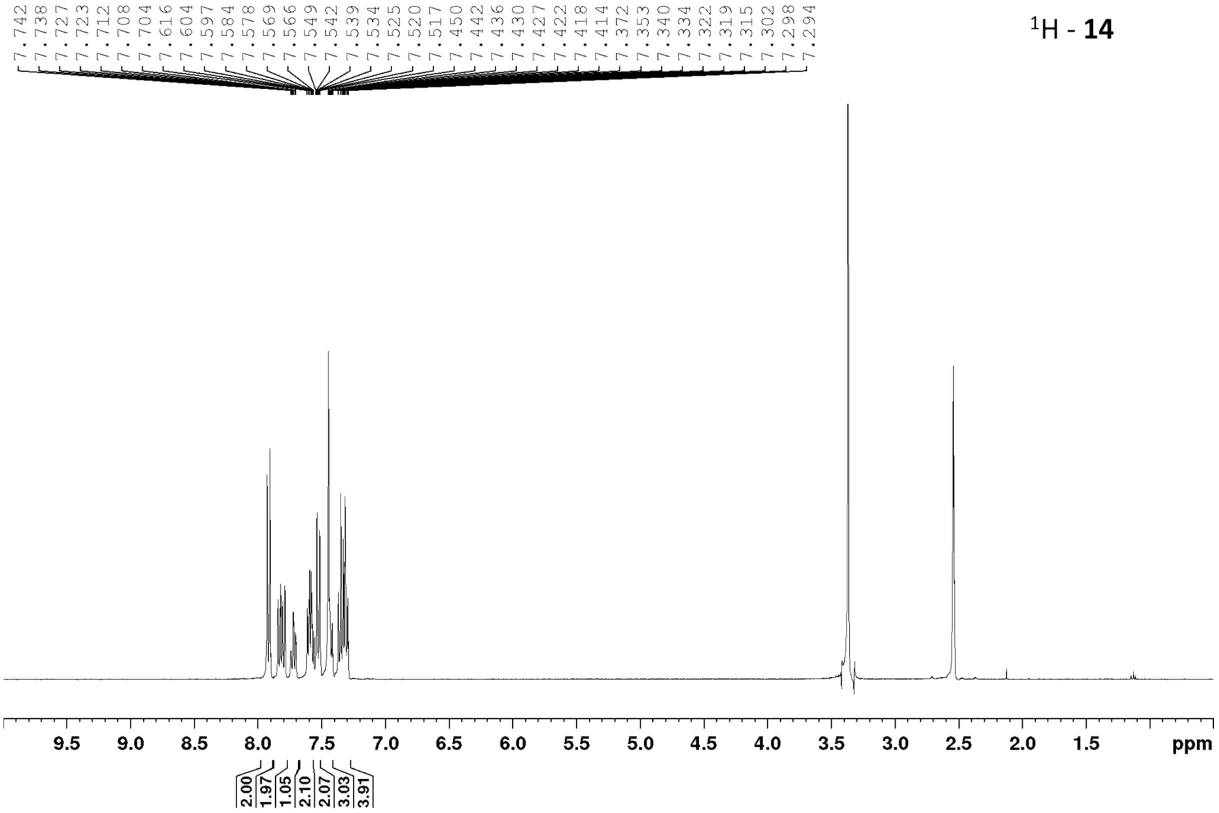
9.5 9.0 8.5 8.0 7.5 7.0 6.5 6.0 5.5 5.0 4.5 4.0 3.5 3.0 2.5 2.0 1.5 ppm
2.00 1.97 1.05 2.10 2.07 3.03 3.91

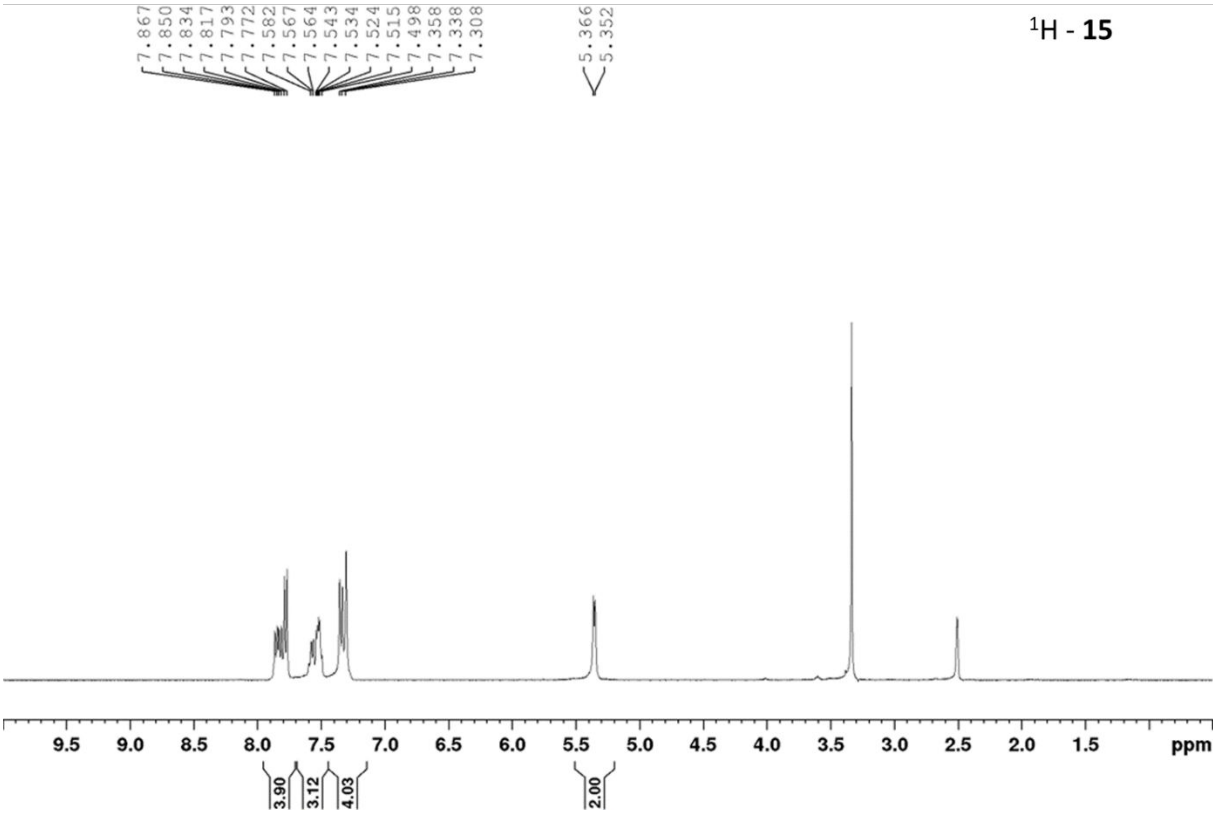
7.867
7.850
7.834
7.817
7.793
7.772
7.582
7.567
7.564
7.543
7.534
7.524
7.515
7.498
7.358
7.338
7.308
5.366
5.352
9.5 9.0 8.5 8.0 7.5 7.0 6.5 6.0 5.5 5.0 4.5 4.0 3.5 3.0 2.5 2.0 1.5 ppm
3.90
3.12
4.03
2.00

^1H - **15**

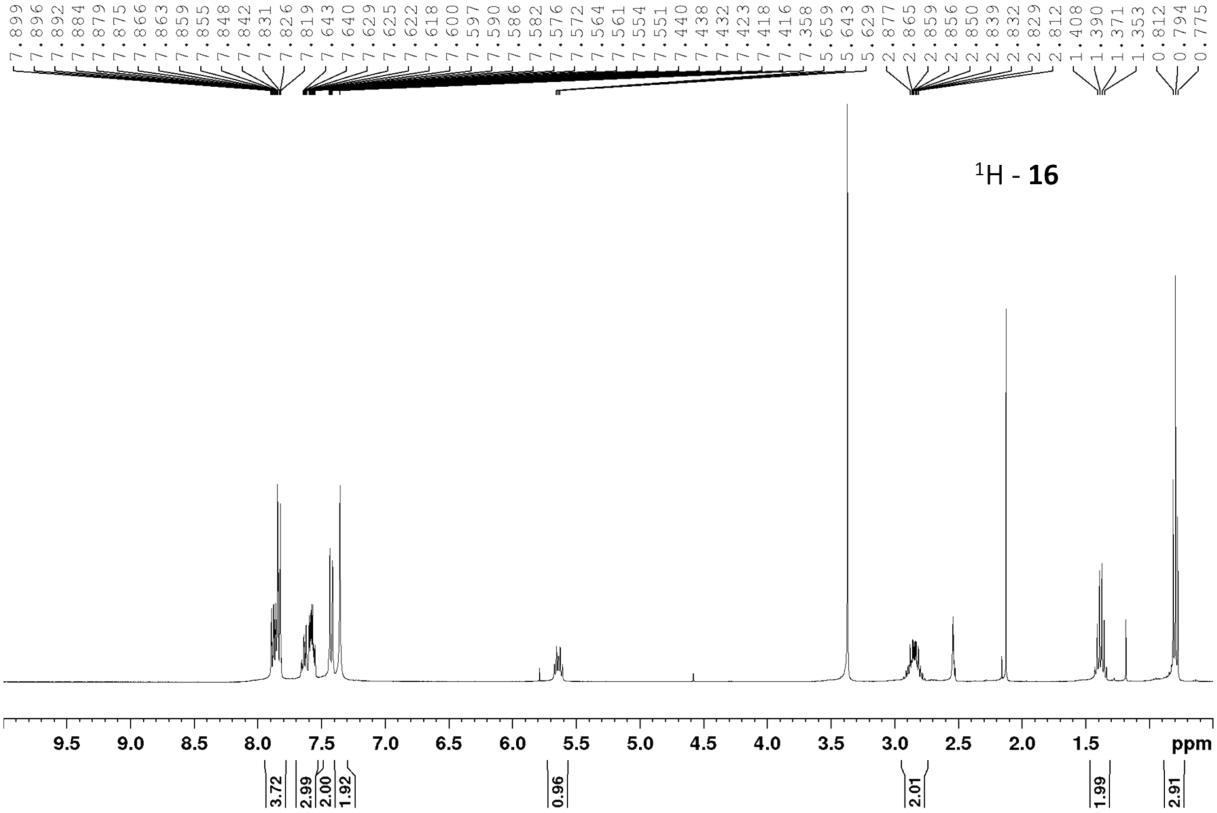
7.899
7.896
7.892
7.884
7.879
7.875
7.866
7.863
7.859
7.855
7.848
7.842
7.831
7.826
7.819
7.643
7.640
7.629
7.625
7.622
7.618
7.600
7.597
7.590
7.586
7.582
7.576
7.572
7.564
7.561
7.554
7.551
7.440
7.438
7.432
7.423
7.418
7.416
7.358
5.659
5.643
5.629
2.877
2.865
2.859
2.856
2.850
2.839
2.832
2.829
2.812
1.408
1.390
1.371
1.353
0.812
0.794
0.775
9.5 9.0 8.5 8.0 7.5 7.0 6.5 6.0 5.5 5.0 4.5 4.0 3.5 3.0 2.5 2.0 1.5 ppm
3.72
2.99
2.00
1.92
0.96
2.01
1.99
2.91

^1H - **16**

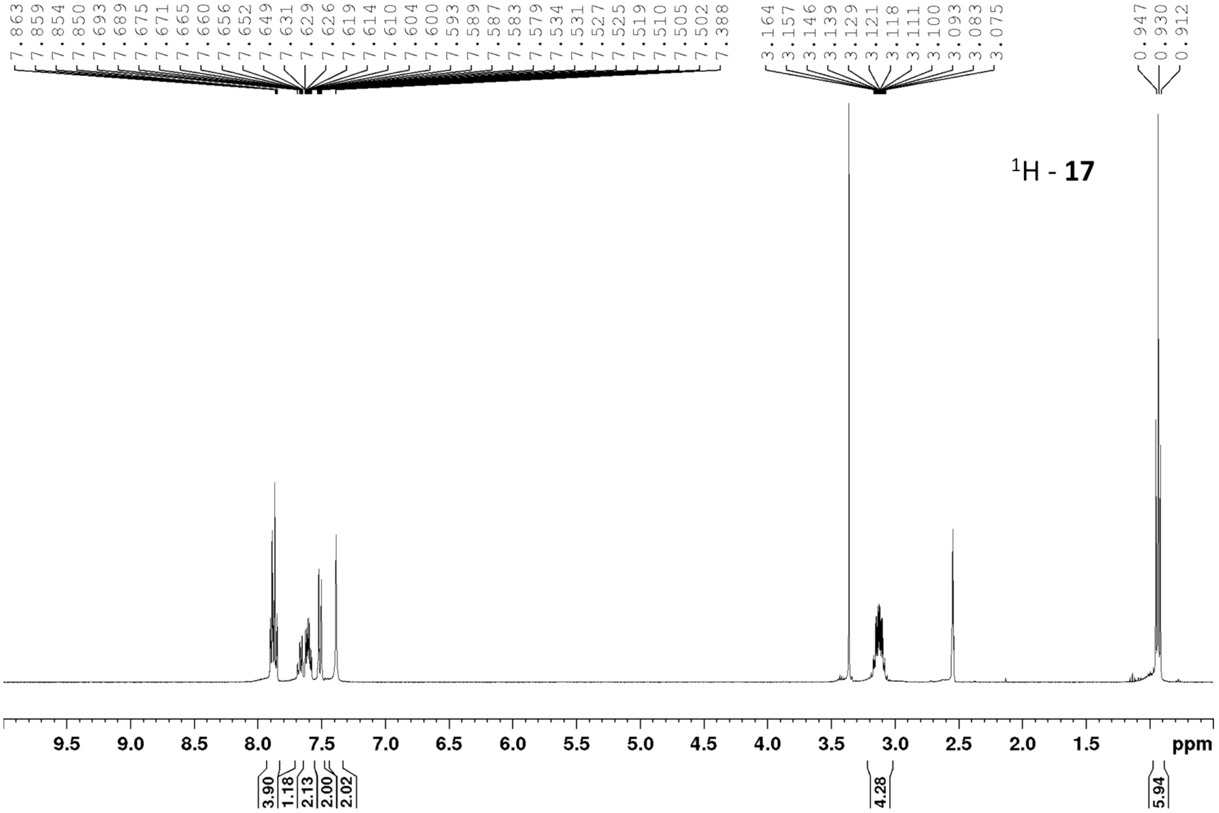
7.863 7.859 7.854 7.850 7.693 7.689 7.675 7.671 7.665 7.660 7.656 7.652 7.649 7.631 7.629 7.626 7.619 7.614 7.610 7.604 7.600 7.593 7.589 7.587 7.583 7.579 7.534 7.531 7.527 7.525 7.519 7.510 7.505 7.502 7.388
3.164 3.157 3.146 3.139 3.129 3.121 3.118 3.111 3.100 3.093 3.083 3.075
0.947 0.930 0.912
^{1}H - **17**
9.5 9.0 8.5 8.0 7.5 7.0 6.5 6.0 5.5 5.0 4.5 4.0 3.5 3.0 2.5 2.0 1.5 ppm
3.90 1.18 2.13 2.00 2.02
4.28
5.94

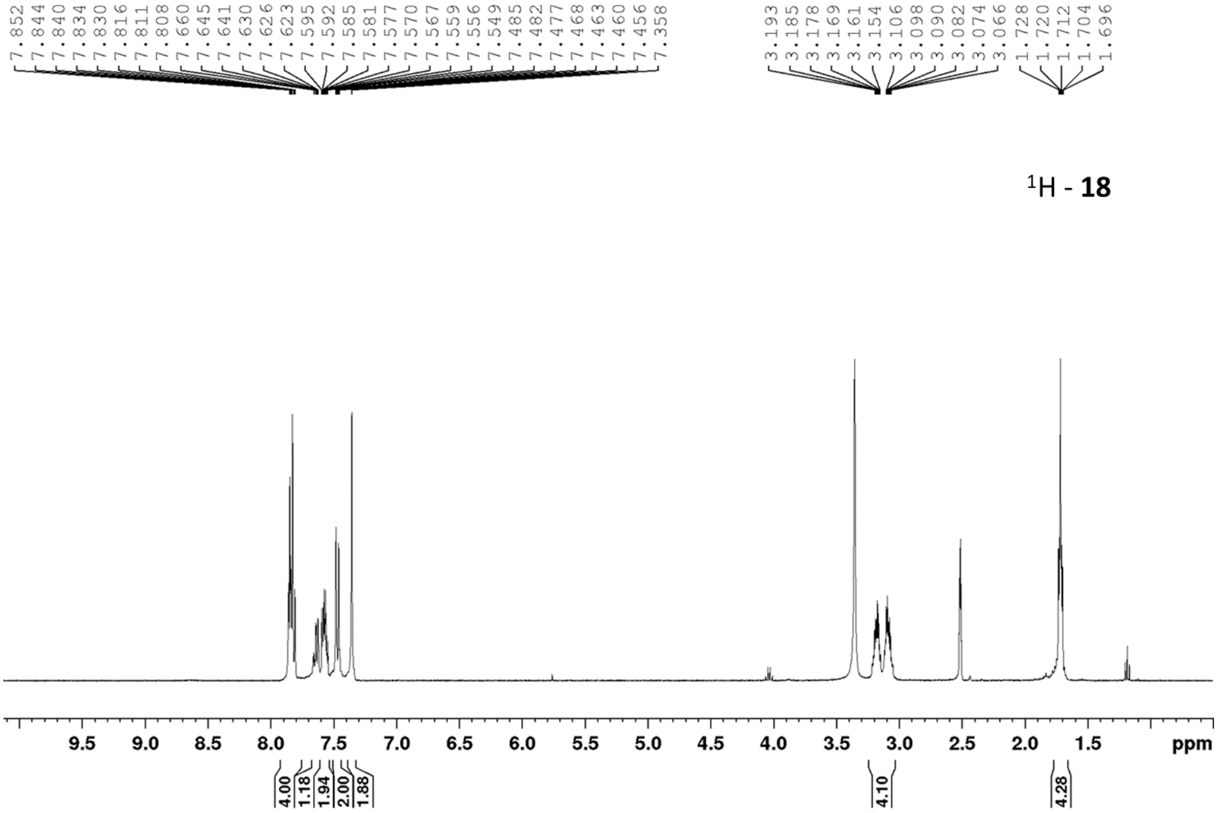
7.852 7.844 7.840 7.834 7.830 7.816 7.811 7.808 7.660 7.645 7.641 7.630 7.626 7.623 7.595 7.592 7.585 7.581 7.577 7.570 7.567 7.559 7.556 7.549 7.485 7.482 7.477 7.468 7.463 7.460 7.456 7.358
3.193 3.185 3.178 3.169 3.161 3.154 3.106 3.098 3.090 3.082 3.074 3.066
1.728 1.720 1.712 1.704 1.696
^{1}H - **18**
9.5 9.0 8.5 8.0 7.5 7.0 6.5 6.0 5.5 5.0 4.5 4.0 3.5 3.0 2.5 2.0 1.5 ppm
4.00 1.18 1.94 2.00 1.88
4.10
4.28

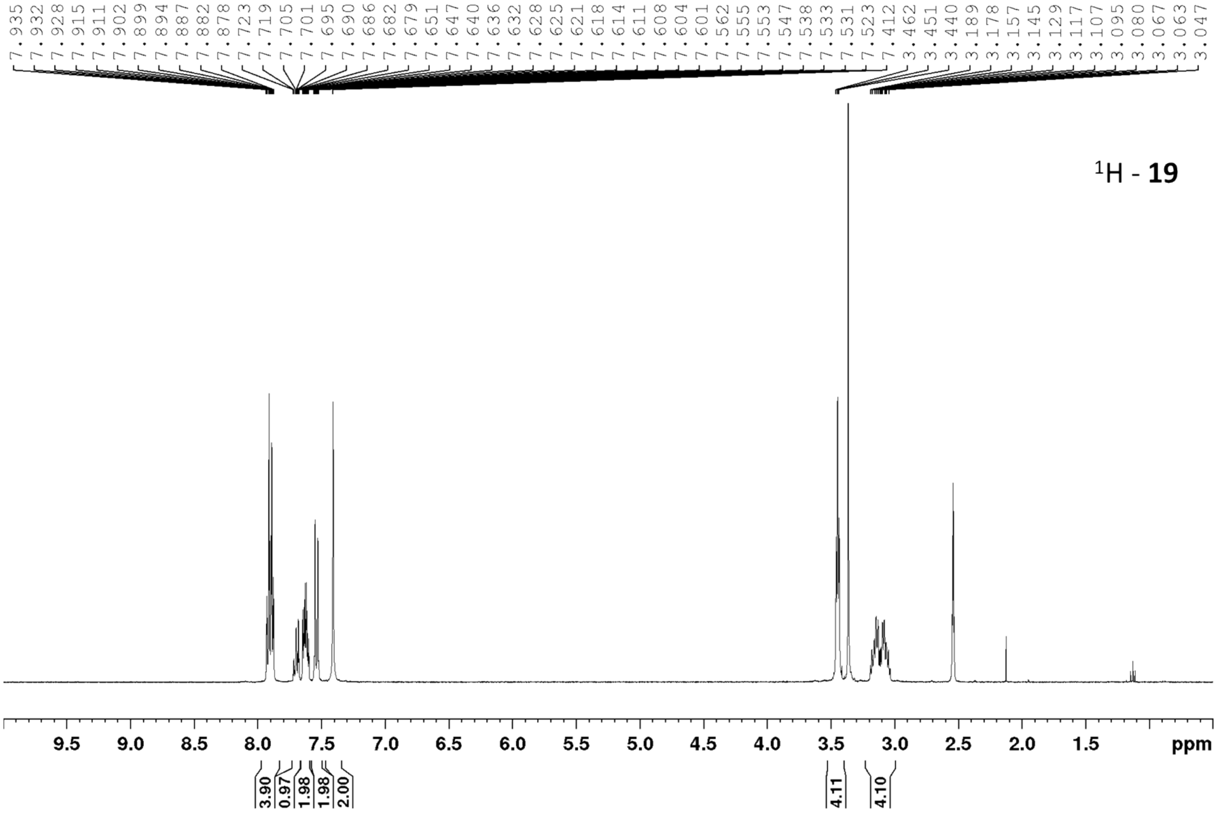
1H - 19
ppm
3.90
0.97
1.98
1.98
2.00
4.11
4.10

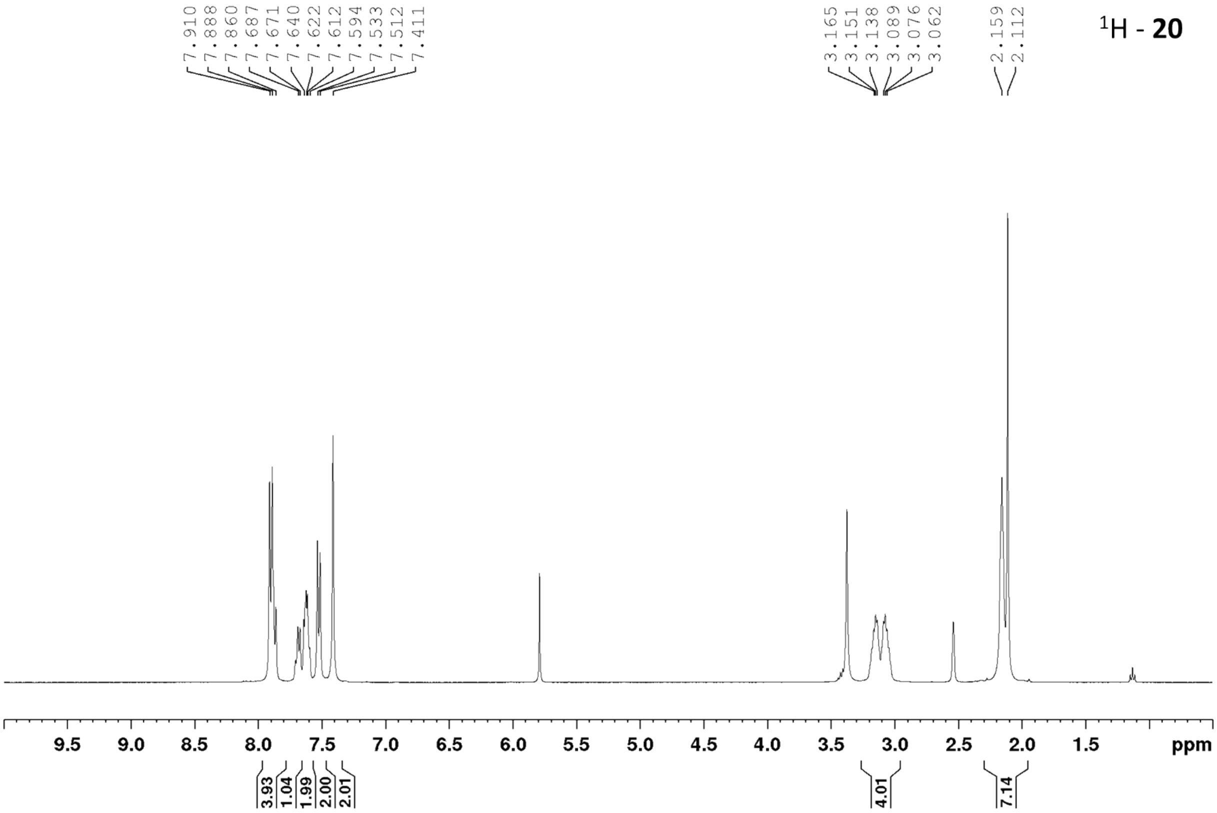
1H - 20
ppm
3.93
1.04
1.99
2.00
2.01
4.01
7.14

^{1}H - **21**

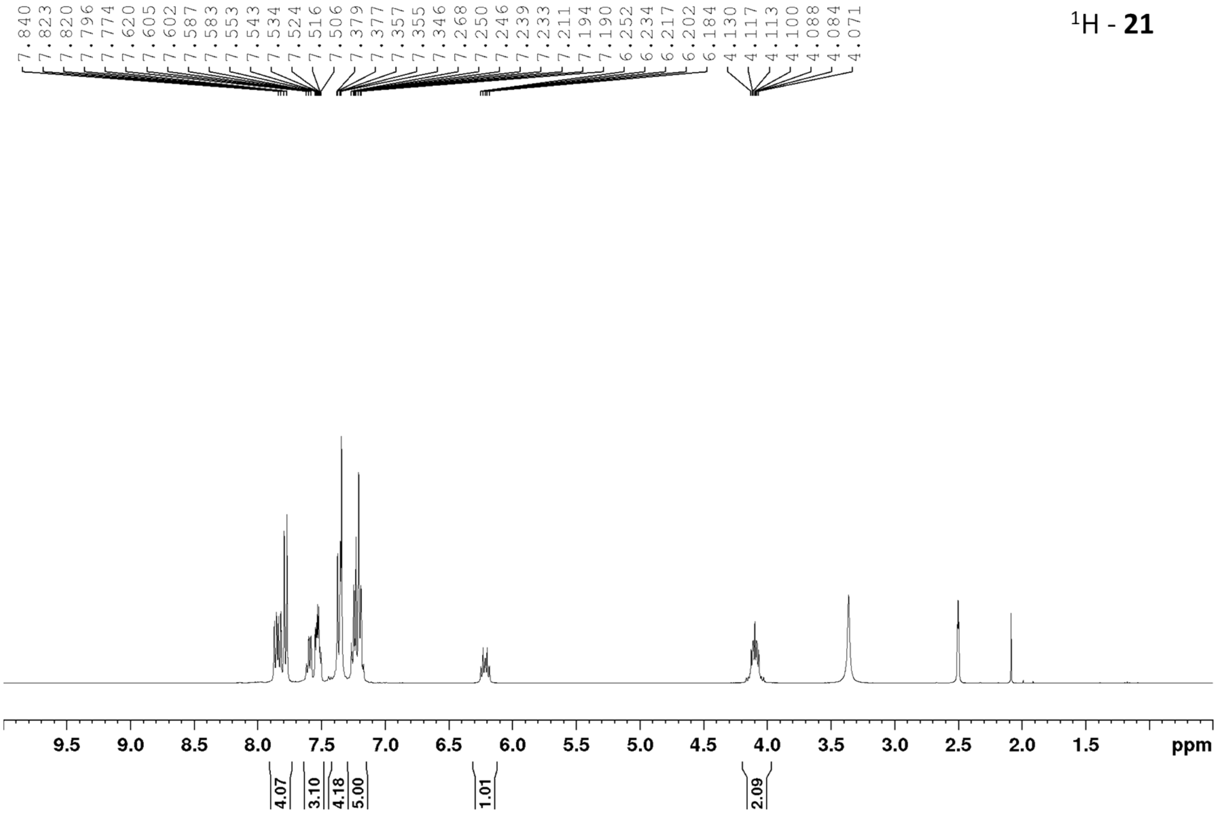

^{1}H - **22**

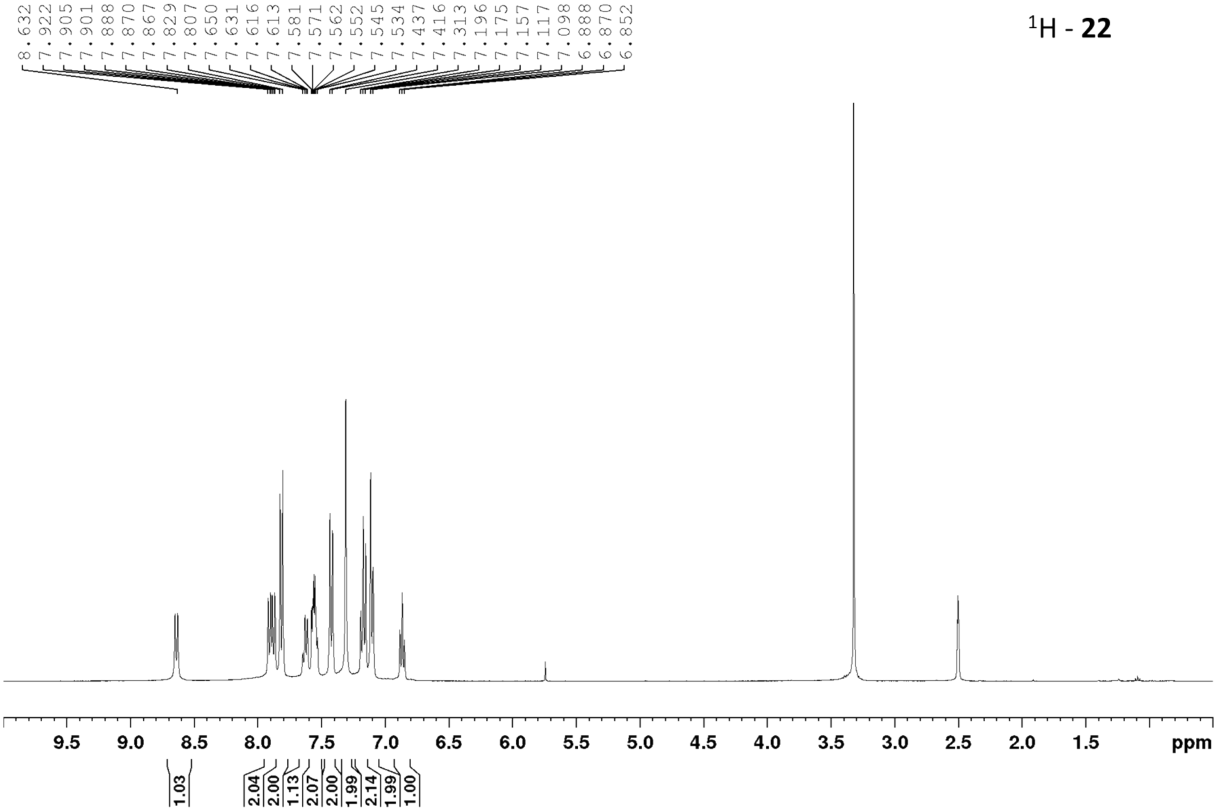

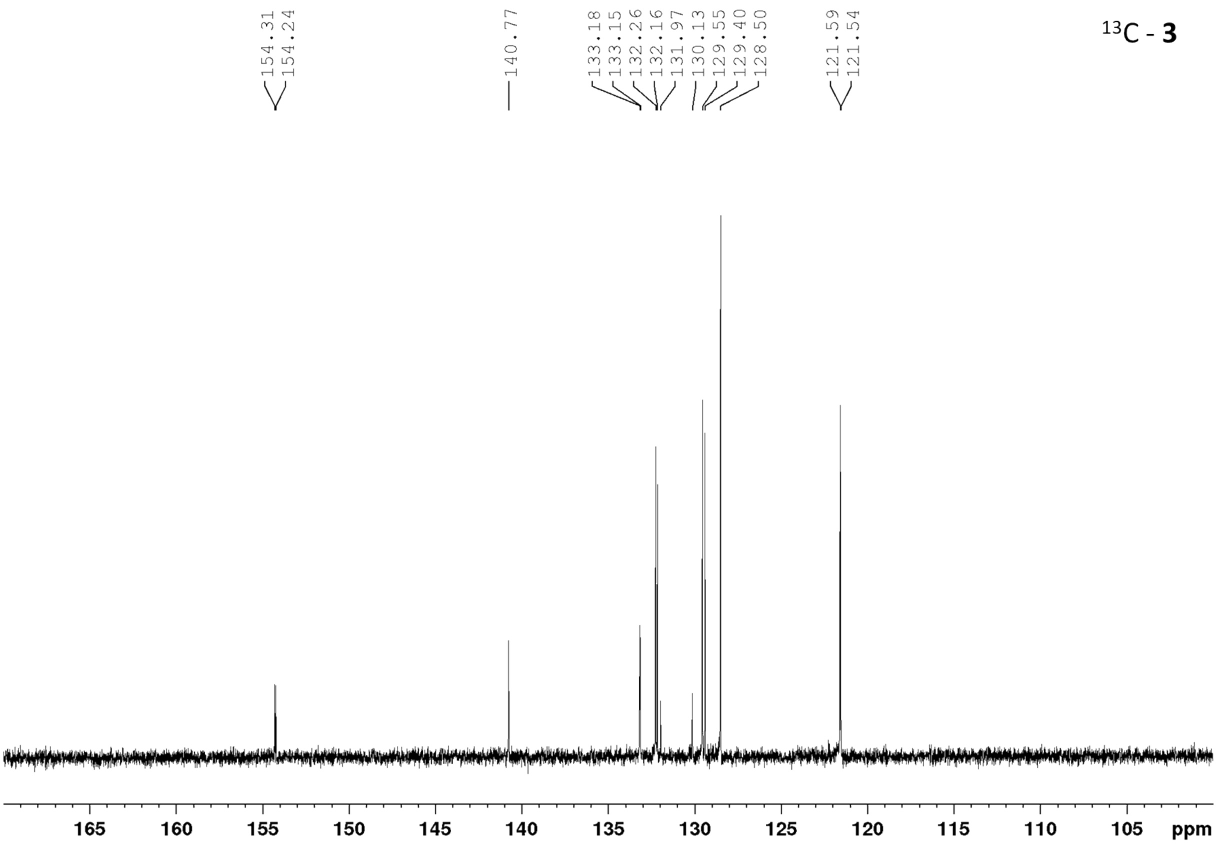
154.31
154.24
140.77
133.18
133.15
132.26
132.16
131.97
130.13
129.55
129.40
128.50
121.59
121.54
^{13}C - 3
165 160 155 150 145 140 135 130 125 120 115 110 105 ppm

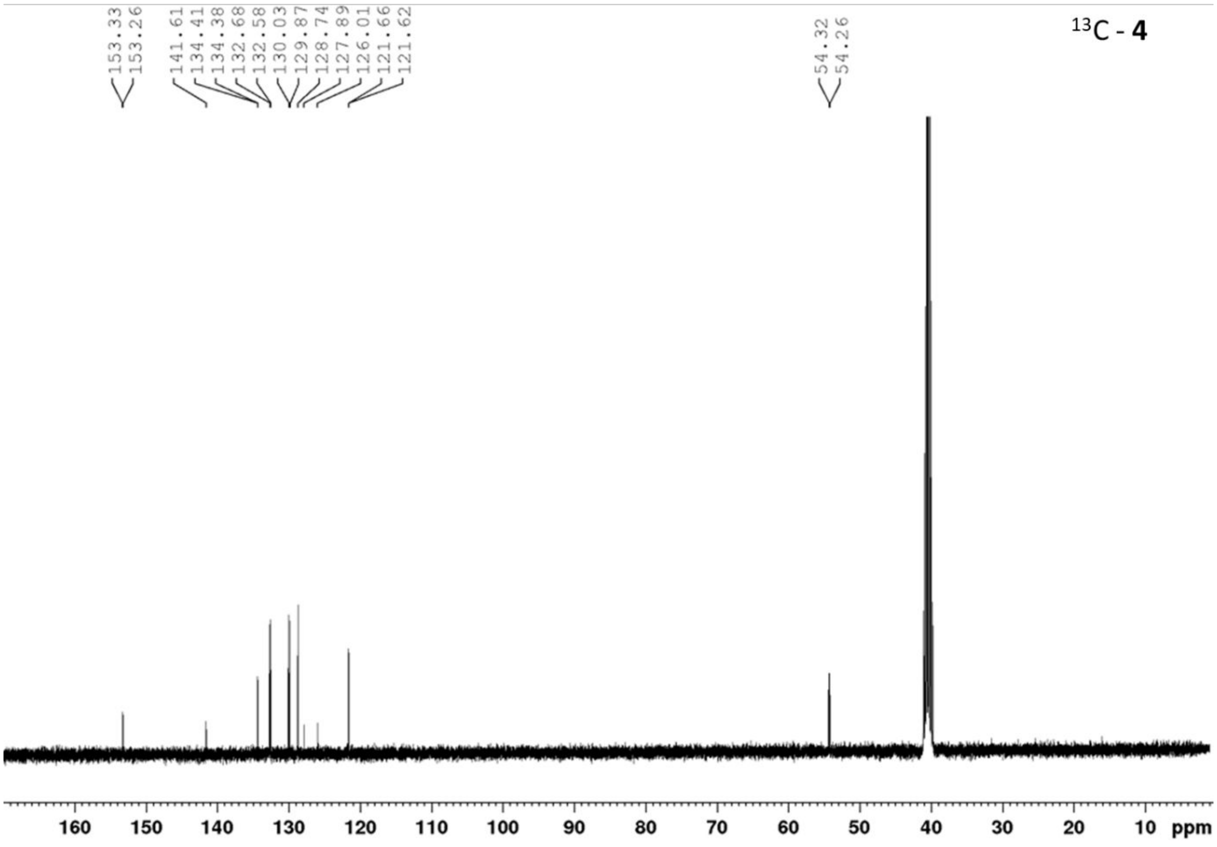
153.33
153.26
141.61
134.41
134.38
132.68
132.58
130.03
129.87
128.74
127.89
126.01
121.66
121.62
54.32
54.26
^{13}C - 4
160 150 140 130 120 110 100 90 80 70 60 50 40 30 20 10 ppm

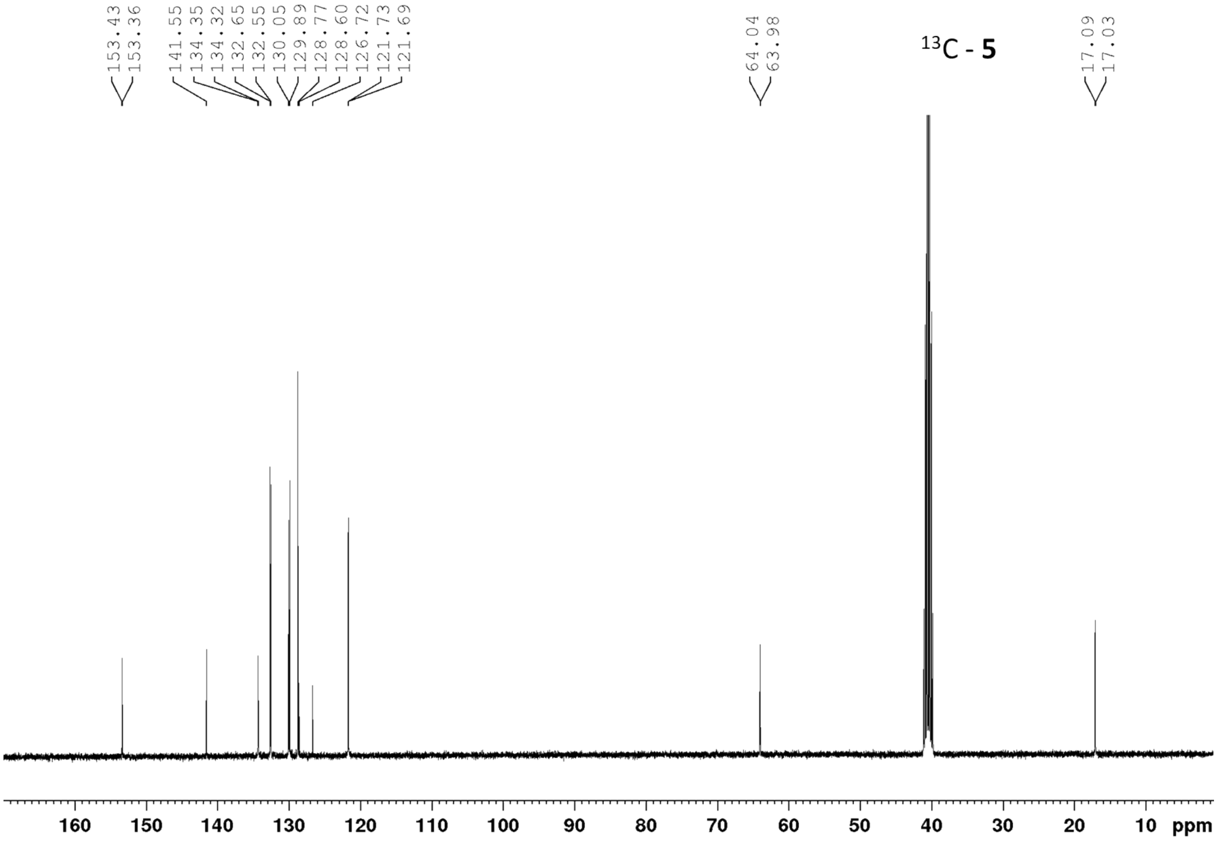
153.43
153.36
141.55
134.35
134.32
132.65
132.55
130.05
129.89
128.77
128.60
126.72
121.73
121.69
64.04
63.98
^{13}C - 5
17.09
17.03
160 150 140 130 120 110 100 90 80 70 60 50 40 30 20 10 ppm

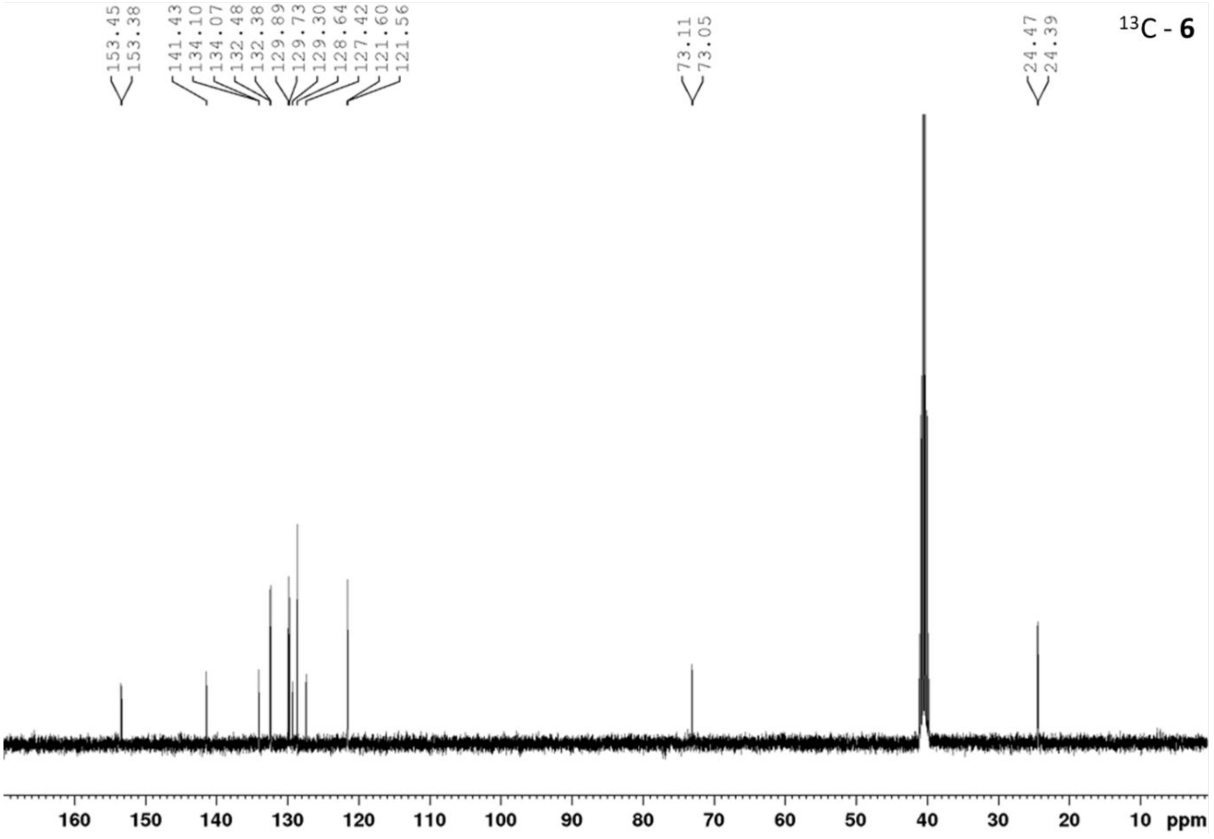
153.45
153.38
141.43
134.10
134.07
132.48
132.38
129.89
129.73
129.30
128.64
127.42
121.60
121.56
73.11
73.05
24.47
24.39
^{13}C - 6
160 150 140 130 120 110 100 90 80 70 60 50 40 30 20 10 ppm

^{13}C - **7**

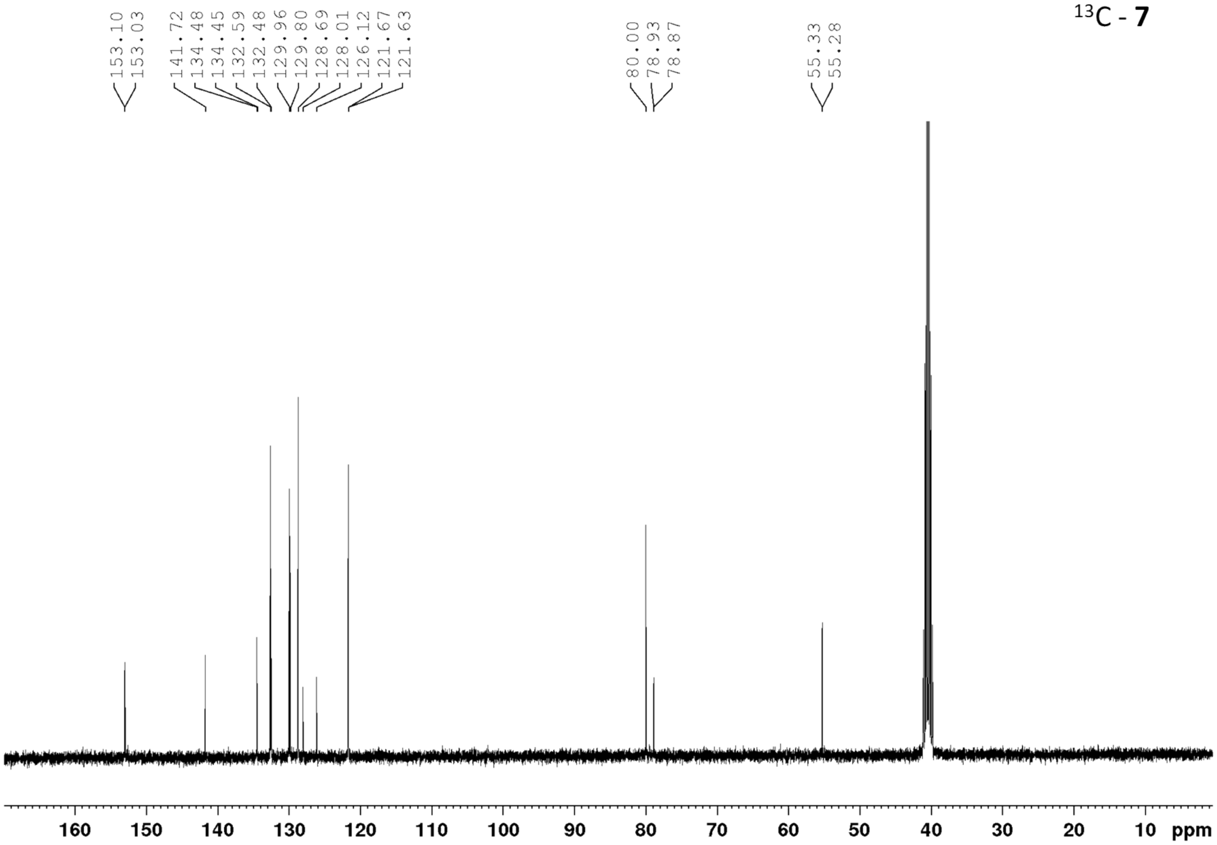
153.10
153.03
141.72
134.48
134.45
132.59
132.48
129.96
129.80
128.69
128.01
126.12
121.67
121.63
80.00
78.93
78.87
55.33
55.28
160
150
140
130
120
110
100
90
80
70
60
50
40
30
20
10
ppm

^{13}C - **8**

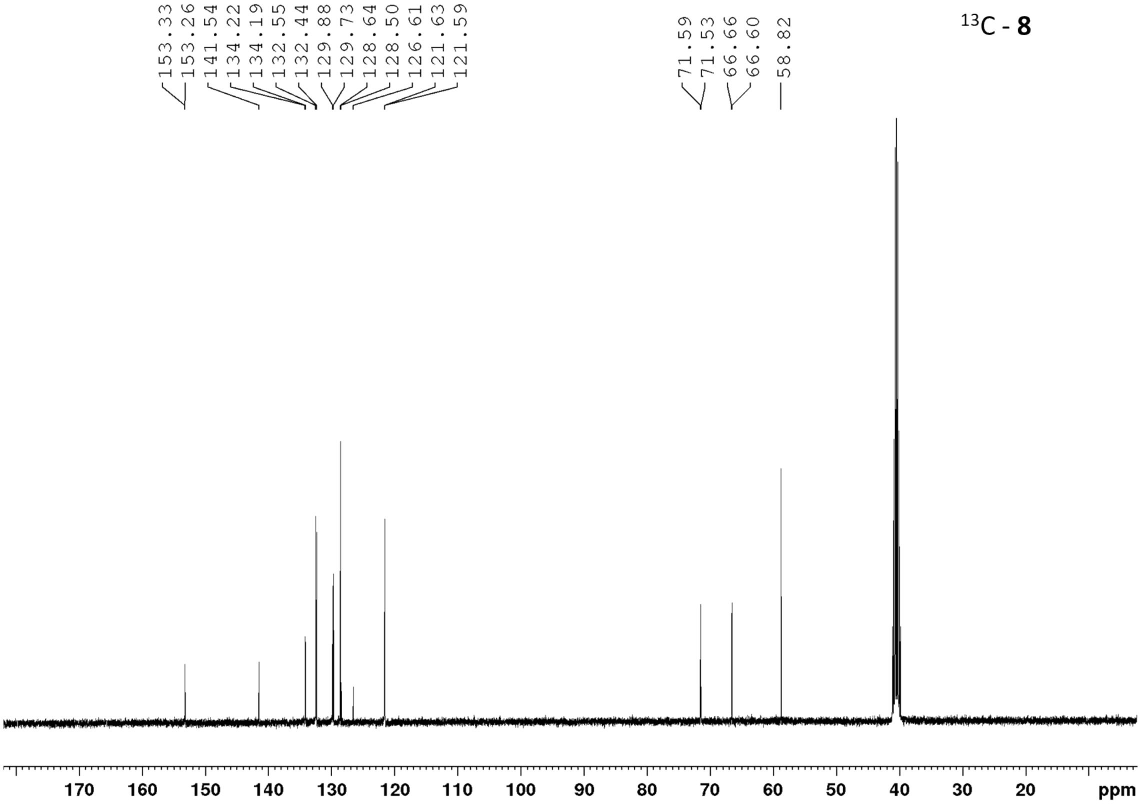
153.33
153.26
141.54
134.22
134.19
132.55
132.44
129.88
129.73
128.64
128.50
126.61
121.63
121.59
71.59
71.53
66.66
66.60
58.82
170
160
150
140
130
120
110
100
90
80
70
60
50
40
30
20
ppm

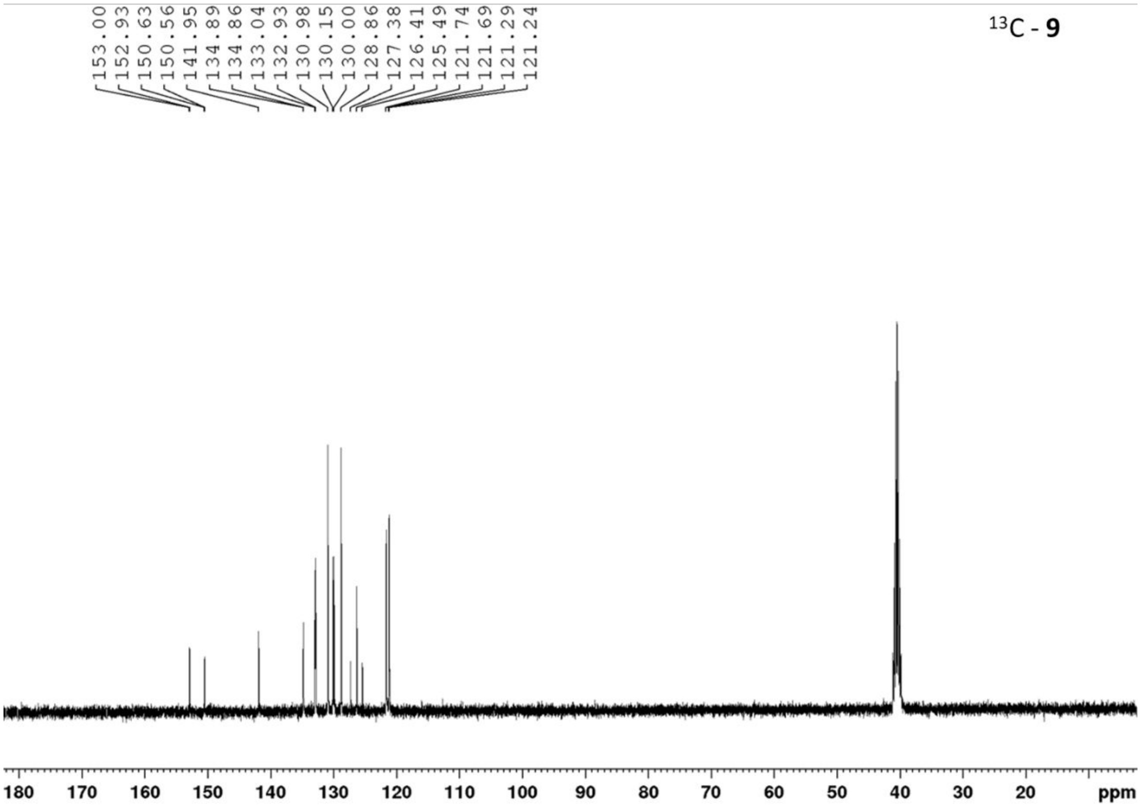
153.00
152.93
150.63
150.56
141.95
134.89
134.86
133.04
132.93
130.98
130.15
130.00
128.86
127.38
126.41
125.49
121.74
121.69
121.29
121.24
180 170 160 150 140 130 120 110 100 90 80 70 60 50 40 30 20 ppm

^{13}C - **9**

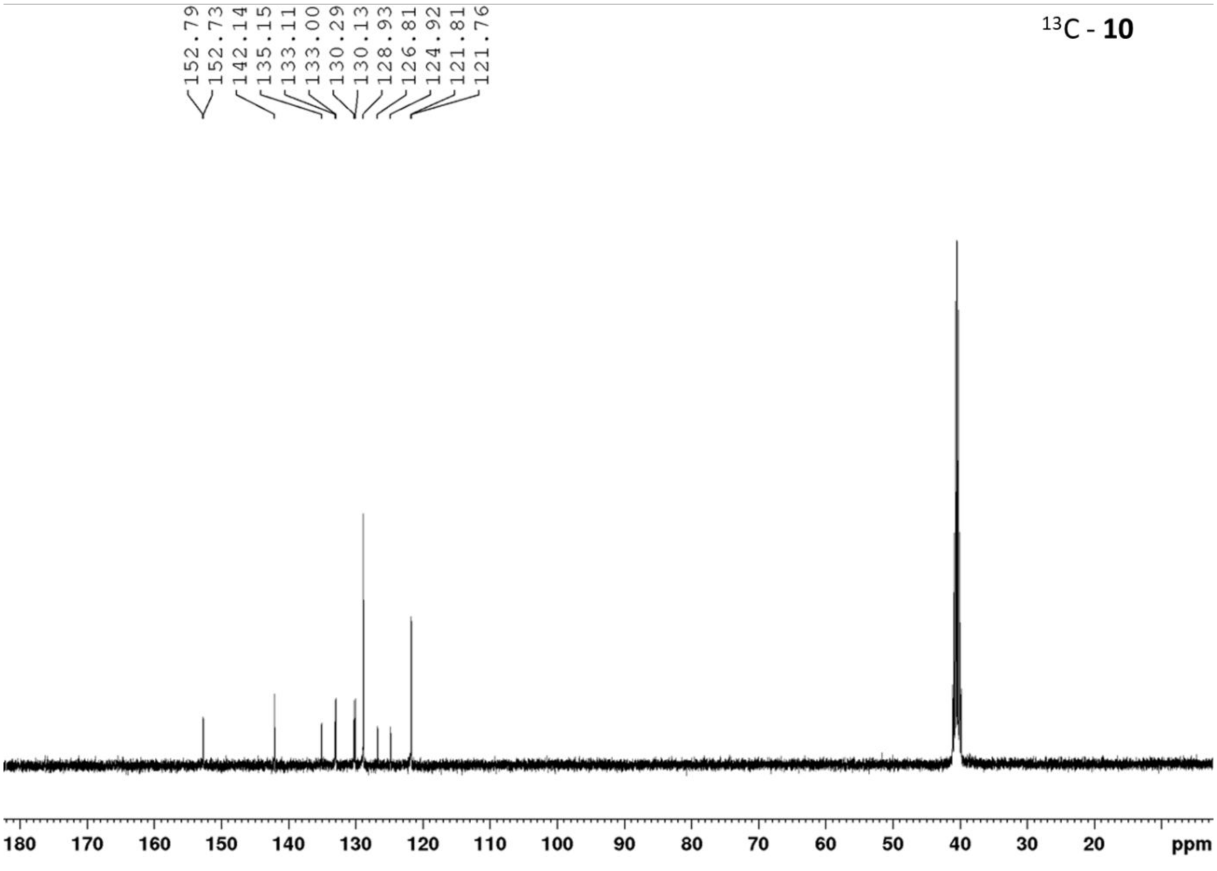
152.79
152.73
142.14
135.15
133.11
133.00
130.29
130.13
128.93
126.81
124.92
121.81
121.76
180 170 160 150 140 130 120 110 100 90 80 70 60 50 40 30 20 ppm

^{13}C - **10**

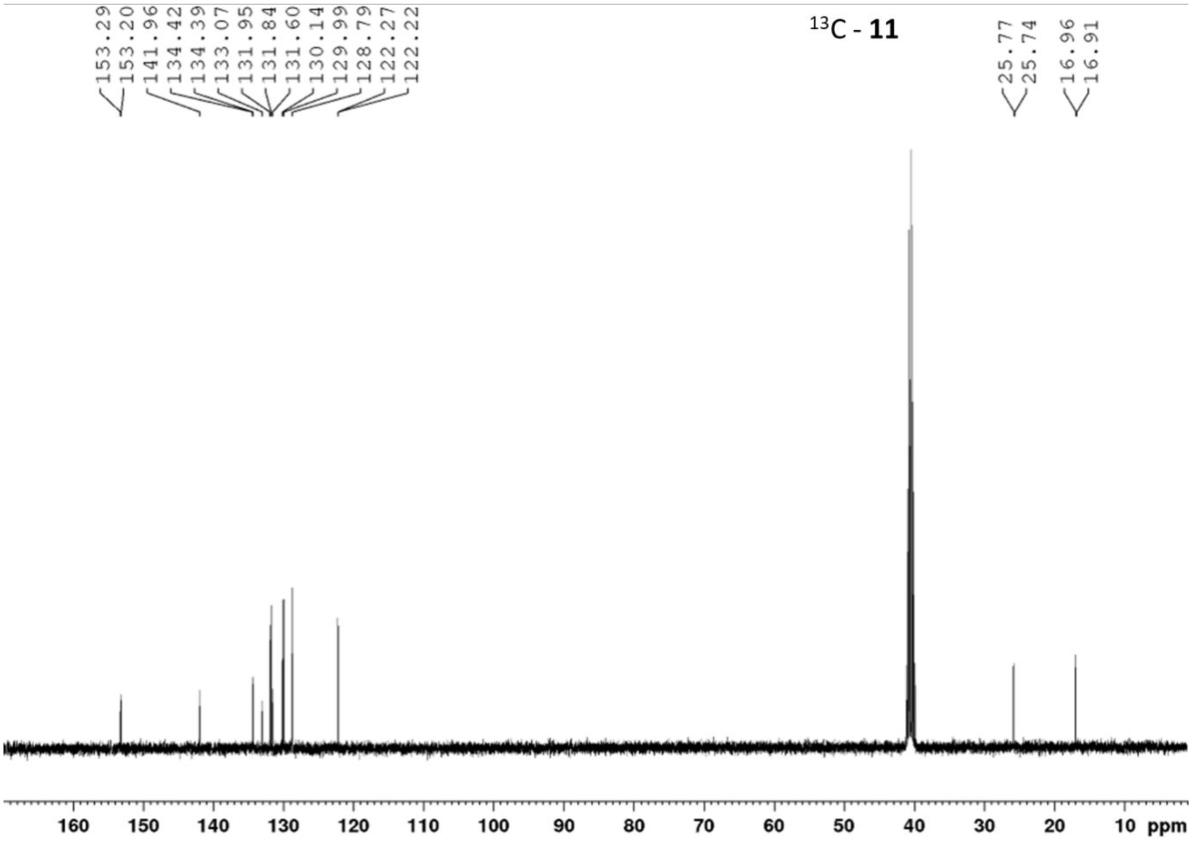
153.29
153.20
141.96
134.42
134.39
133.07
131.95
131.84
131.60
130.14
129.99
128.79
122.27
122.22
^{13}C - **11**
25.77
25.74
16.96
16.91
160 150 140 130 120 110 100 90 80 70 60 50 40 30 20 10 ppm

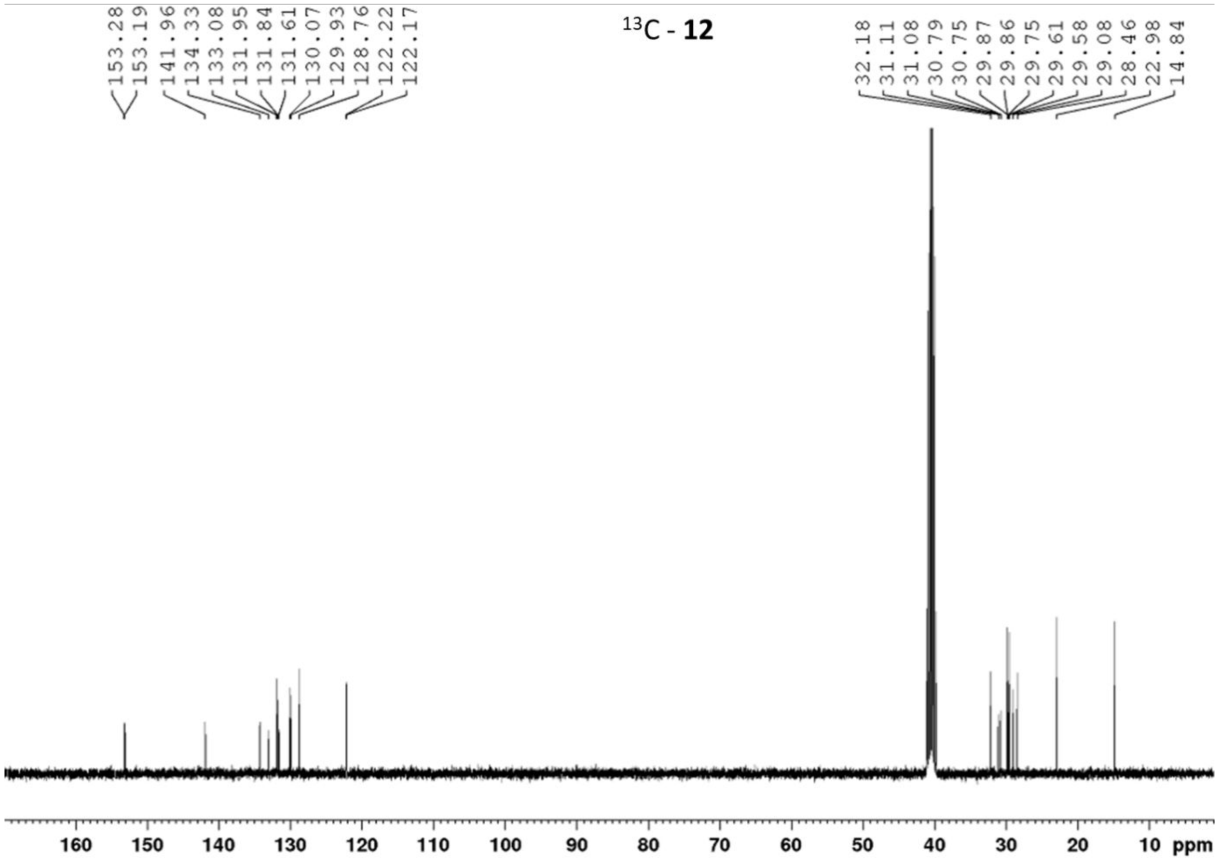
153.28
153.19
141.96
134.33
133.08
131.95
131.84
131.61
130.07
129.93
128.76
122.22
122.17
^{13}C - **12**
32.18
31.11
31.08
30.79
30.75
29.87
29.86
29.75
29.61
29.58
29.08
28.46
22.98
14.84
160 150 140 130 120 110 100 90 80 70 60 50 40 30 20 10 ppm

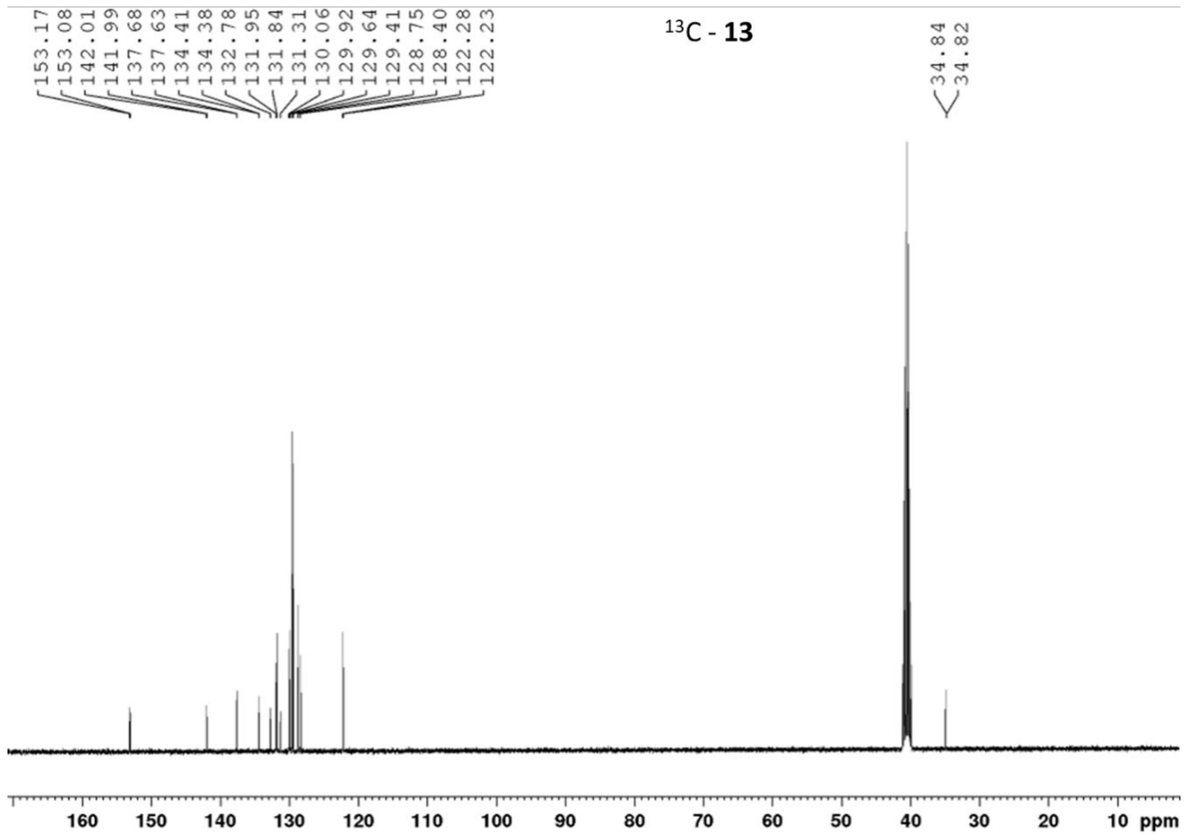

¹³C - 13
153.17
153.08
142.01
141.99
137.68
137.63
134.41
134.38
132.78
131.95
131.84
131.31
130.06
129.92
129.64
129.41
128.75
128.40
122.28
122.23
34.84
34.82
160 150 140 130 120 110 100 90 80 70 60 50 40 30 20 10 ppm

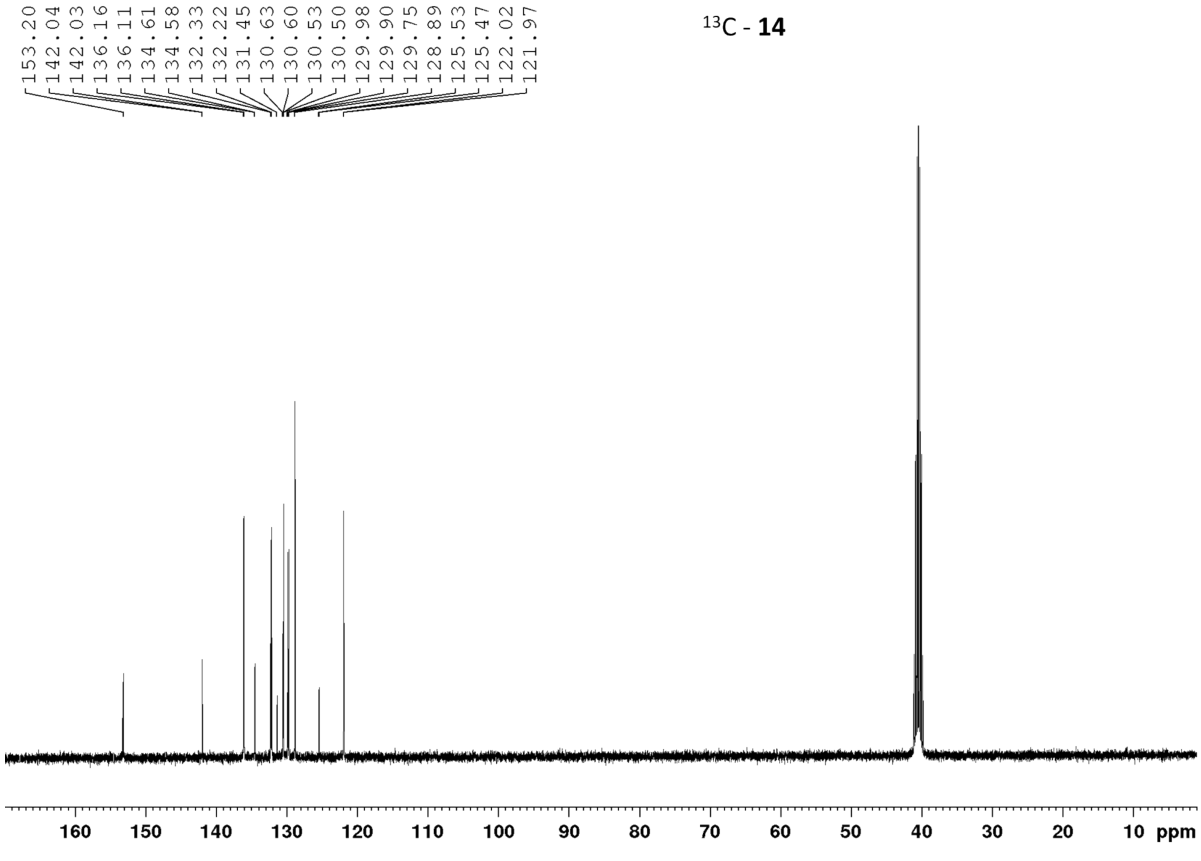

¹³C - 14
153.20
142.04
142.03
136.16
136.11
134.61
134.58
132.33
132.22
131.45
130.63
130.60
130.53
130.50
129.98
129.90
129.75
128.89
125.53
125.47
122.02
121.97
160 150 140 130 120 110 100 90 80 70 60 50 40 30 20 10 ppm

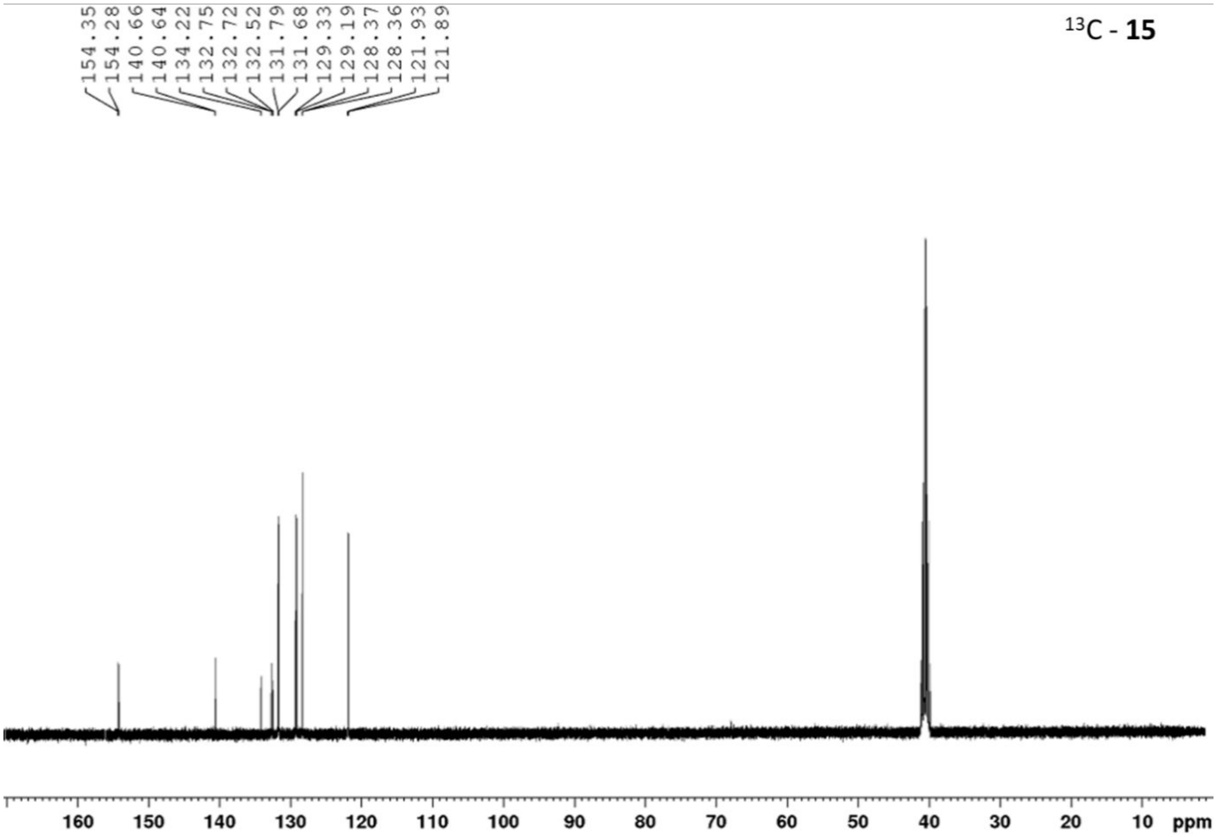
154.35
154.28
140.66
140.64
134.22
132.75
132.72
132.52
131.79
131.68
129.33
129.19
128.37
128.36
121.93
121.89
^{13}C - **15**
160 150 140 130 120 110 100 90 80 70 60 50 40 30 20 10 ppm

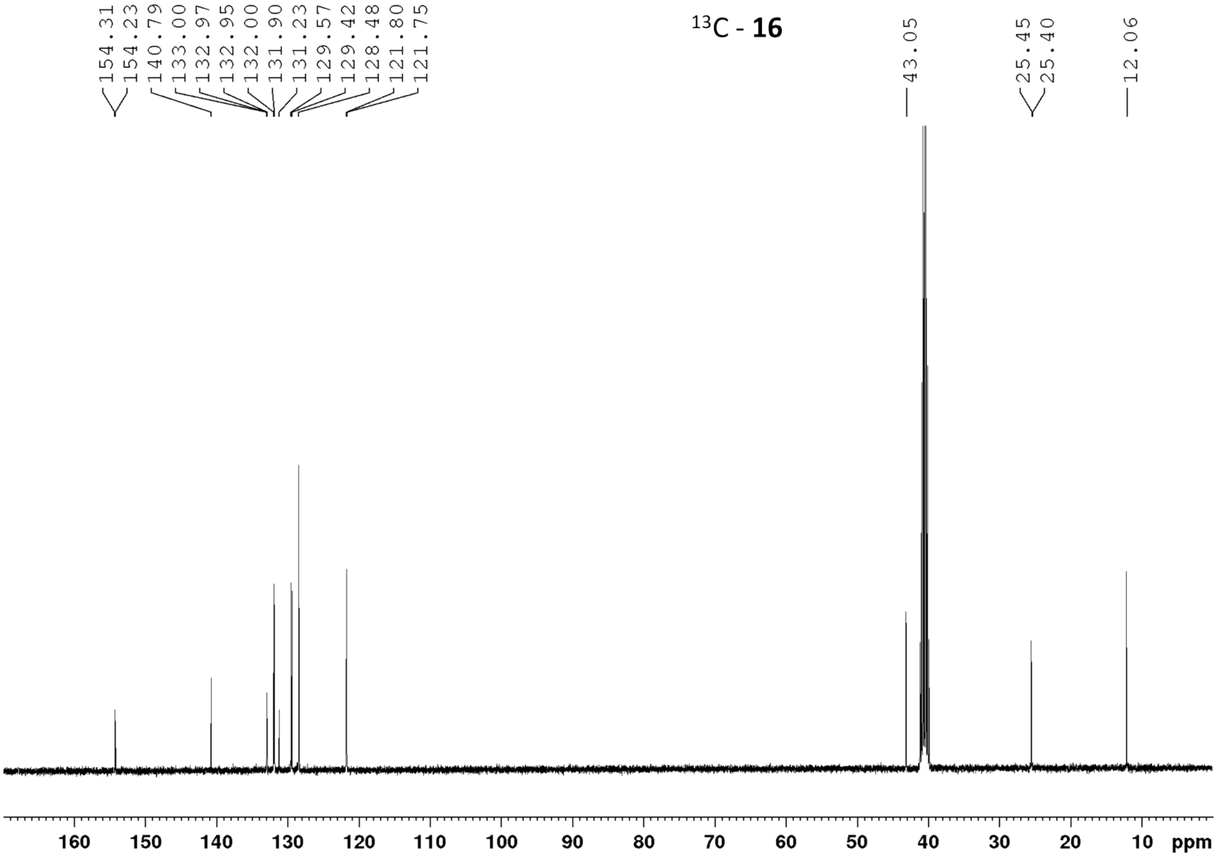
154.31
154.23
140.79
133.00
132.97
132.95
132.00
131.90
131.23
129.57
129.42
128.48
121.80
121.75
^{13}C - **16**
43.05
25.45
25.40
12.06
160 150 140 130 120 110 100 90 80 70 60 50 40 30 20 10 ppm

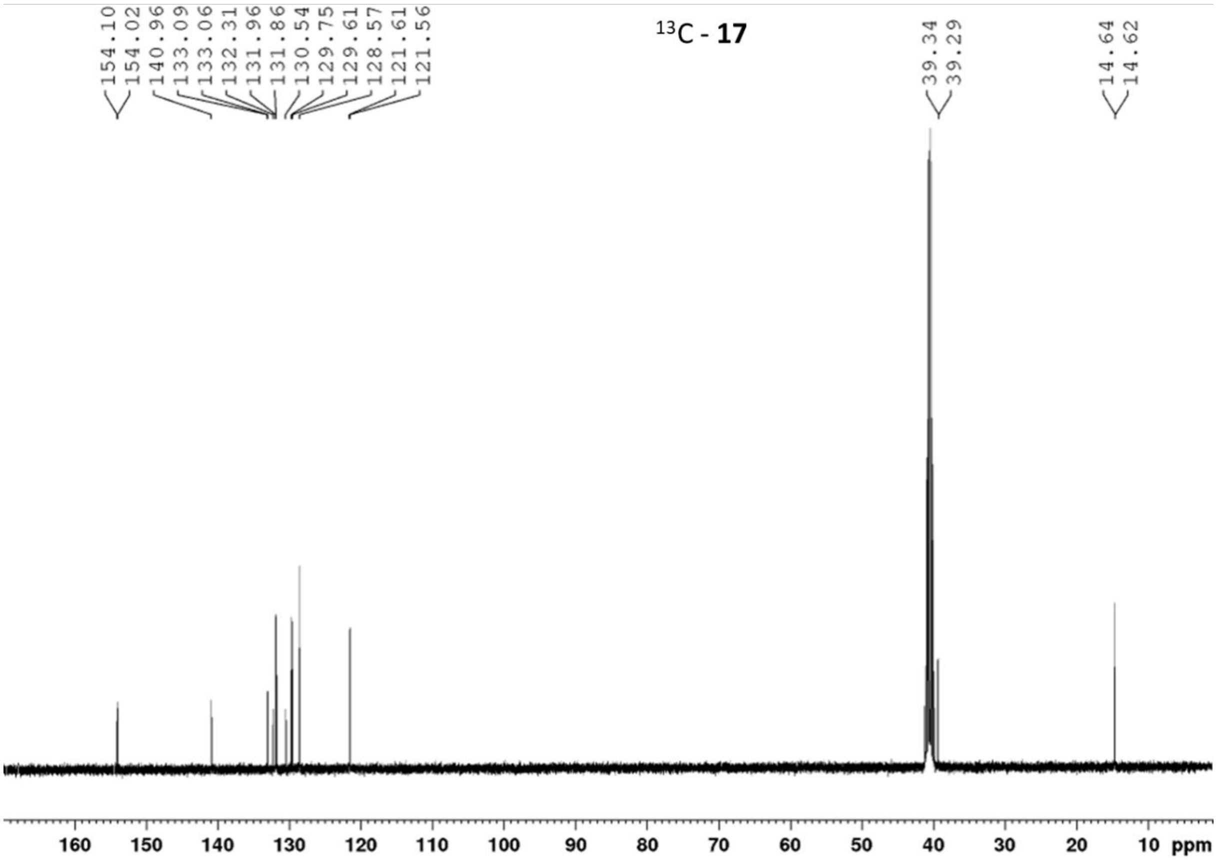

^{13}C - **17**
154.10
154.02
140.96
133.09
133.06
132.31
131.96
131.86
130.54
129.75
129.61
128.57
121.61
121.56
39.34
39.29
14.64
14.62
160 150 140 130 120 110 100 90 80 70 60 50 40 30 20 10 ppm

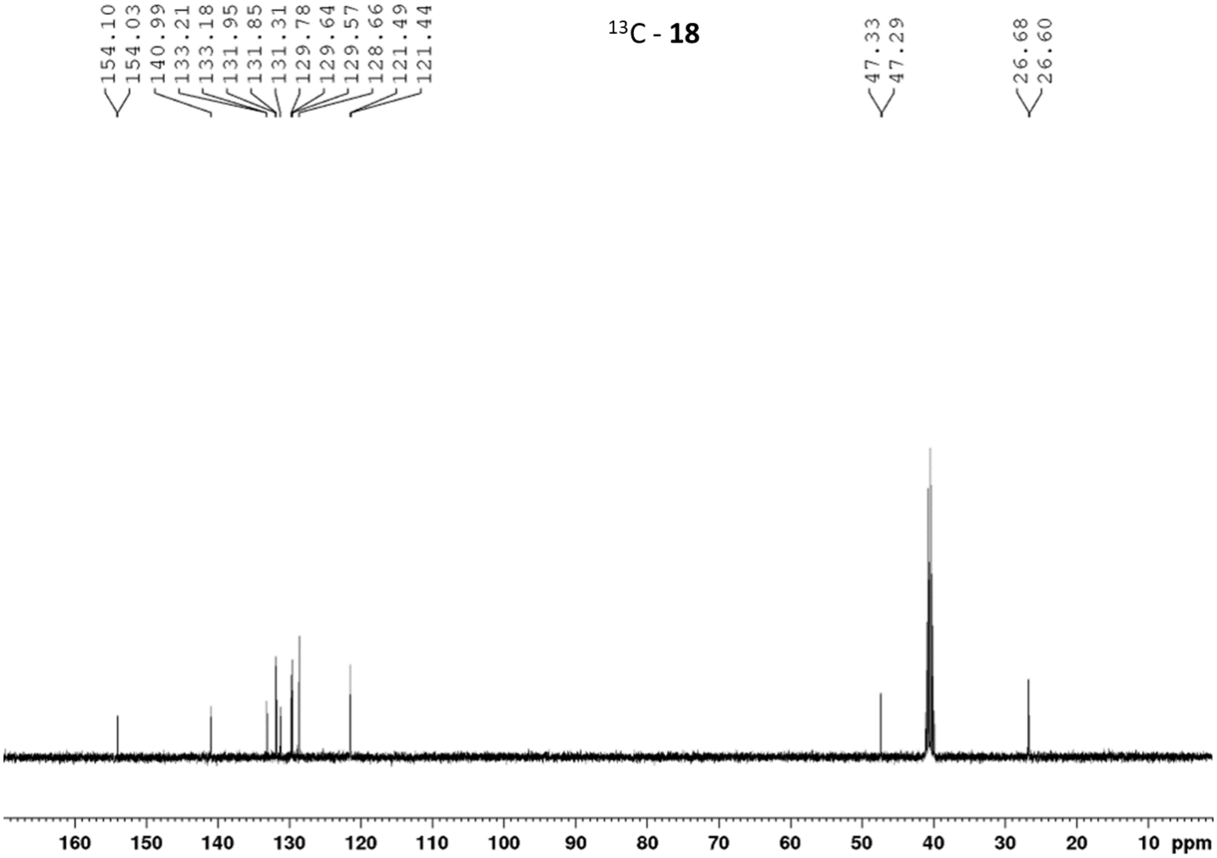

^{13}C - **18**
154.10
154.03
140.99
133.21
133.18
131.95
131.85
131.31
129.78
129.64
129.57
128.66
121.49
121.44
47.33
47.29
26.68
26.60
160 150 140 130 120 110 100 90 80 70 60 50 40 30 20 10 ppm

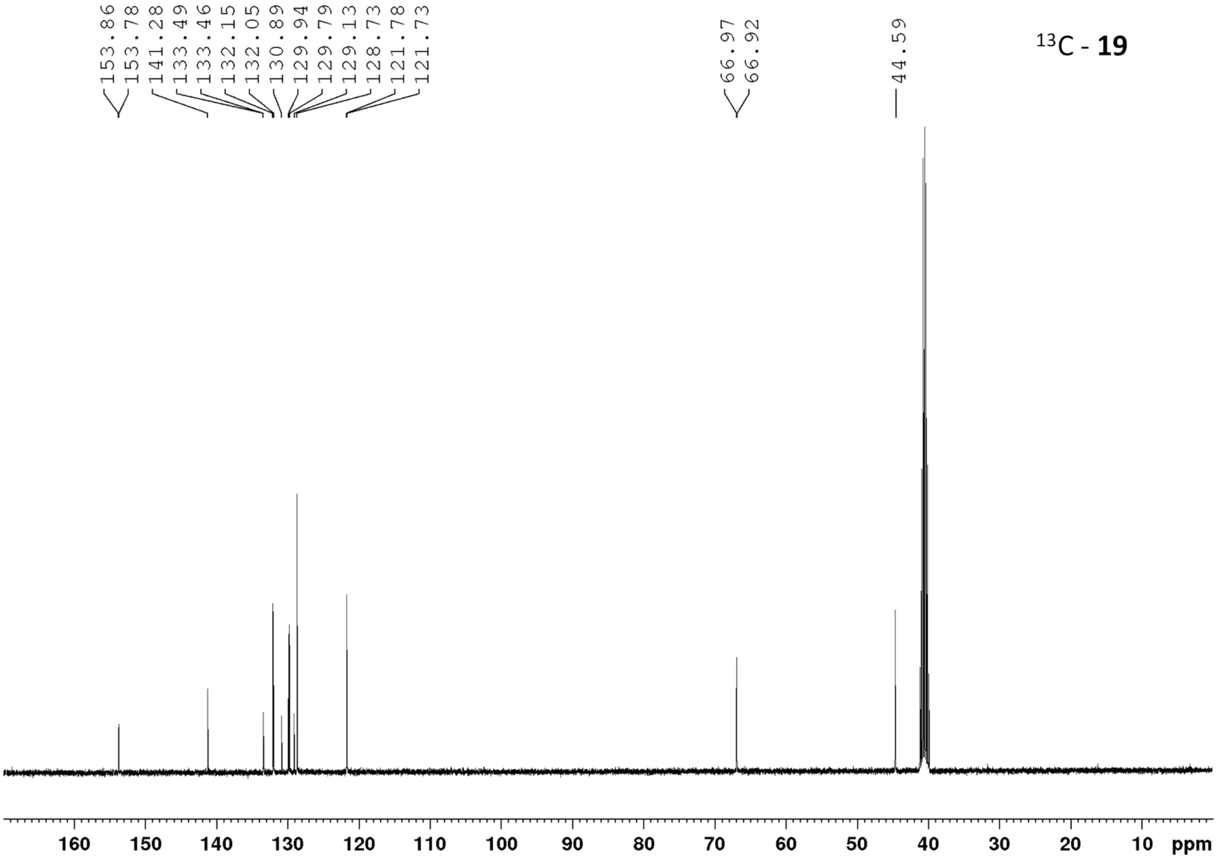
153.86
153.78
141.28
133.49
133.46
132.15
132.05
130.89
129.94
129.79
129.13
128.73
121.78
121.73
66.97
66.92
44.59
160 150 140 130 120 110 100 90 80 70 60 50 40 30 20 10 ppm

^{13}C - **19**

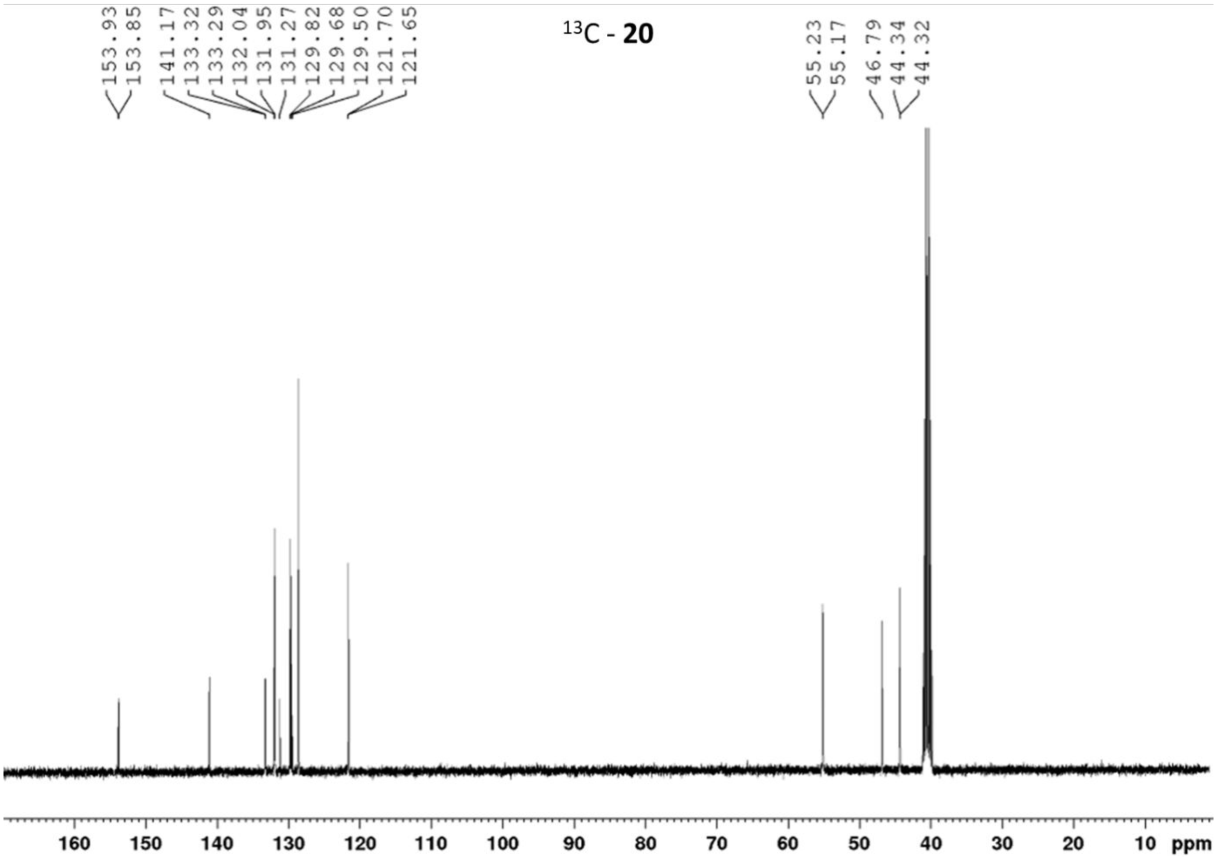
153.93
153.85
141.17
133.32
133.29
132.04
131.95
131.27
129.82
129.68
129.50
121.70
121.65
55.23
55.17
46.79
44.34
44.32
160 150 140 130 120 110 100 90 80 70 60 50 40 30 20 10 ppm

^{13}C - **20**

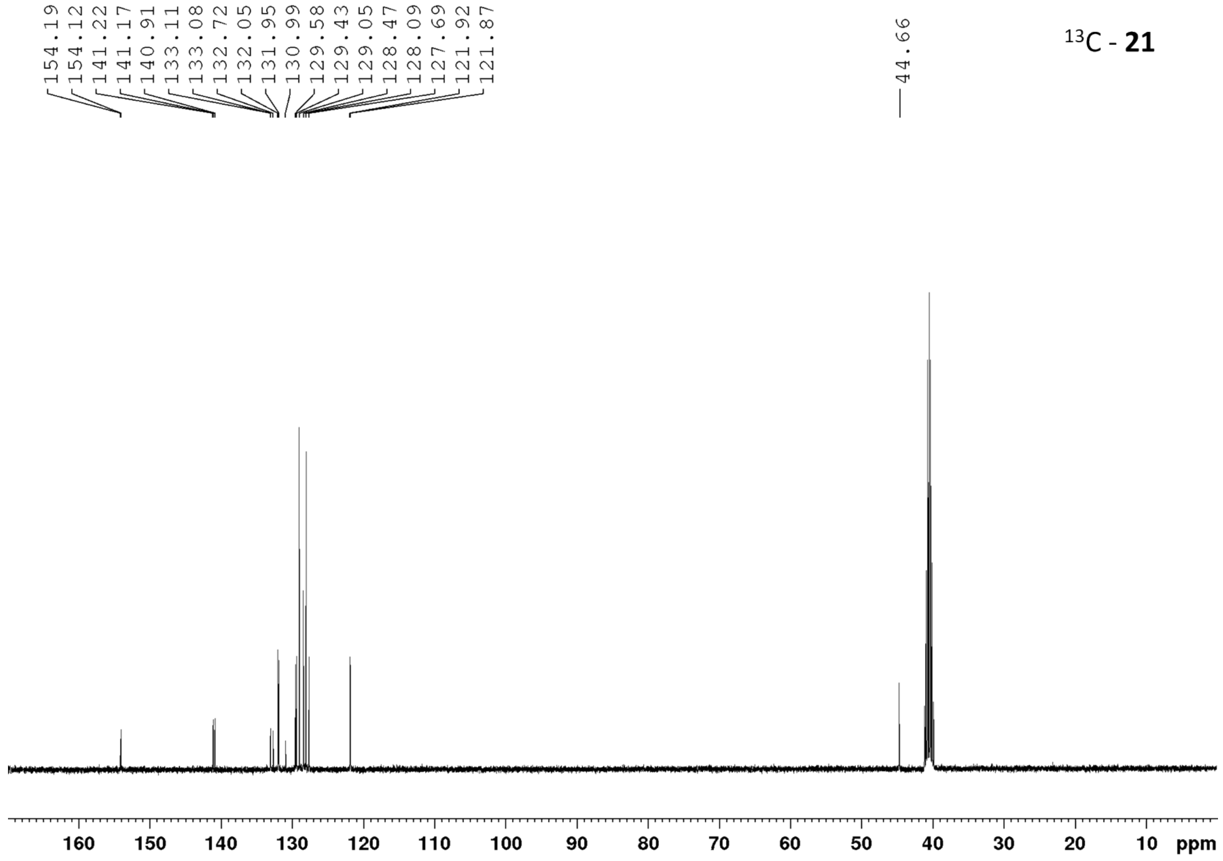
154.19
154.12
141.22
141.17
140.91
133.11
133.08
132.72
132.05
131.95
130.99
129.58
129.43
129.05
128.47
128.09
127.69
121.92
121.87
44.66
^{13}C - **21**
160 150 140 130 120 110 100 90 80 70 60 50 40 30 20 10 ppm

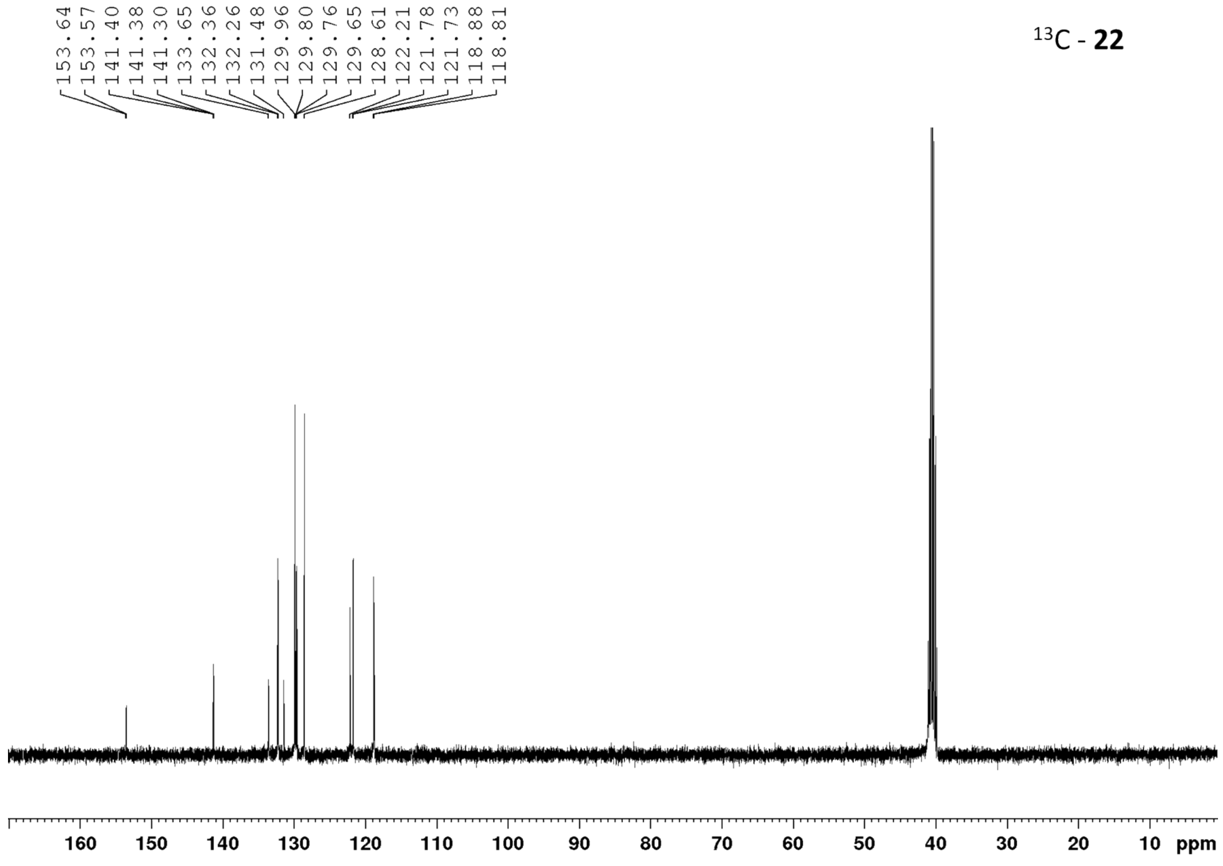
153.64
153.57
141.40
141.38
141.30
133.65
132.36
132.26
131.48
129.96
129.80
129.76
129.65
128.61
122.21
121.78
121.73
118.88
118.81
^{13}C - **22**
160 150 140 130 120 110 100 90 80 70 60 50 40 30 20 10 ppm

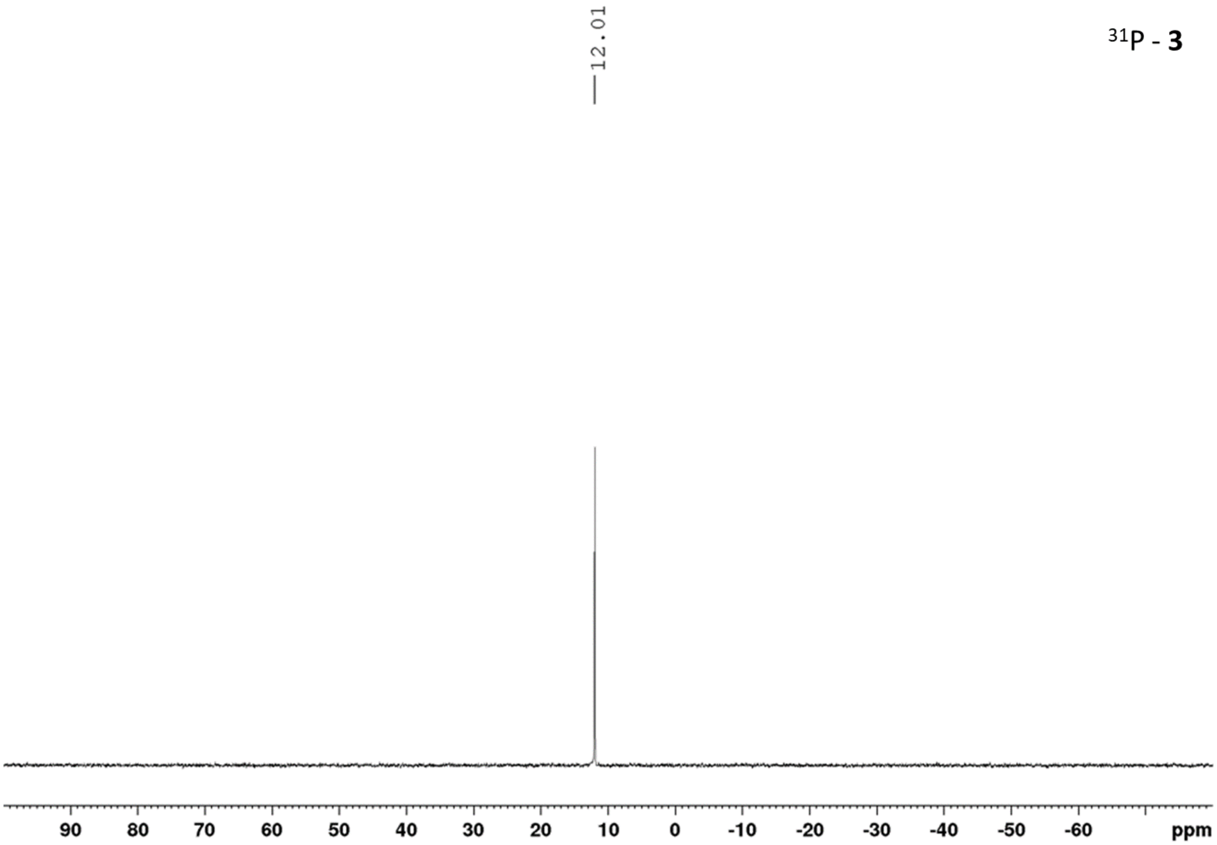
12.01
31P - 3
90 80 70 60 50 40 30 20 10 0 -10 -20 -30 -40 -50 -60 ppm

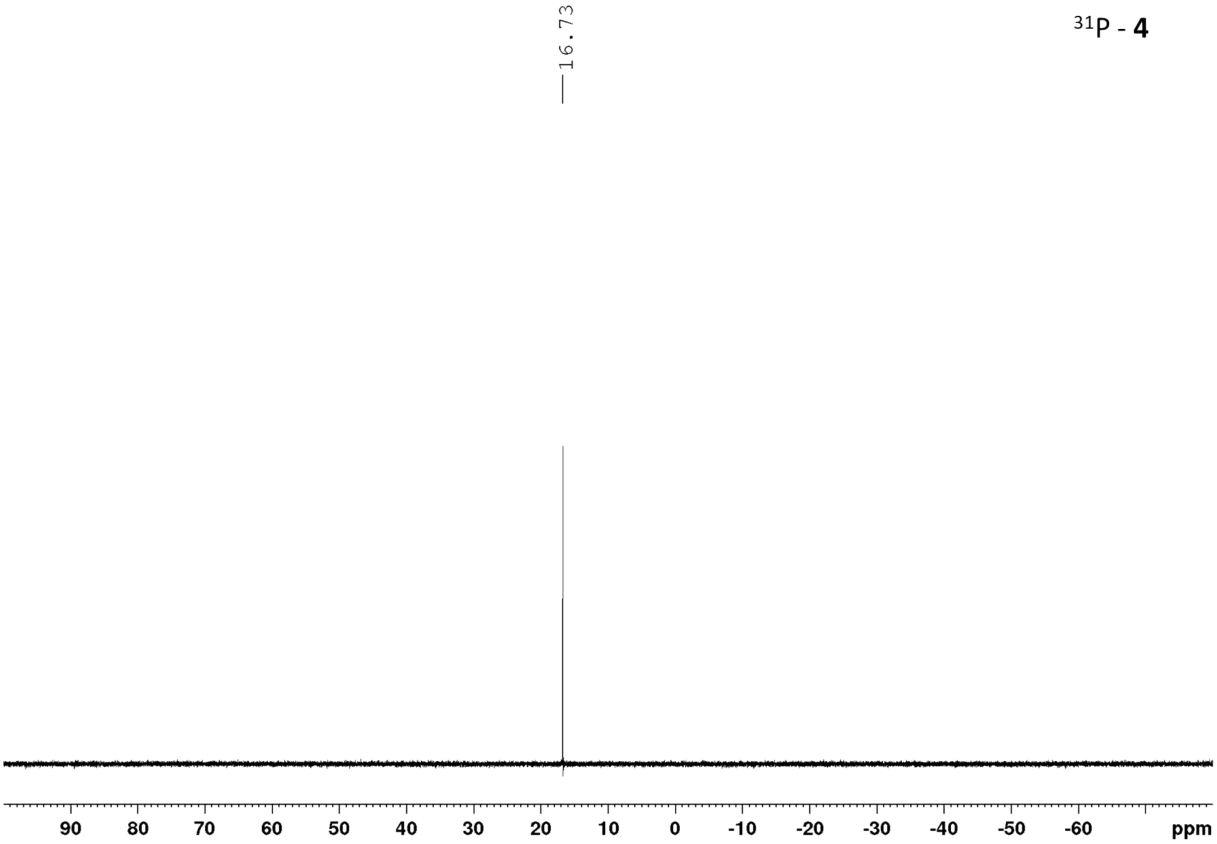
16.73
31P - 4
90 80 70 60 50 40 30 20 10 0 -10 -20 -30 -40 -50 -60 ppm

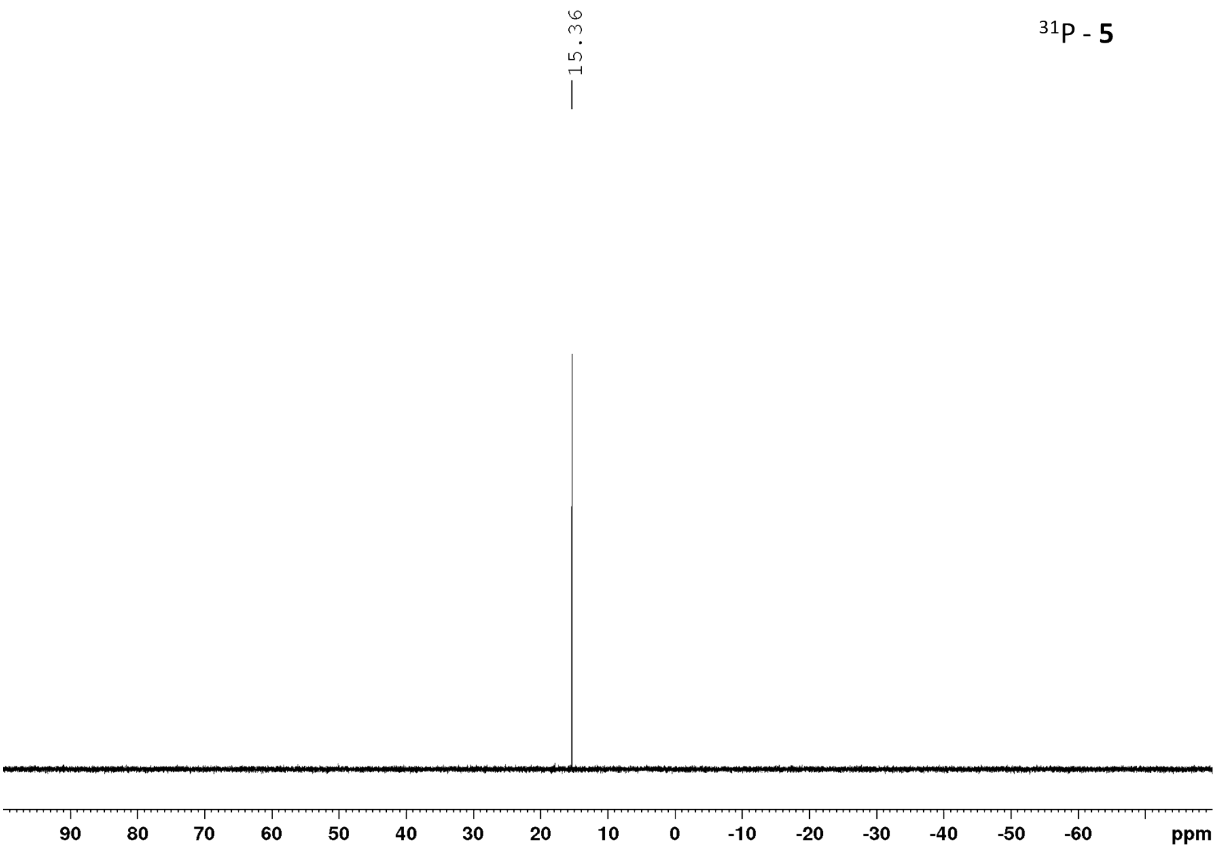
15.36
^{31}P - 5
90
80
70
60
50
40
30
20
10
0
-10
-20
-30
-40
-50
-60
ppm

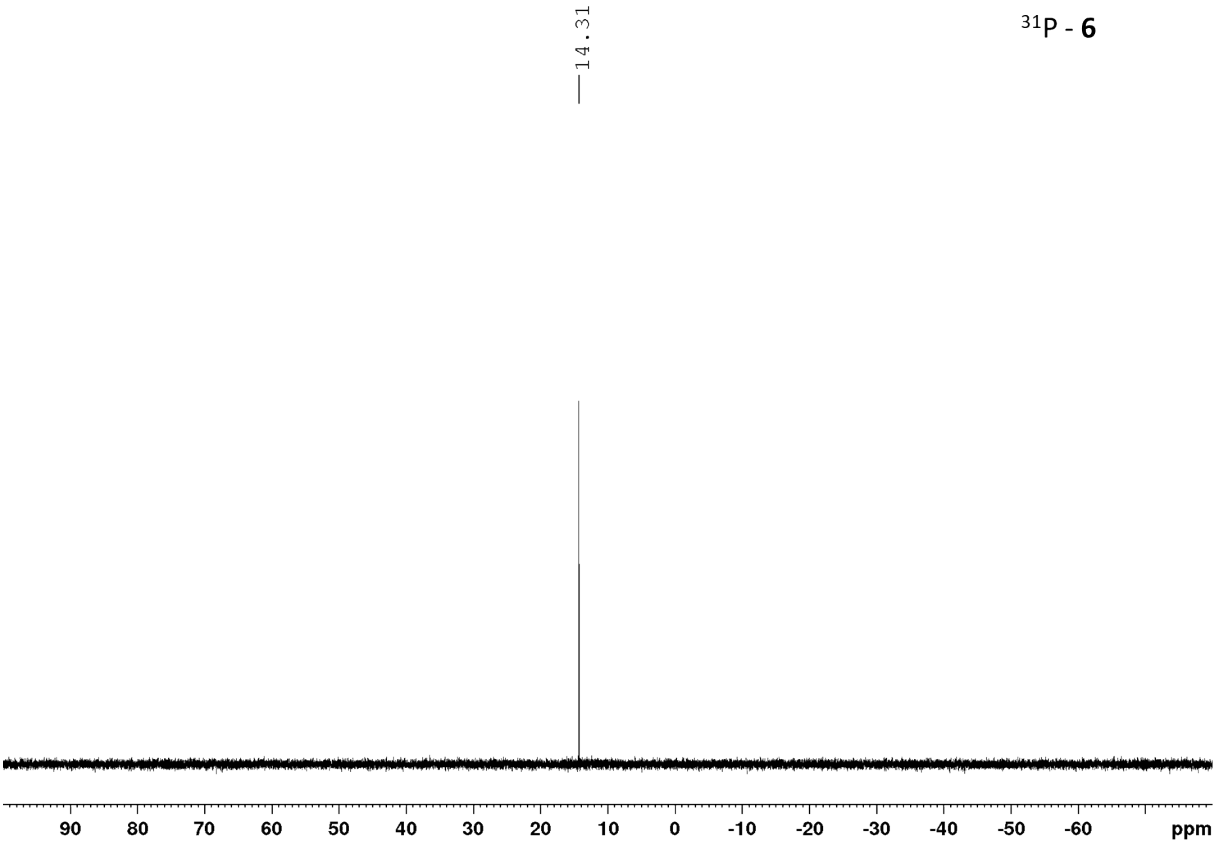
14.31
^{31}P - 6
90
80
70
60
50
40
30
20
10
0
-10
-20
-30
-40
-50
-60
ppm

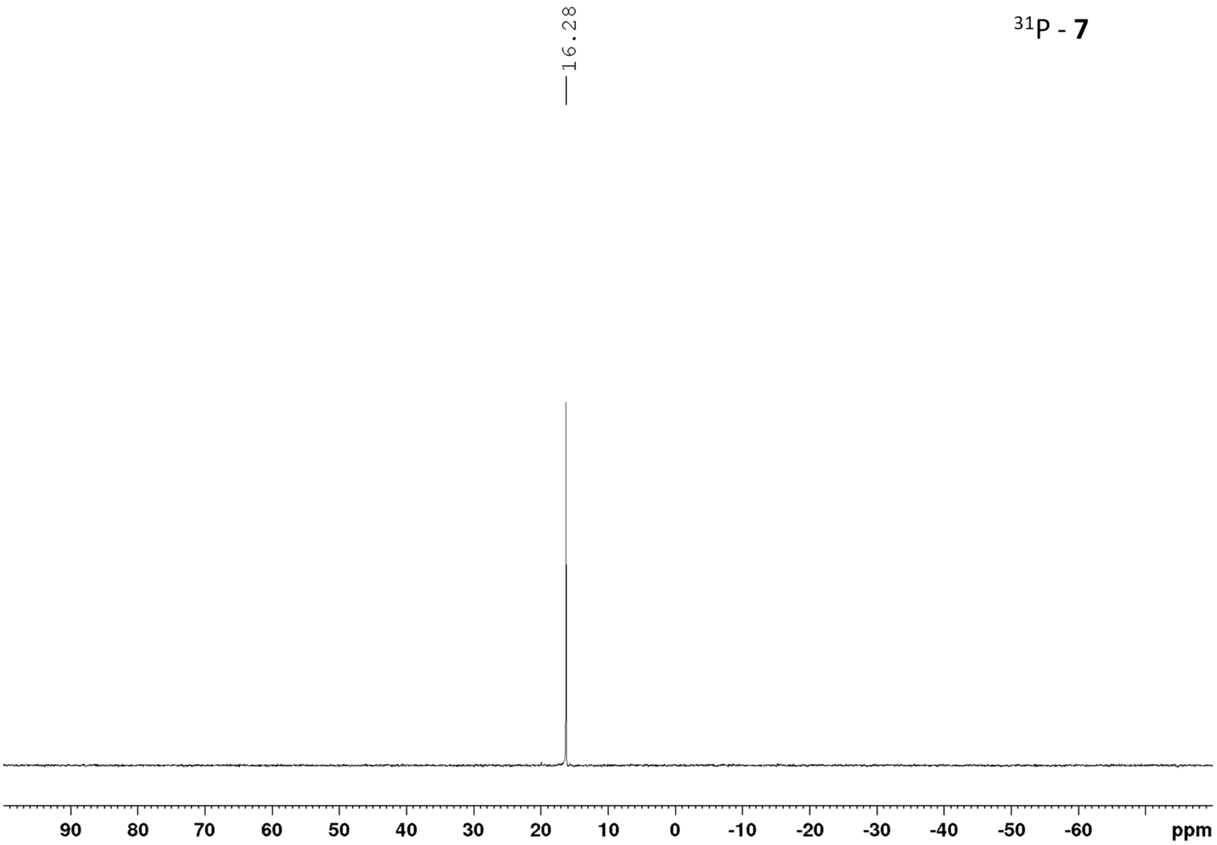
16.28
90 80 70 60 50 40 30 20 10 0 -10 -20 -30 -40 -50 -60 ppm

^{31}P - **7**

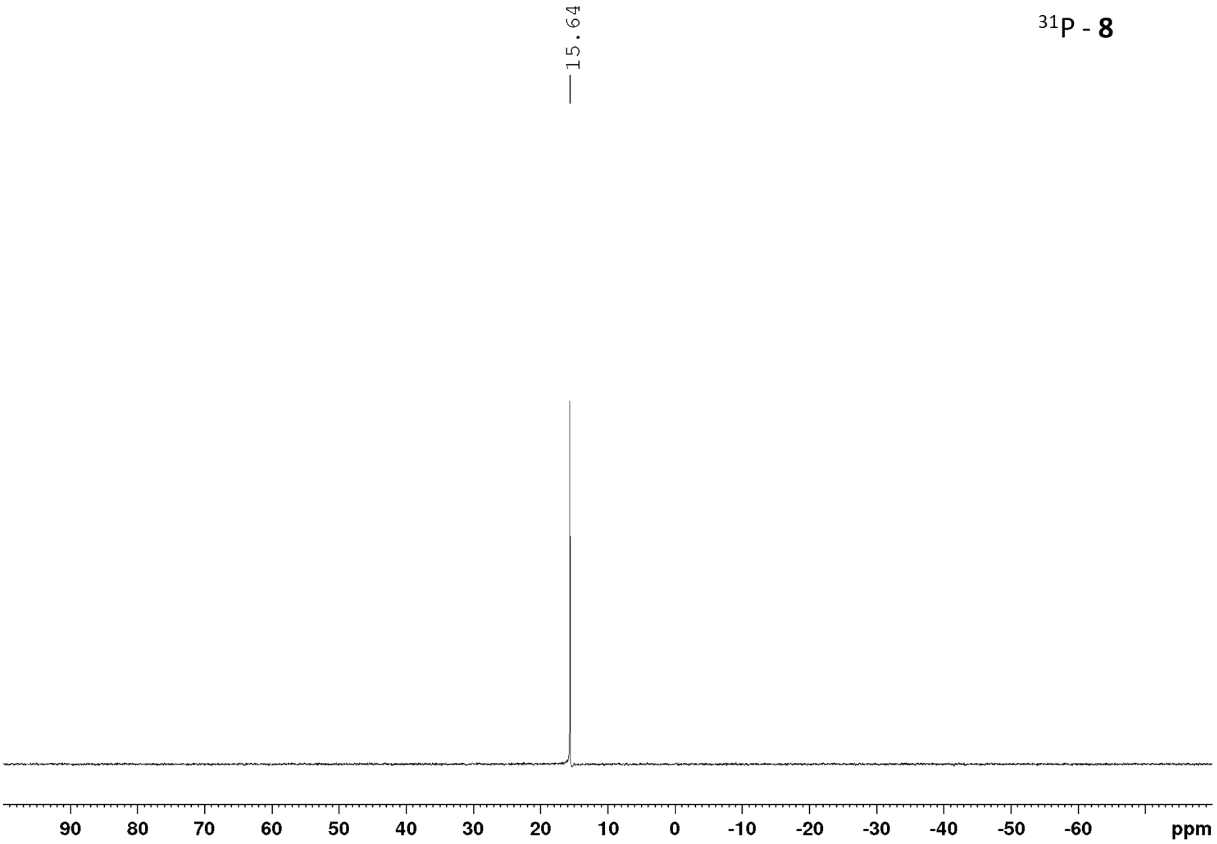
15.64
90 80 70 60 50 40 30 20 10 0 -10 -20 -30 -40 -50 -60 ppm

^{31}P - **8**

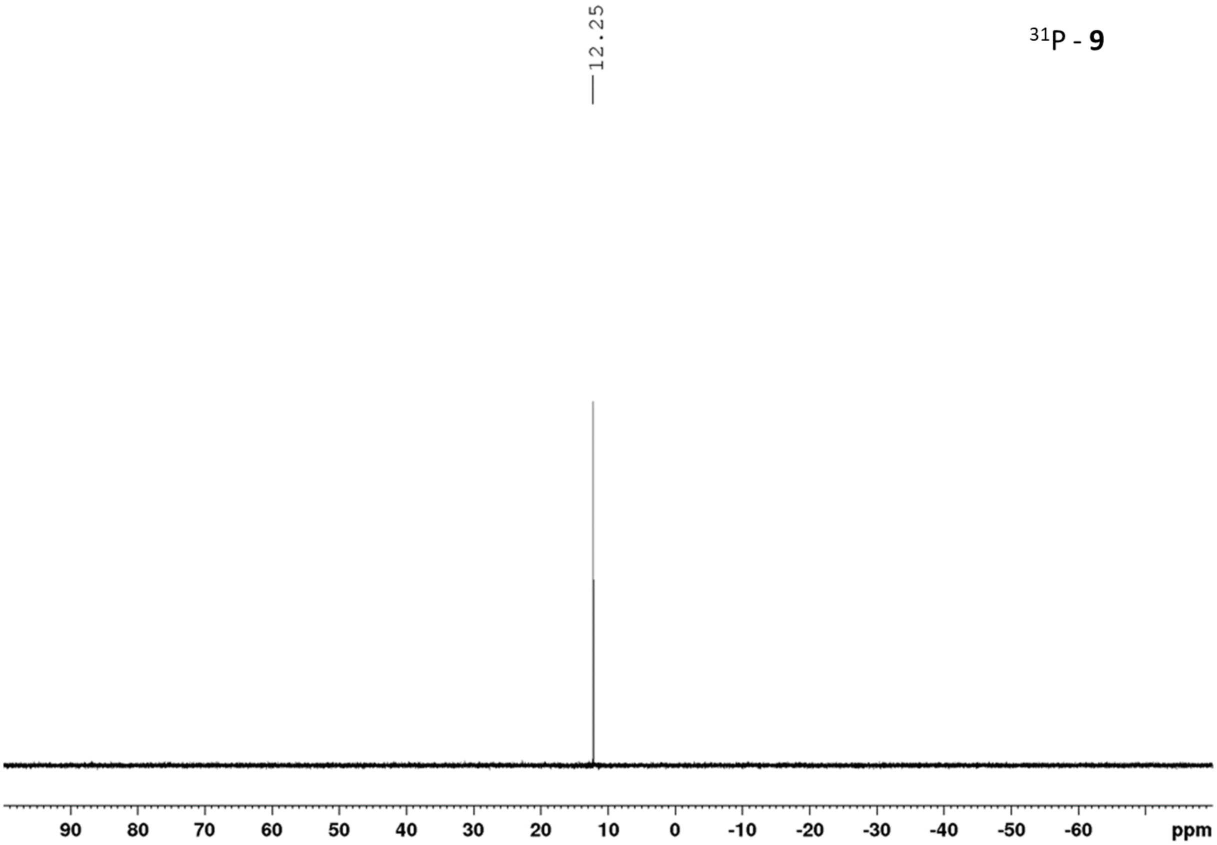
12.25
^{31}P - **9**
90 80 70 60 50 40 30 20 10 0 -10 -20 -30 -40 -50 -60 ppm

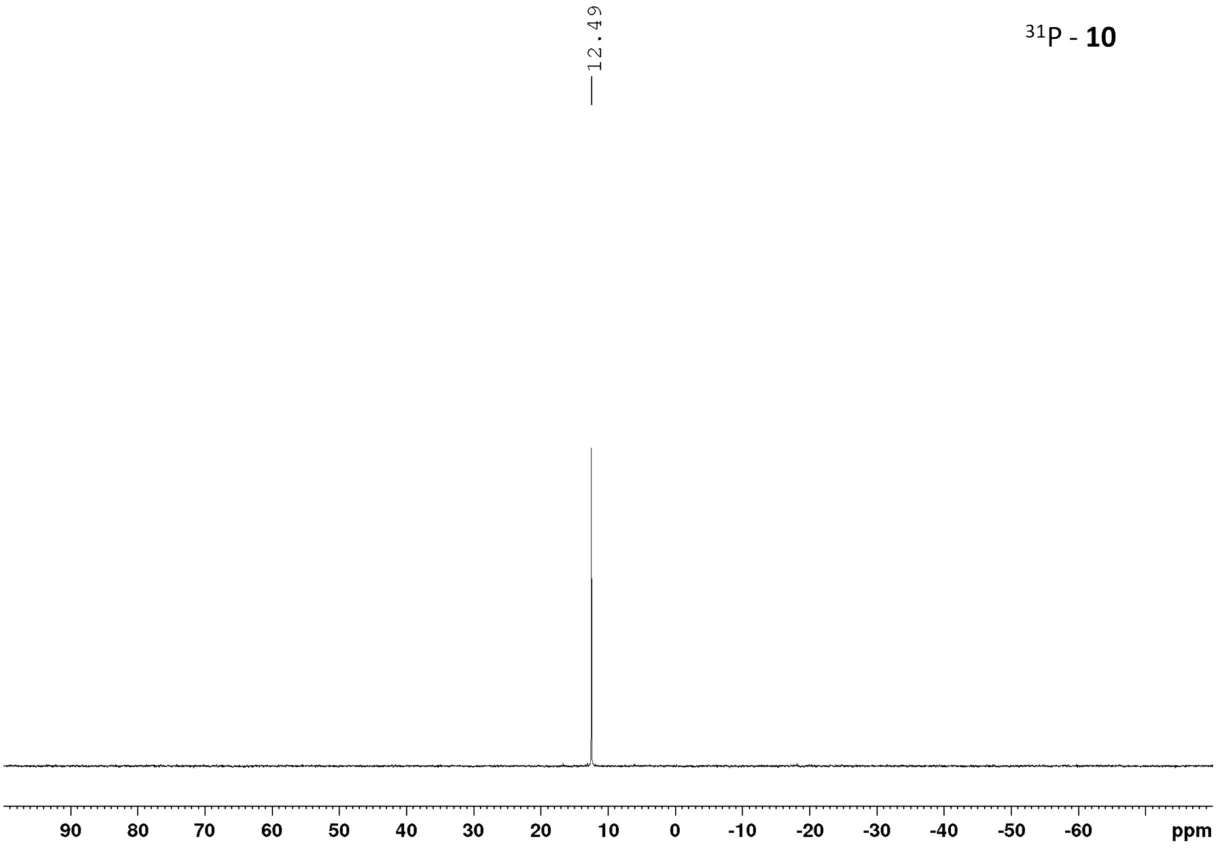
12.49
^{31}P - **10**
90 80 70 60 50 40 30 20 10 0 -10 -20 -30 -40 -50 -60 ppm

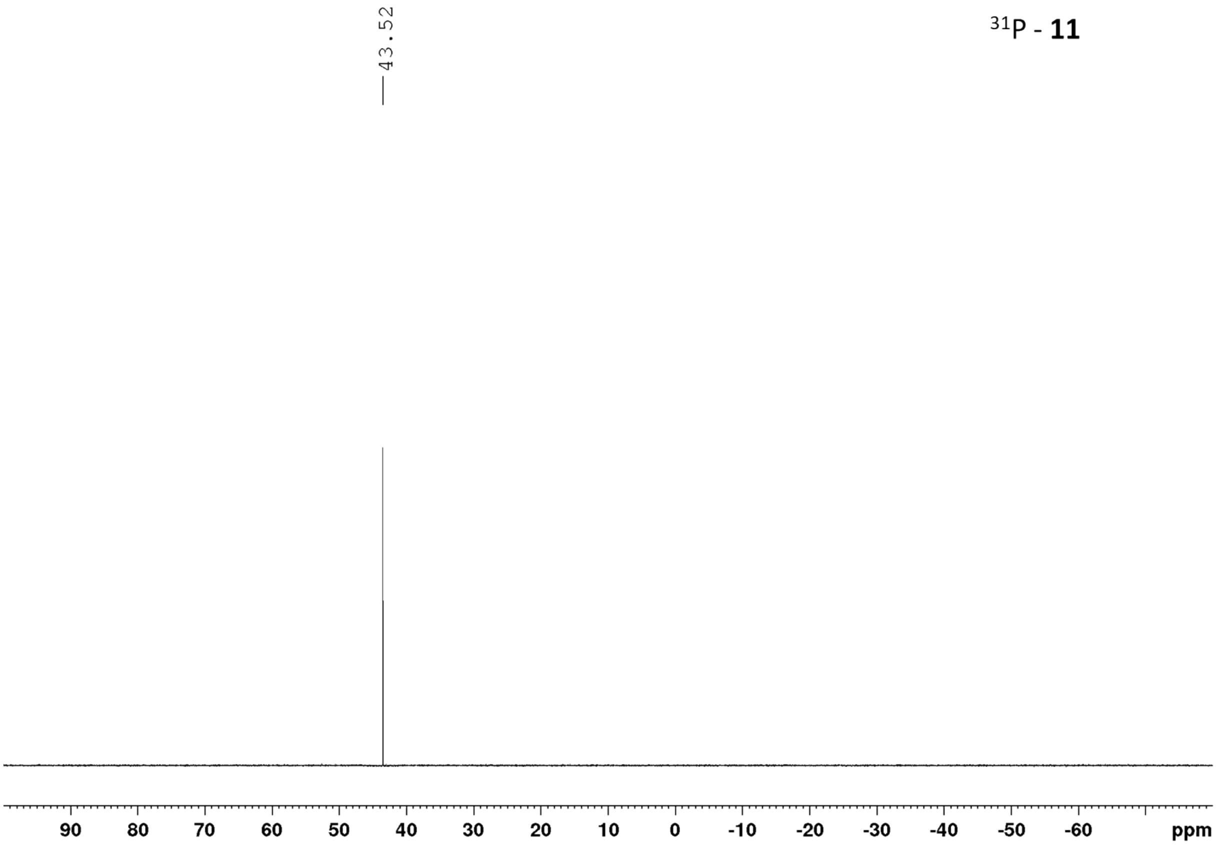

^{31}P - **11**

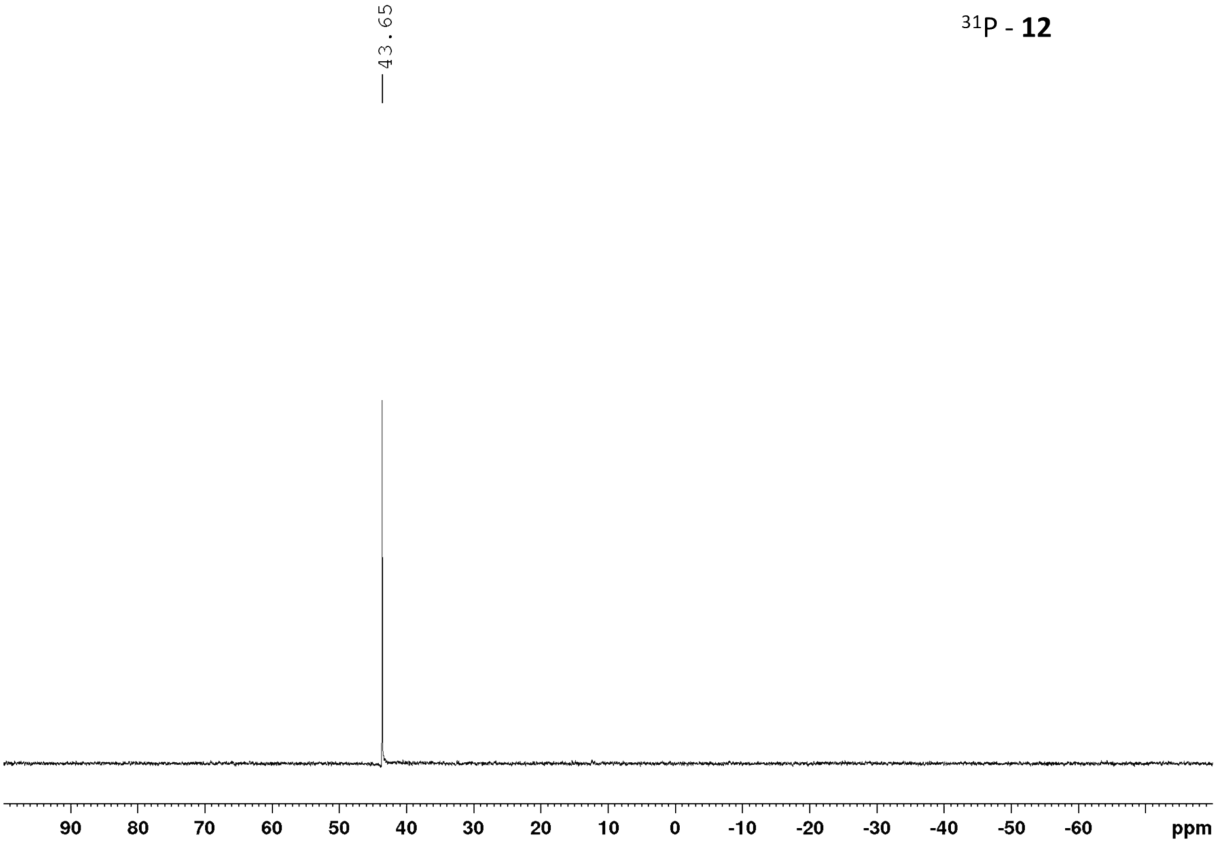

^{31}P - **12**

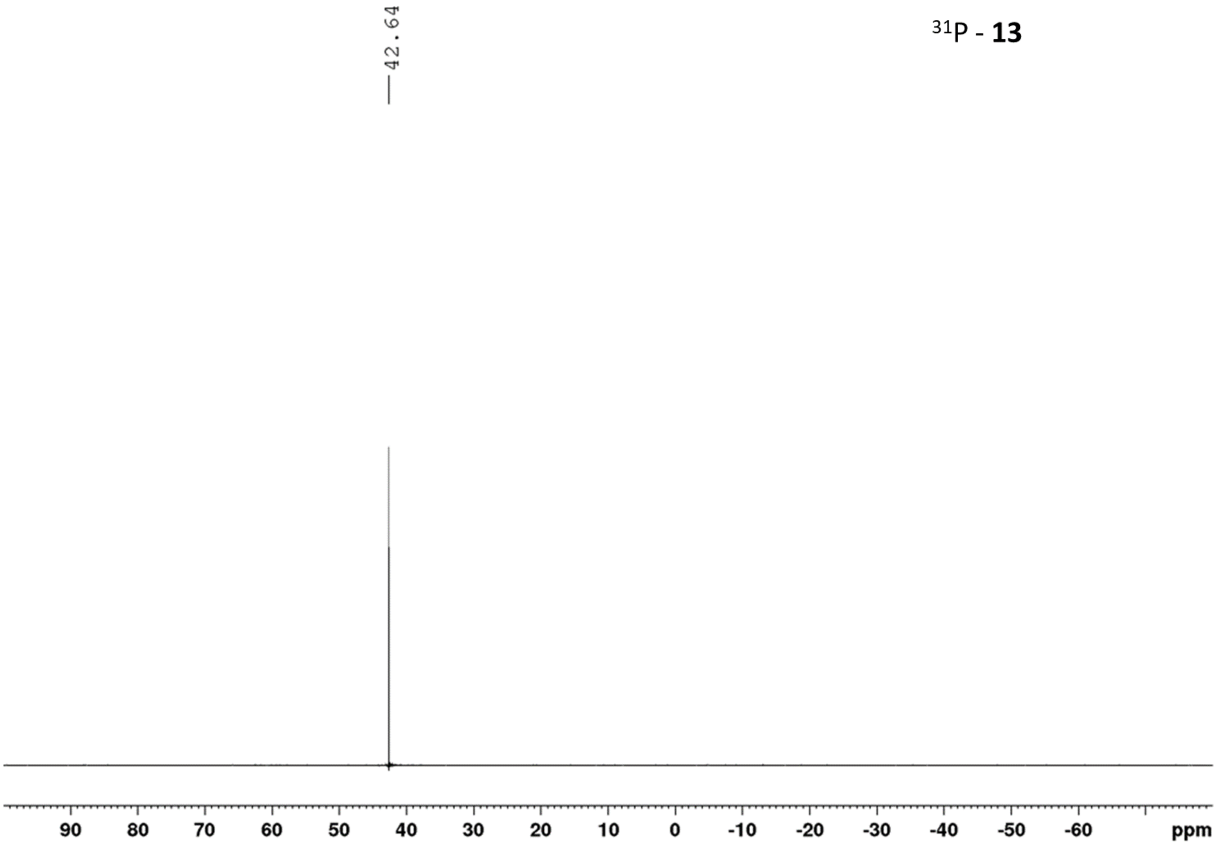
42.64
³¹P - 13
90
80
70
60
50
40
30
20
10
0
-10
-20
-30
-40
-50
-60
ppm

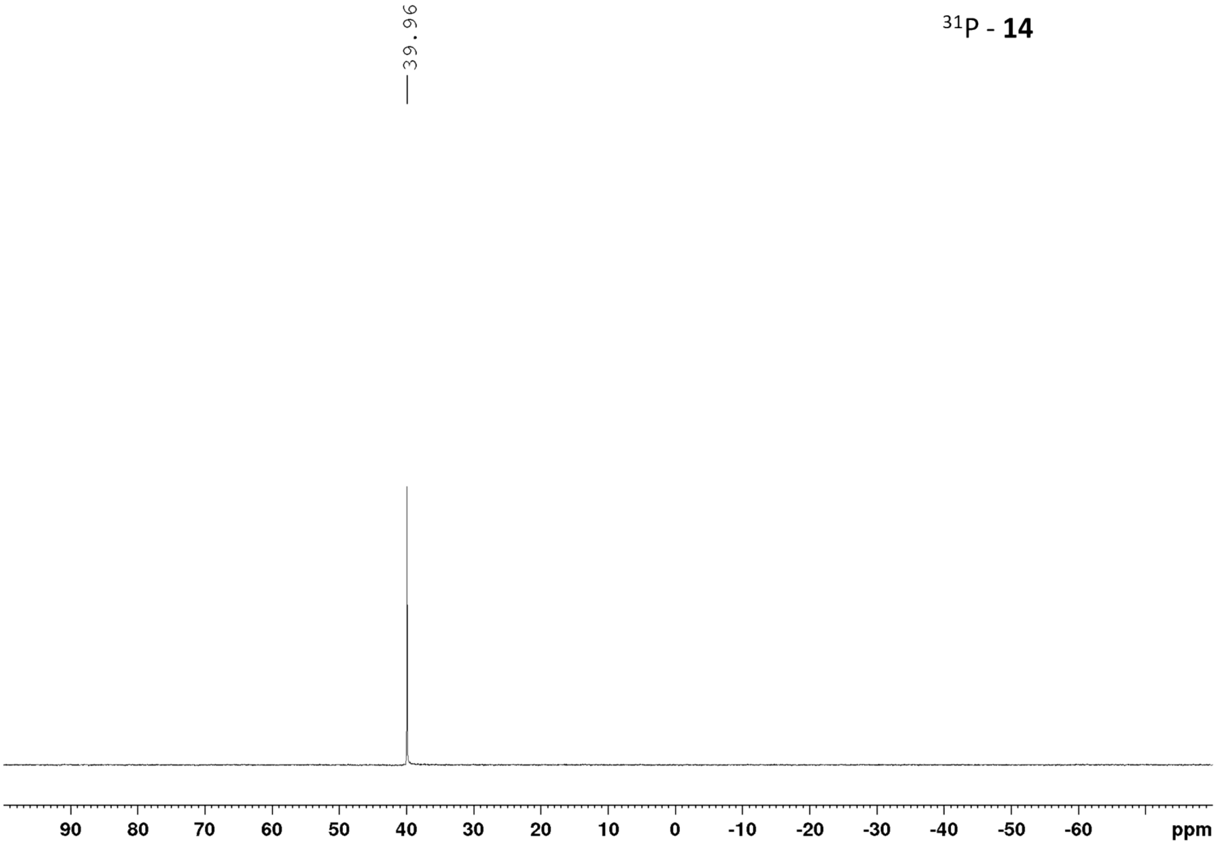
39.96
³¹P - 14
90
80
70
60
50
40
30
20
10
0
-10
-20
-30
-40
-50
-60
ppm

^{31}P - **15**

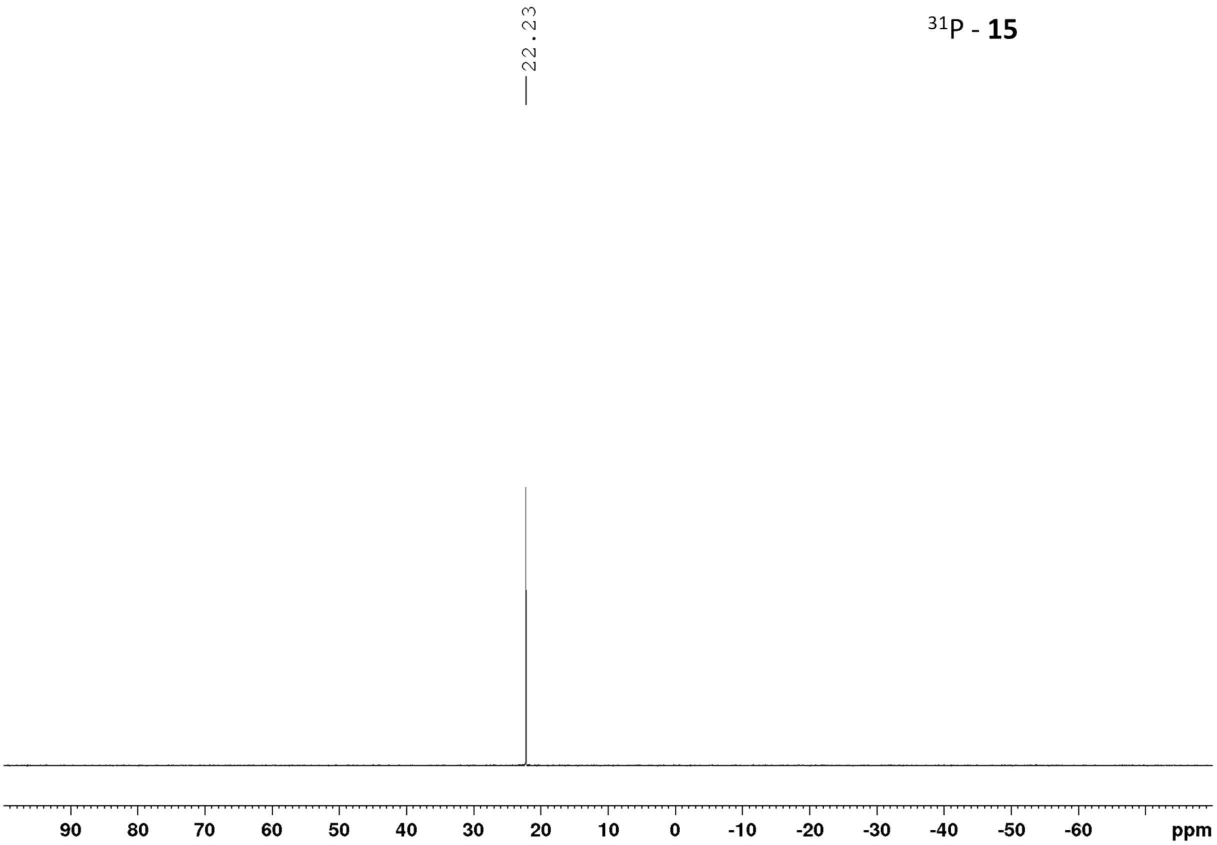
22.23
90
80
70
60
50
40
30
20
10
0
-10
-20
-30
-40
-50
-60
ppm

^{31}P - **16**

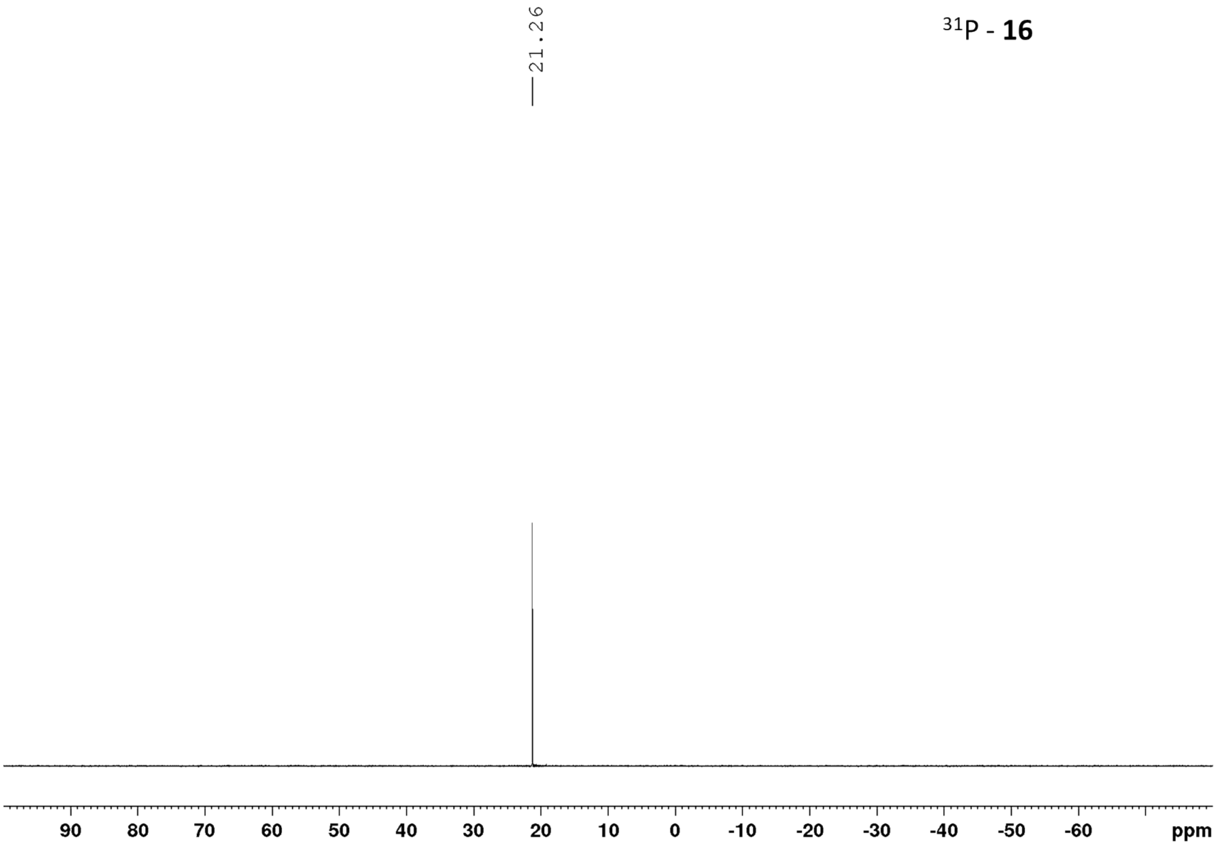
21.26
90
80
70
60
50
40
30
20
10
0
-10
-20
-30
-40
-50
-60
ppm

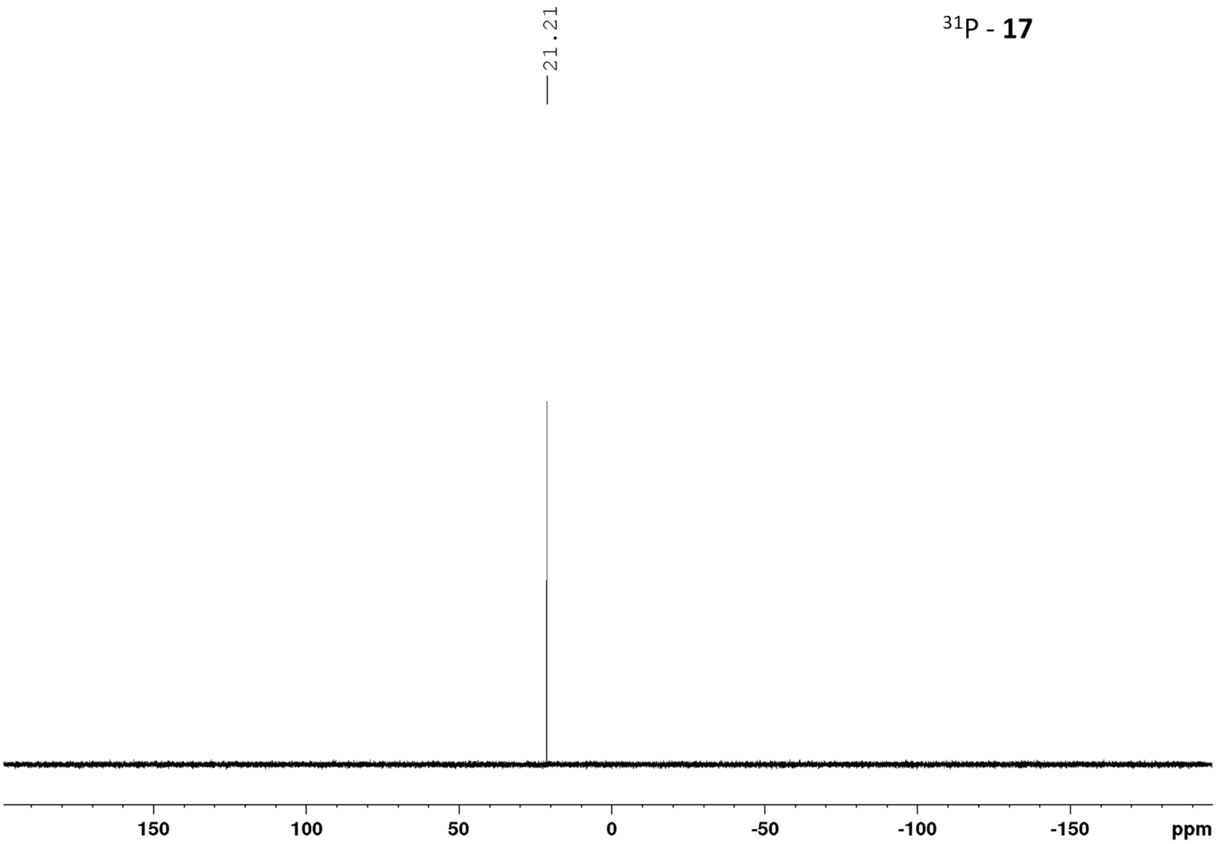

21.21
^{31}P - **17**
150
100
50
0
-50
-100
-150
ppm

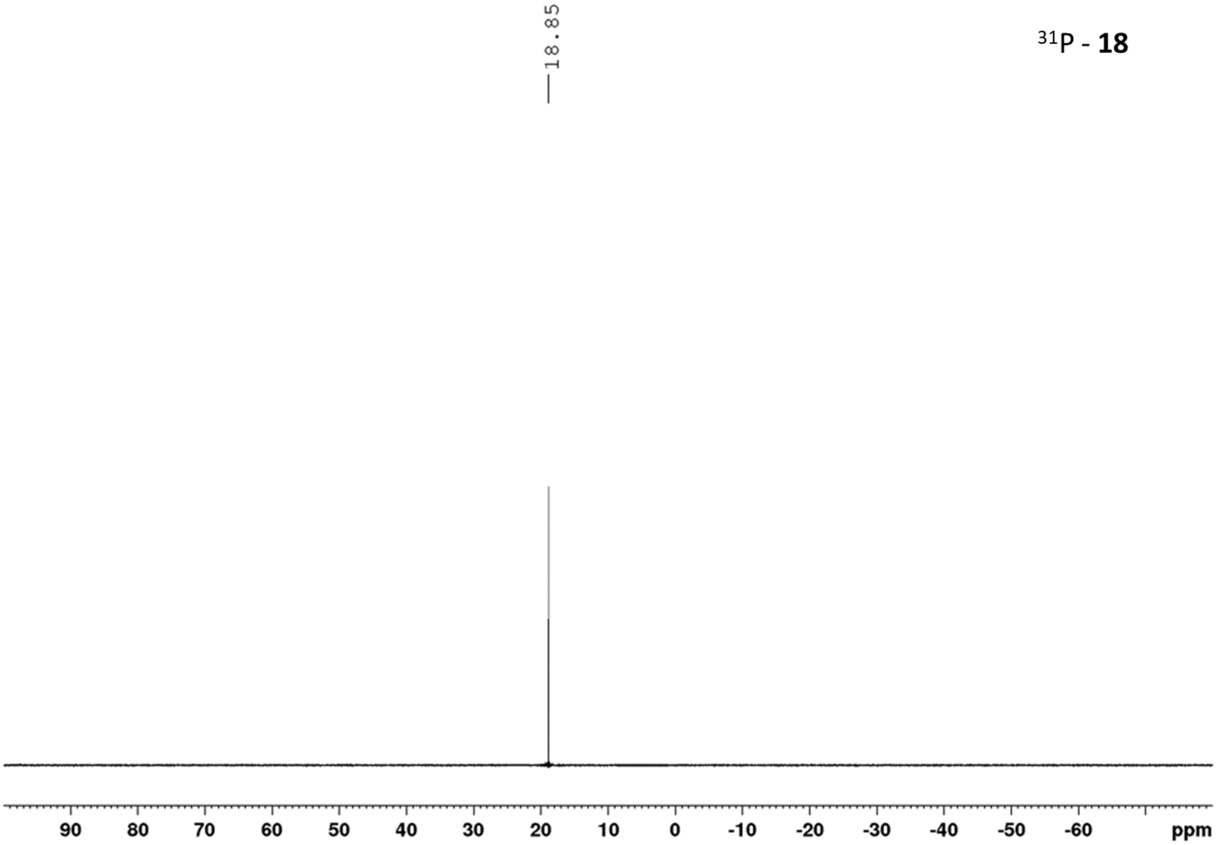

18.85
^{31}P - **18**
90
80
70
60
50
40
30
20
10
0
-10
-20
-30
-40
-50
-60
ppm

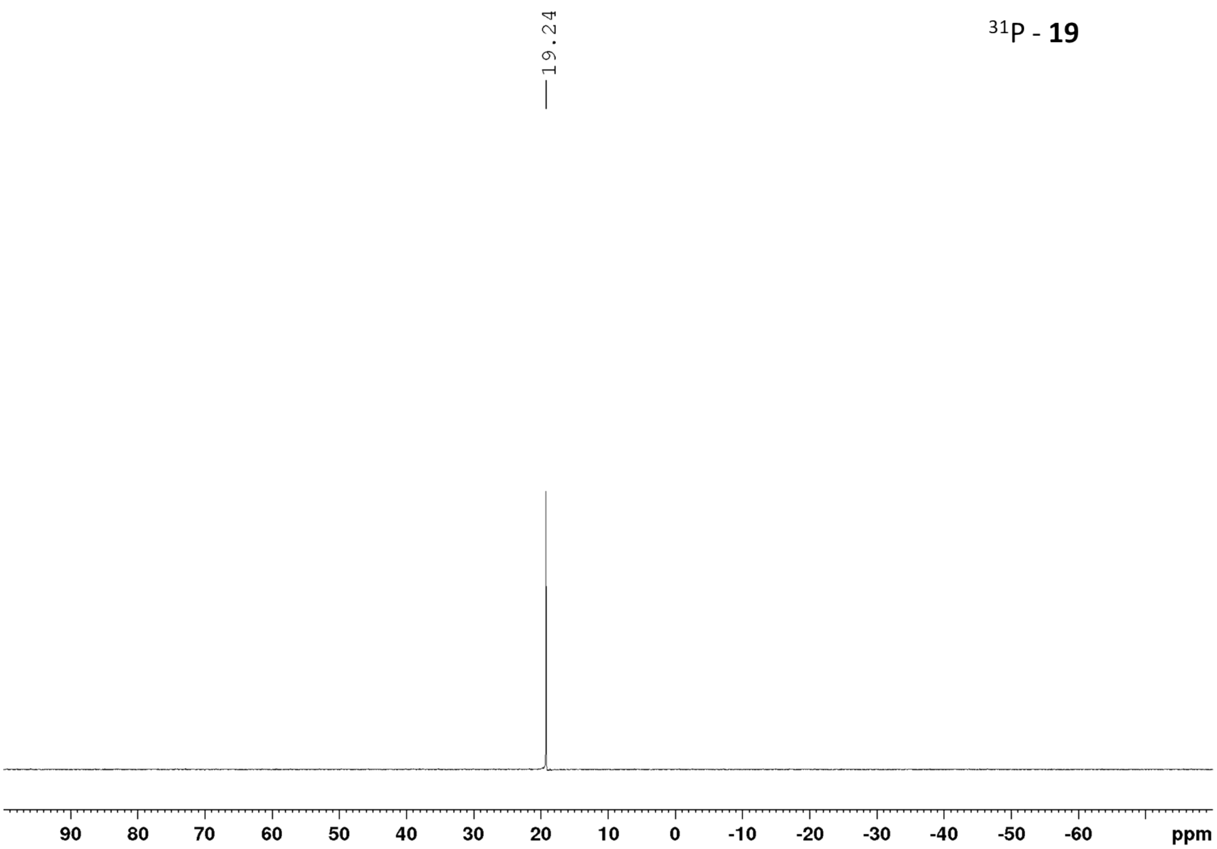
19.24
^{31}P - **19**
90
80
70
60
50
40
30
20
10
0
-10
-20
-30
-40
-50
-60
ppm

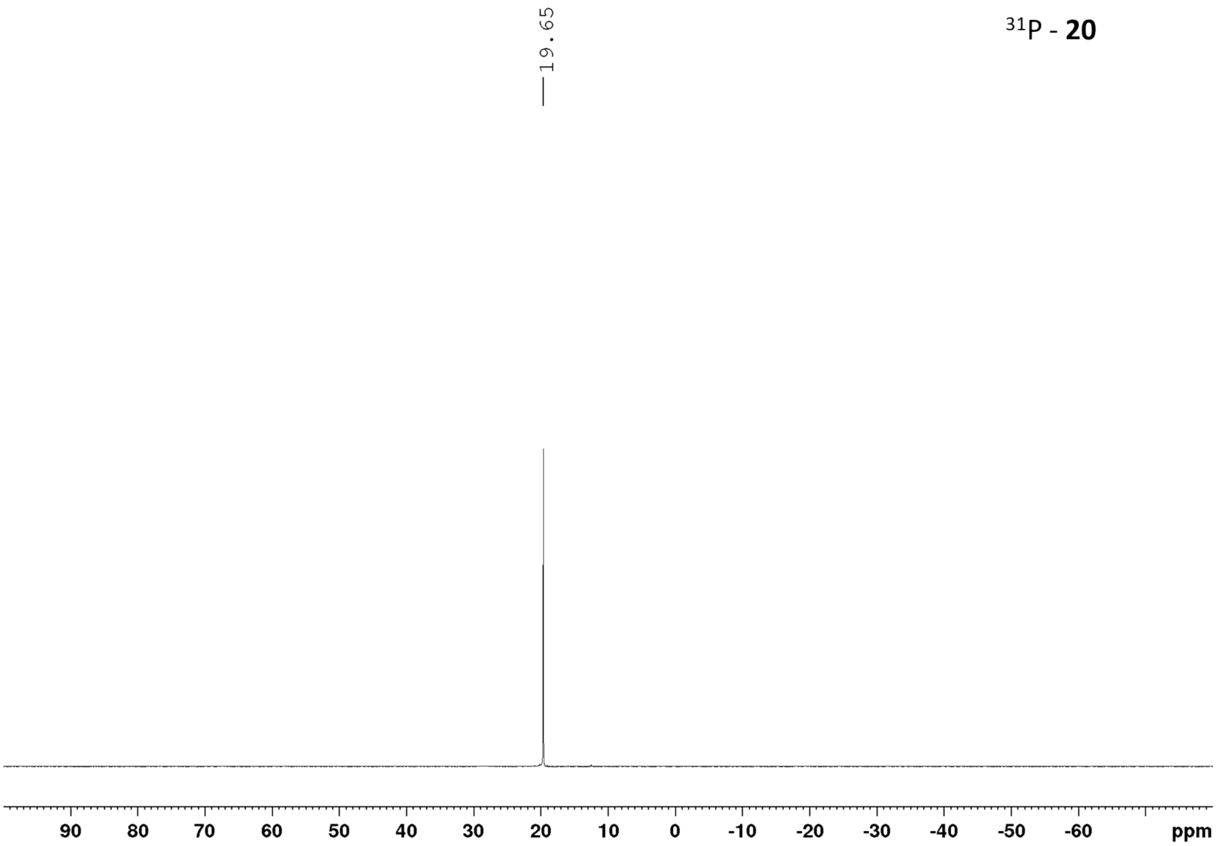
19.65
^{31}P - **20**
90
80
70
60
50
40
30
20
10
0
-10
-20
-30
-40
-50
-60
ppm

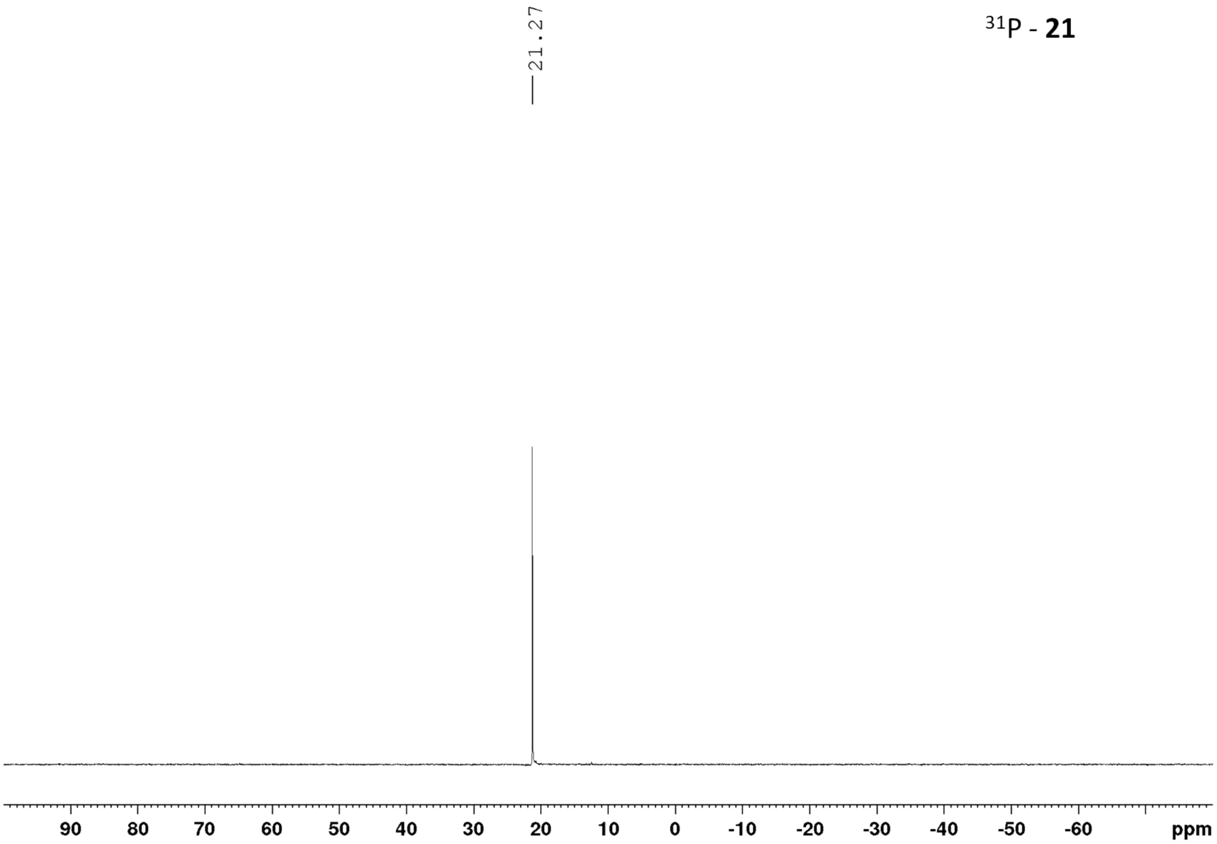

21.27
³¹P - 21
90
80
70
60
50
40
30
20
10
0
-10
-20
-30
-40
-50
-60
ppm

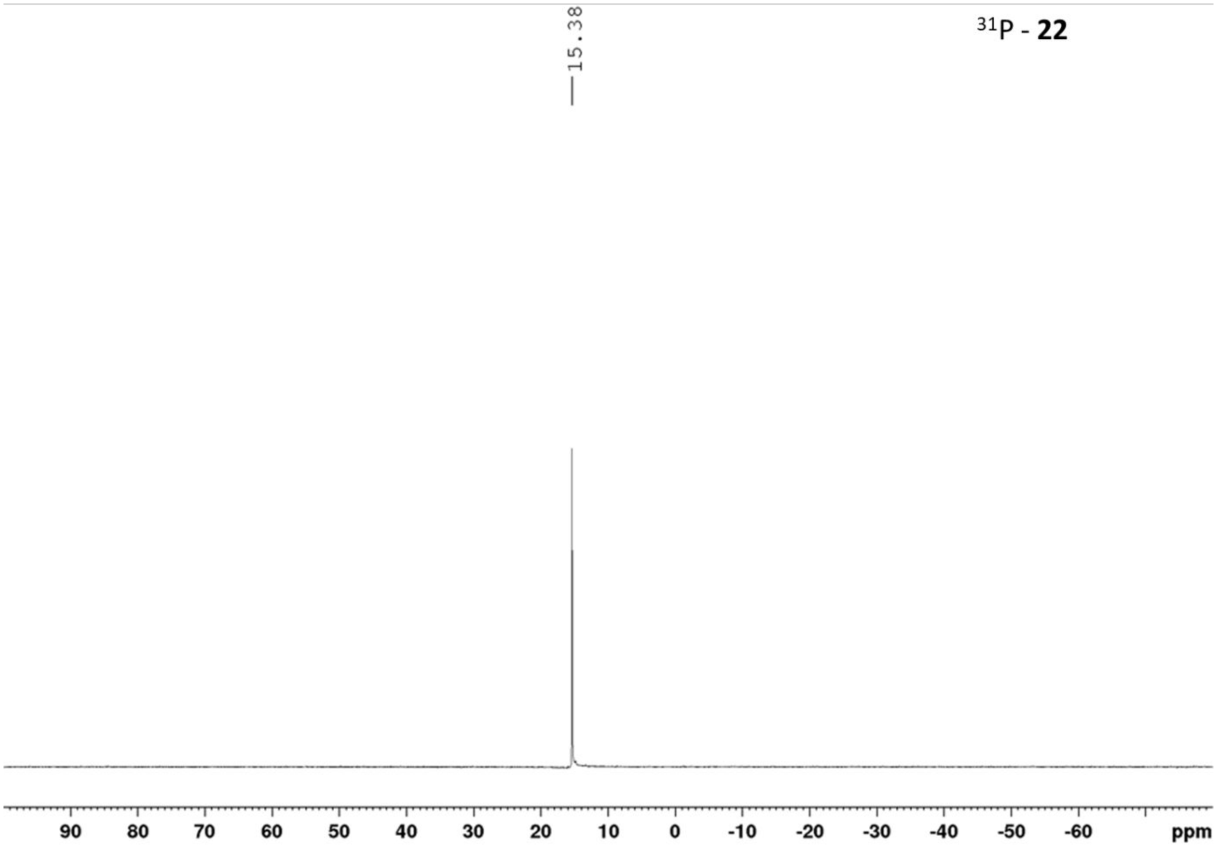

15.38
³¹P - 22
90
80
70
60
50
40
30
20
10
0
-10
-20
-30
-40
-50
-60
ppm

Table S1. Data collection and refinement statistics for CA II/**3** and CA VII/**3** complexes.

	6SDS	6SDT
Crystal parameters		
Space group	$P2_1$	$P2_12_12$
a (Å)	42.2	66.3
b (Å)	41.2	89.4
c (Å)	71.9	44.2
α (°)	90	90
β (°)	104.1	90
γ (°)	90	90
Data collection statistics		
Resolution (Å)	35.5-1.26 (1.28-1.26)	34.0-1.94 (1.97-1.94)
Temperature (K)	100	100
Total reflections	237816	117966
Unique reflections	64217	20119
Completeness (%)	98.5 (77.6)	99.9 (99.8)
$\langle I\rangle/\langle\sigma(I)\rangle$	20.9 (2.3)	16.5 (2.5)
Redundancy (%)	3.7 (1.8)	5.9 (3.6)
R_{merge}^{a}	0.054 (0.399)	0.084 (0.539)
R_{meas}^{a}	0.061 (0.498)	0.092 (0.630)
R_{pim}^{a}	0.028 (0.292)	0.037 (0.321)
Refinement statistics		
Resolution (Å)	35.50-1.26	33.99-1.94
R_{work}^{b} (%)	14.6	18.8
R_{free}^{b} (%)	17.4	22.6
r.m.s.d. from ideal geometry:		
Bond lengths (Å)	0.011	0.010
Bond angles (°)	1.7	1.8
Number of protein atoms	2072	2054
Number of inhibitor atoms	20	20
Number of water molecules	190	94
Average B factor (Å^2)		
All atoms	12.0	21.0
Protein atoms	11.4	20.9
Inhibitor atoms	13.6	20.8
Water molecules	18.5	22.3

$^{a}R_{merge} = \Sigma_{hkl}\Sigma_i|I_i(hkl)-\langle I(hkl)\rangle|/\ \Sigma_{hkl}\Sigma_i I_i(hkl)$; $R_{meas} = \Sigma_{hkl}\{n(hkl)/[n(hkl)-1]\}^{1/2}\Sigma_i|I_i(hkl)-\langle I(hkl)\rangle|/\ \Sigma_{hkl}\Sigma_i I_i(hkl)$; $R_{pim} = \Sigma_{hkl}\{1/[n(hkl)-1]\}^{1/2}\Sigma_i|I_i(hkl)-\langle I(hkl)\rangle|/\ \Sigma_{hkl}\Sigma_i I_i(hkl)$, where $I_i(hkl)$ is the intensity of an observation and $\langle I(hkl)\rangle$ is the mean value for its unique reflection; summations are over all "n" reflections.

$^{b}R_{factor} = \Sigma_h||F_o(h)|-|F_c(h)||/\Sigma_h|F_o(h)|$, where F_o and F_c are the observed and calculated structure-factor amplitudes, respectively. R_{free} was calculated with 2.2% of the data excluded from the refinement.

Table S2. Number of hydrophobic interactions (<4 Å) between CA II/VII residues and compound **3**

Ligand portion	Residue	CA II	CA VII
**Benzenesulfonammide**	His94	1	2
	Val121	1	3
	Leu198	8	10
**Phenyl tail**	Phe131	2	2
	Pro202	2	-
**Total**		14	17

Drug stability profiles in solution

The solution stability profiles of tested compounds in PBS, human plasma and $HCl_{(aq)}$ 10 mM were obtained by monitoring the variation of analyte concentration at different incubation times (Figures S1-5).

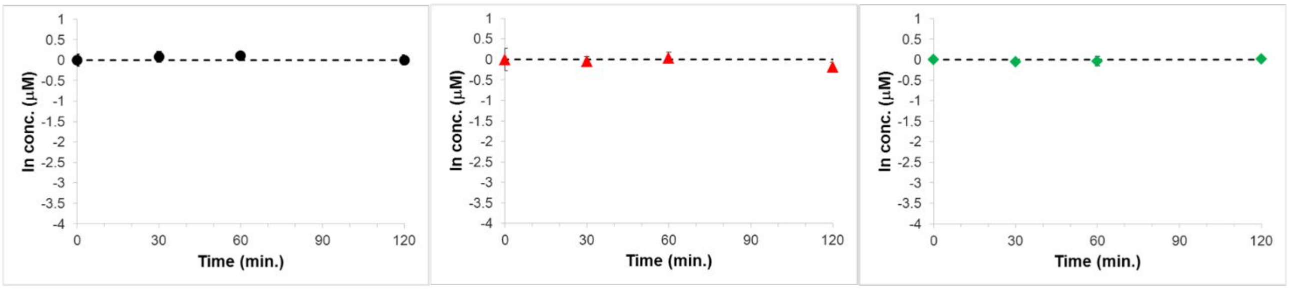

Figure S1: Degradation plots of **6** in PBS (blue square), human plasma (red triangle) and 10 mM $HCl_{(aq)}$ (green diamond).

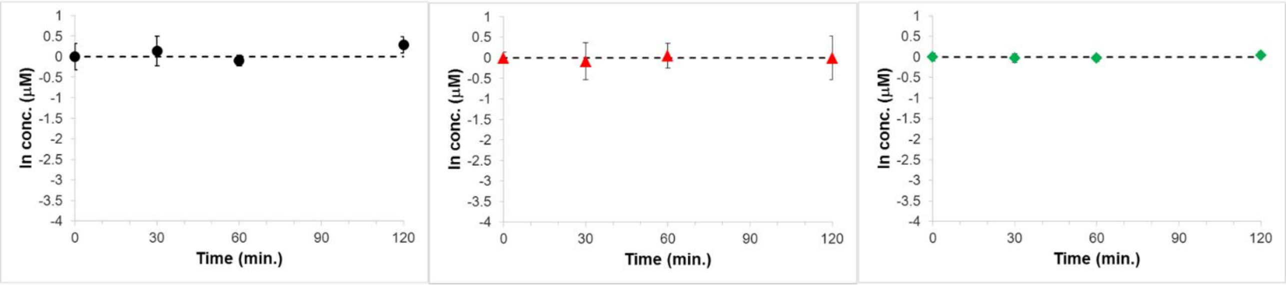

Figure S2: Degradation plots of **7** in PBS (blue square), human plasma (red triangle) and 10 mM $HCl_{(aq)}$ (green diamond).

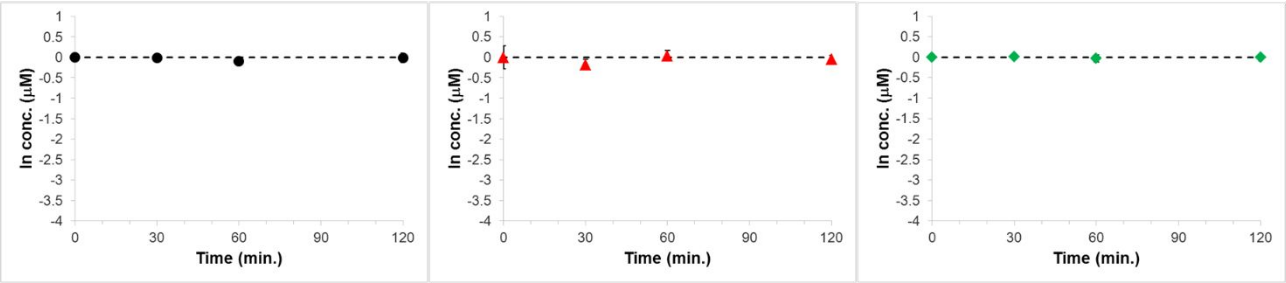

Figure S3: Degradation plots of **10** in PBS (blue square), human plasma (red triangle) and 10 mM $HCl_{(aq)}$ (green diamond).

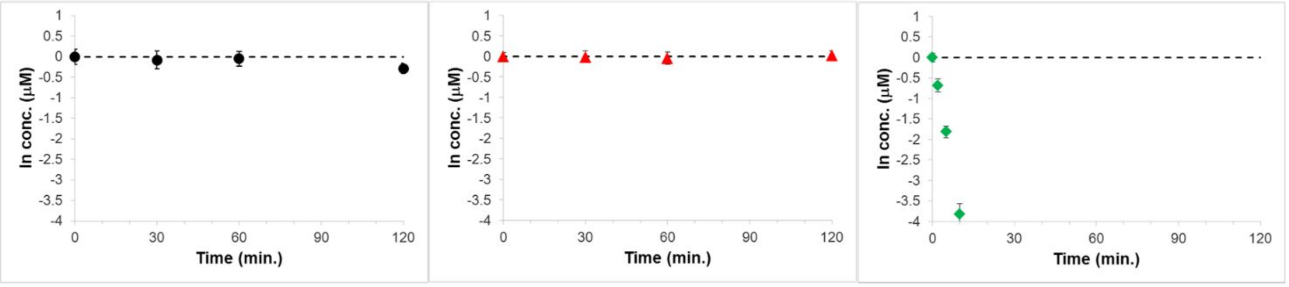

Figure S4: Degradation plots of **18** in PBS (blue square), human plasma (red triangle) and 10 mM $HCl_{(aq)}$ (green diamond).

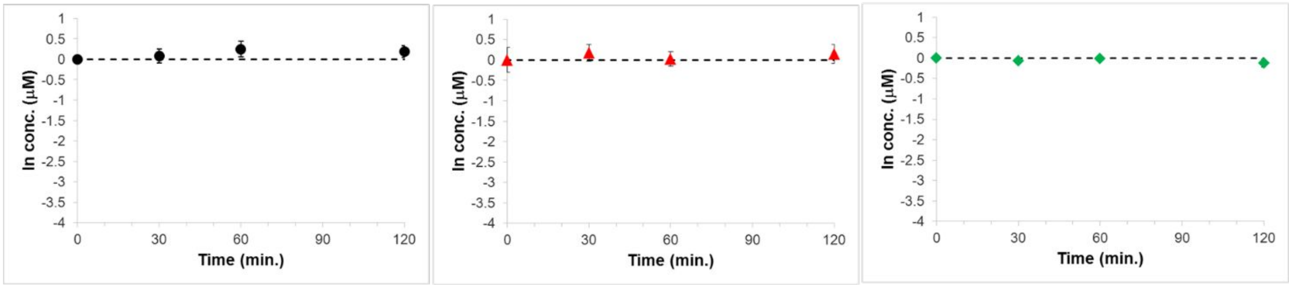

Figure S5: Degradation plots of **20** in PBS (blue square), human plasma (red triangle) and 10 mM HCl (green diamond).

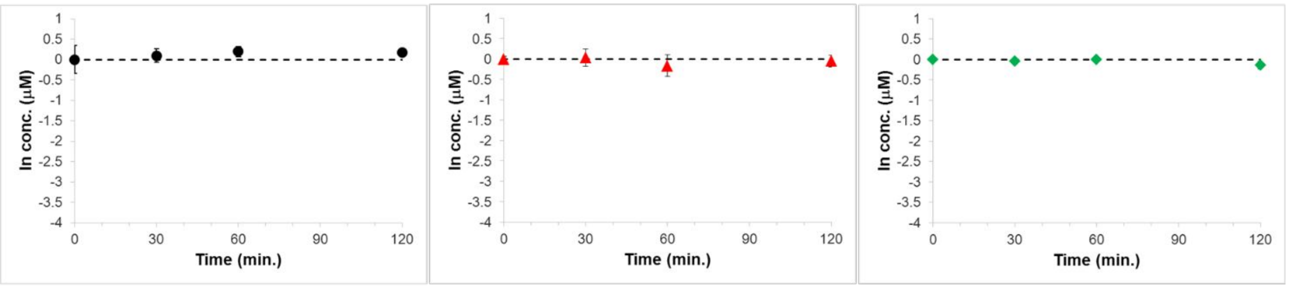

Figure S6: Degradation plots of **22** in PBS (blue square), human plasma (red triangle) and 10 mM $HCl_{(aq)}$ (green diamond).

HPLC chromatograms

Table S3. Elution gradient of mobile phase used for LC-MS/MSDAD analysis

Time (min)	A (%)
0.00	90
8.00	10
13.00	10
13.01	90
18.00	90

The chromatographic profiles of LC-DAD analysis and a representative UV spectrum are reported in Figures S7-27.

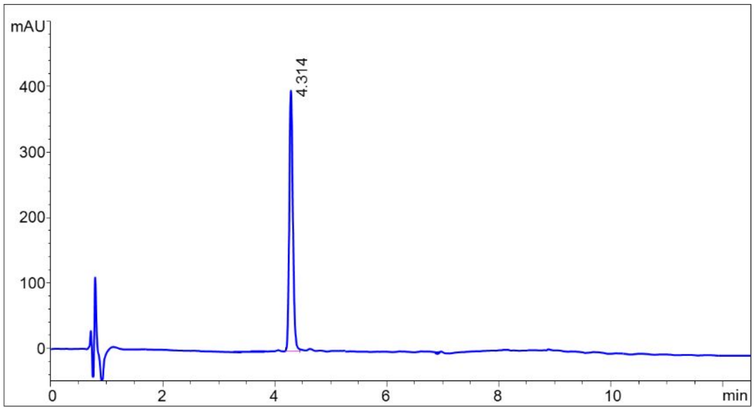

Figure S7. Chromatographic profile of **3** monitored at λ=230 nm.

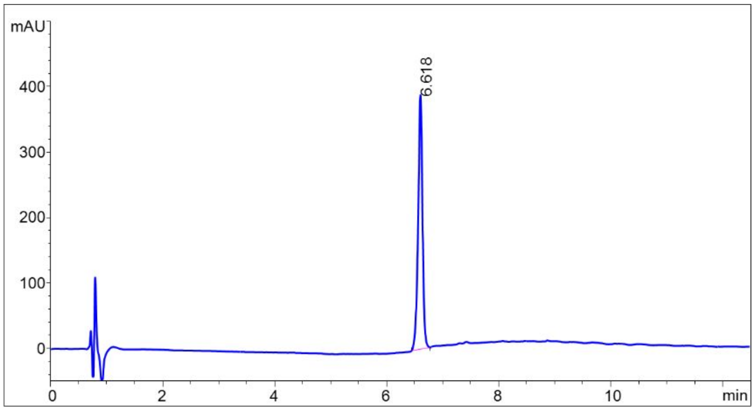

Figure S8. Chromatographic profile of **4** monitored at λ=230 nm.

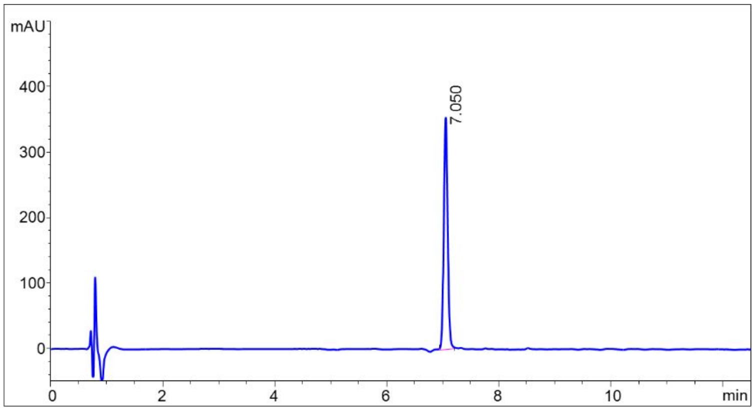

Figure S9. Chromatographic profile of **5** monitored at λ=230 nm.

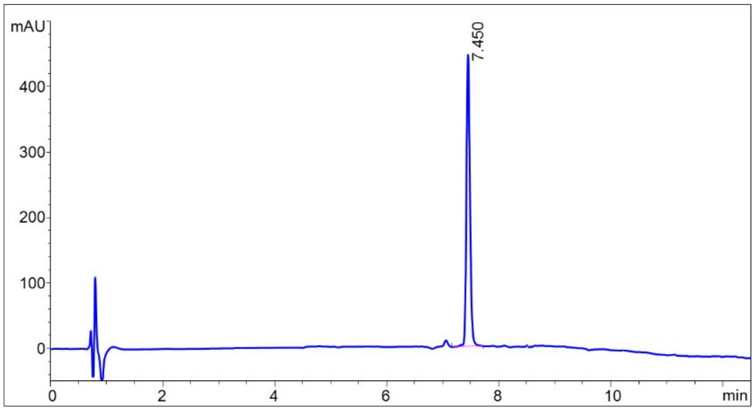

Figure S10. Chromatographic profile of **6** monitored at λ=230 nm.

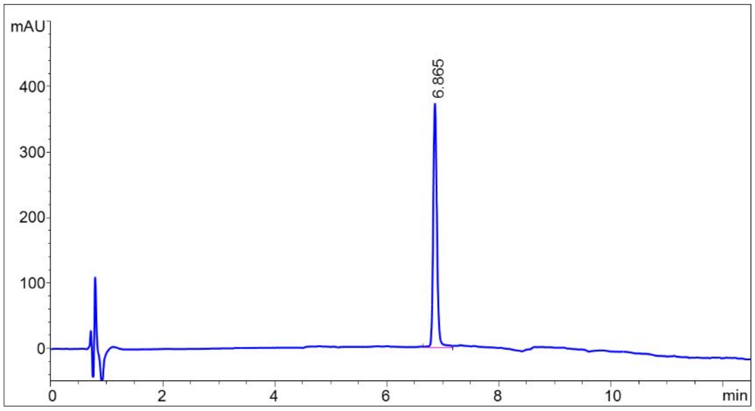

Figure S11. Chromatographic profile of **7** monitored at λ=230 nm.

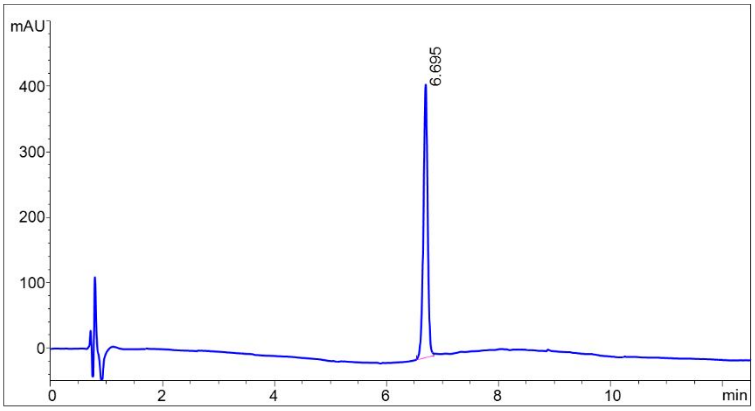

Figure S12. Chromatographic profile of **8** monitored at λ=230 nm.

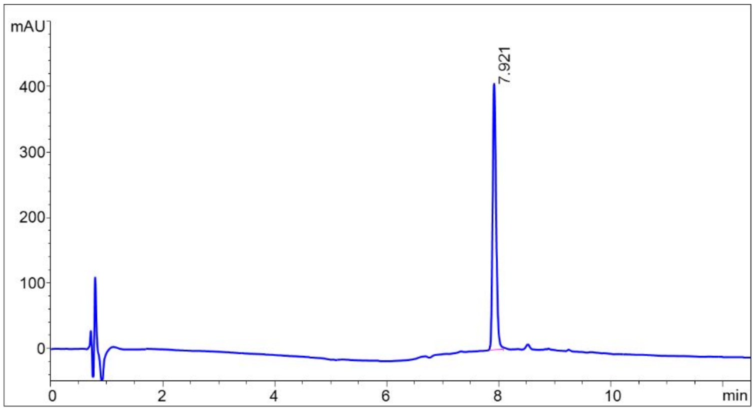

Figure S13. Chromatographic profile of **9** monitored at λ=230 nm.

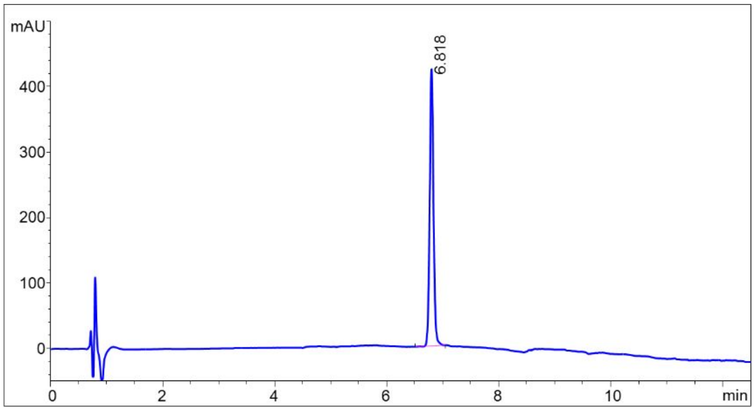

Figure S14. Chromatographic profile of **10** monitored at λ=230 nm.

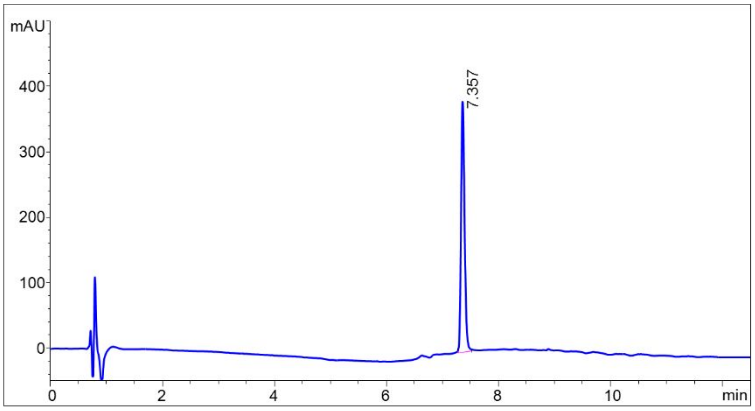

Figure S15. Chromatographic profile of **11** monitored at λ=230 nm.

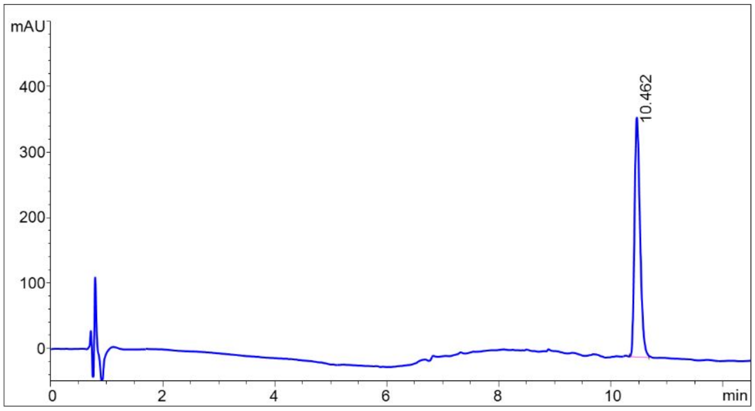

Figure S16. Chromatographic profile of **12** monitored at λ=230 nm.

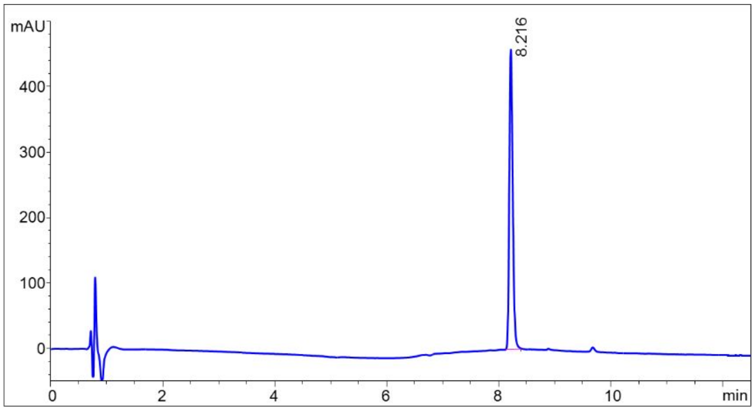

Figure S17. Chromatographic profile of **13** monitored at λ=230 nm.

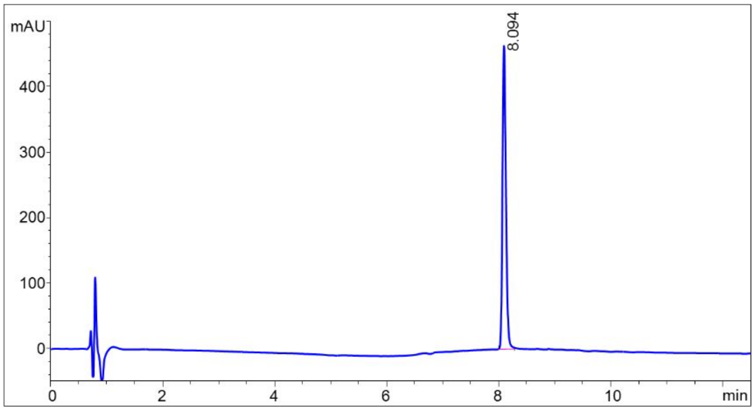

Figure S18. Chromatographic profile of **14** monitored at λ=230 nm.

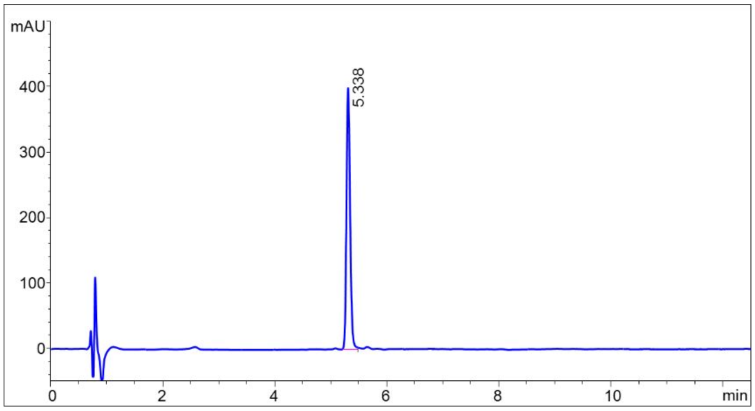

Figure S19. Chromatographic profile of **15** monitored at λ=230 nm.

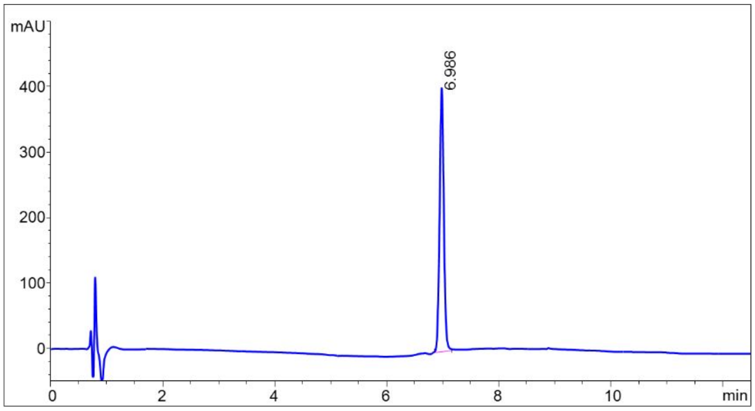

Figure S20. Chromatographic profile of **16** monitored at λ=230 nm.

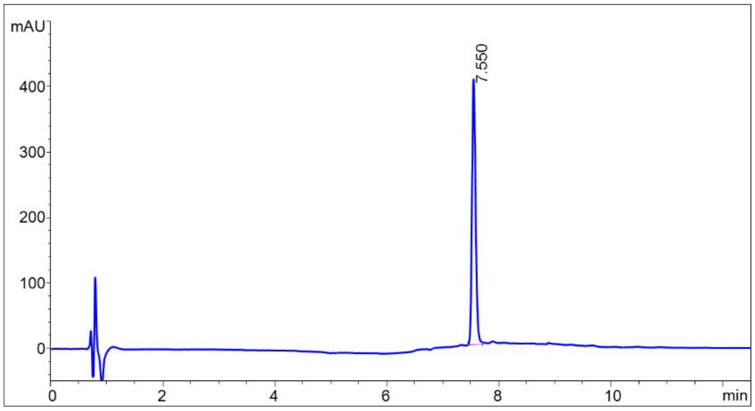

Figure S21. Chromatographic profile of **17** monitored at λ=230 nm.

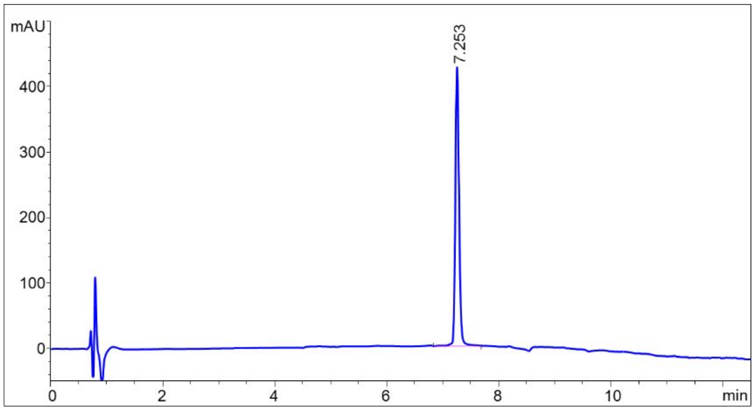

Figure S22. Chromatographic profile of **18** monitored at λ=230 nm.

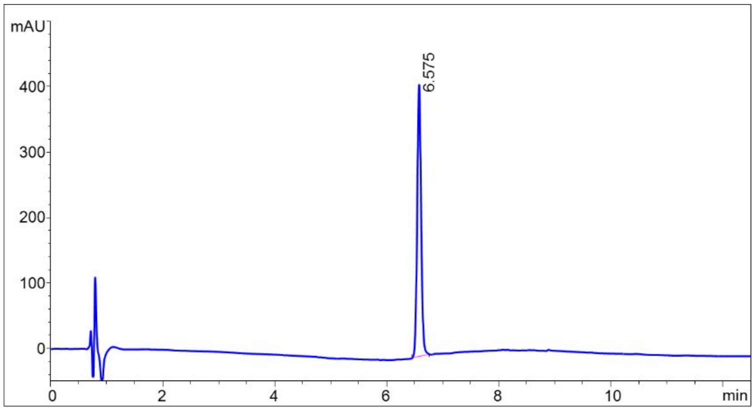

Figure S23. Chromatographic profile of **19** monitored at λ=230 nm.

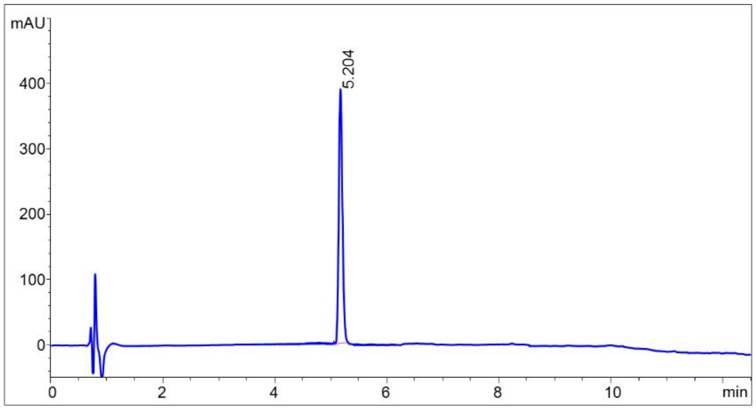

Figure S24. Chromatographic profile of **20** monitored at λ=230 nm.

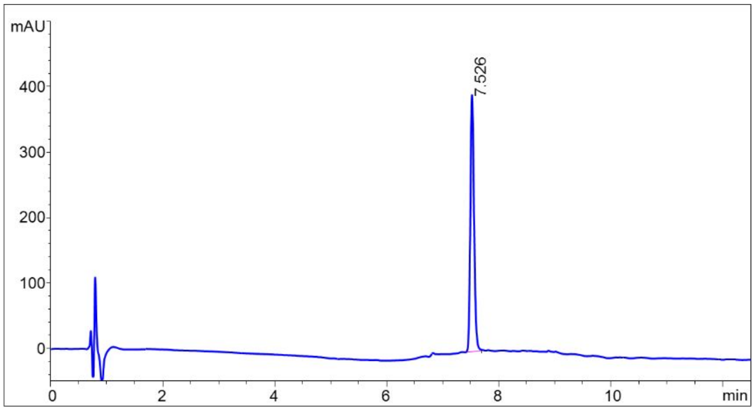

Figure S25. Chromatographic profile of **21** monitored at λ=230 nm.

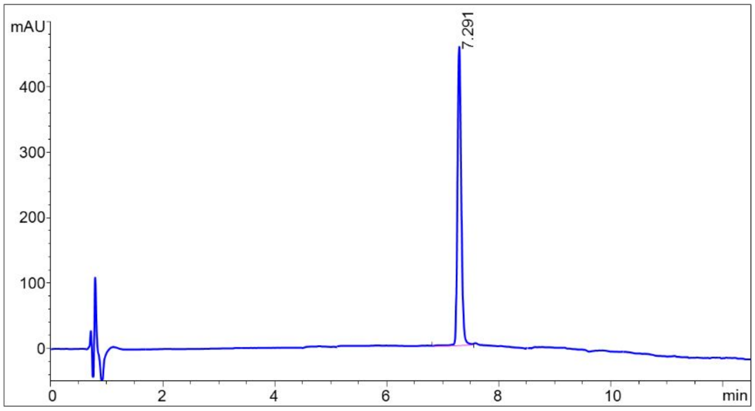

Figure S26. Chromatographic profile of **22** monitored at λ=230 nm.

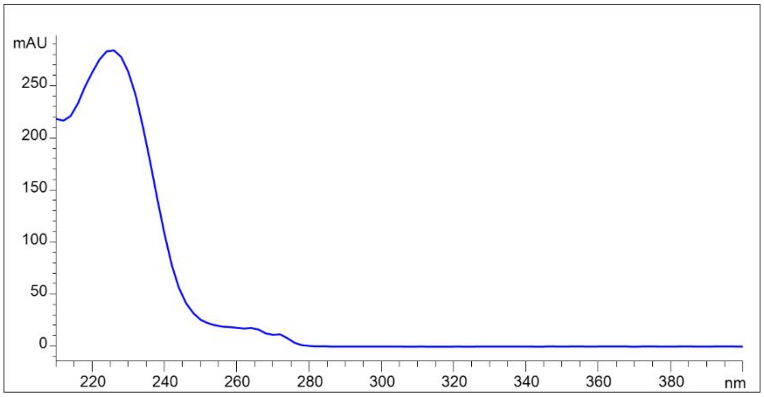

Figure S27. UV spectrum of the studied compounds.

Table S4. ***In silico*** **predicted ADMET properties for compounds tested** ***in vivo*** **(https://preadmet.bmdrc.kr/)**

Parameter	6	7	10	20	22
BBB[a]	0.116709	0.0487032	0.0539688	0.0511972	0.0147186
Buffer_solubility_mg_L	801.419**	1531.03**	74.23**	1801.69**	60.1026**
Caco2[b]	6.05651	6.95995	0.369409	20.0919	3.34751
CYP_2C19_inhibition	Inhibitor	Inhibitor	Inhibitor	Non	Inhibitor
CYP_2C9_inhibition	Inhibitor	Inhibitor	Inhibitor	Inhibitor	Inhibitor
CYP_2D6_inhibition	Non	Non	Non	Inhibitor	Non
CYP_2D6_substrate	Non	Non	Non	Substrate	Non
CYP_3A4_inhibition	Inhibitor	Inhibitor	Inhibitor	Non	Non
CYP_3A4_substrate	Substrate	Substrate	Substrate	Weakly	Substrate
HIA[c]	95.953475	96.446599	90.962078	97.427527	94.877979
MDCK[d]	19.1949	3.86873	0.0629787	12.987	1.07594
Pgp_inhibition	Non	Non	Inhibitor	Non	Non
Plasma_Protein_Binding	98.844965	100	100	56.123787	100
Pure_water_solubility_mg_L	571.202	401.749	32.0863	5636.12	42.4008
Skin_Permeability (cm/hour)	-1.15826	-0.981716*	-0.847708*	-1.95397*	-1.14593*
SKlogD_value[e]	1.89879	1.47579	1.34163	-0.6571	2.43052
SKlogP_value[f]	1.89879	1.47579	1.34163	0.907360	2.43052
SKlogS_buffer[g]	-2.646790**	-2.360710**	-3.800070**	-2.341370**	-3.810360**
SKlogS_pure[h]	-2.79386	-2.94174	-4.16433	-1.84607	-3.96188
algae_at[i]	0.0707286	0.0610489	0.0349119	0.121669	0.0560762
Ames_test[j]	non-mutagen	mutagen	non-mutagen	mutagen	non-mutagen
Carcino_Mouse[k]	negative	negative	positive	negative	negative
Carcino_Rat[l]	negative	negative	negative	negative	negative
daphnia_at[m]	0.199535	0.115303	0.136445	0.402159	0.122753
hERG_inhibition[n]	low_risk	low_risk	ambiguous	ambiguous	ambiguous
medaka_at[o]	0.0703555	0.025001	0.0474358	0.296	0.0317767
minnow_at[p]	0.0130446	0.0056248	0.00812458	0.141998	0.0090334
TA100_10RLI[q]	negative	negative	negative	negative	negative
TA100_NA[r]	negative	negative	negative	negative	negative
TA1535_10RLI[s]	negative	negative	negative	positive	negative
TA1535_NA[t]	negative	positive	negative	negative	negative

a. *In vivo* blood-brain barrier penetration (C.brain/C.blood)
b. *In vitro* Caco-2 cell permeability (nm/sec)
c. Human intestinal absorption (HIA, %)
d. In vitro MDCK cell permeability (nm/sec)
e. SK logD in pH 7.4 (SK atomic types)
f. SK logP (SK atomic types)
g. SK logS in buffer system (SK atomic types, S: mol/L)

h. SK logS in pure water (SK atomic types, S: mol/L)
i. Acute algae toxicity
j. Ames test. PNAS 1973, 69, 3128-213
k. 2 years carcinogenicity bioassay in mouse
l. 2 years carcinogenicity bioassay in rat
m. Acute daphina toxicity
n. *In vitro* hERG inhibition
o. Acute fish toxicity (medaka)
p. Acute fish toxicity (minnow)
q. *In vitro* Ames test result in TA100 strain (Metabolic activation by rat liver homogenate)
r. *In vitro* Ames test result in TA100 strain (No metabolic activation)
s. *In vitro* Ames test result in TA1535 strain (Metabolic activation by rat liver homogenate)
t. *In vitro* Ames test result in TA1535 strain (No metabolic activation)
